# Supplementary material for: Ceftolozane-Tazobactam Combination Therapy Compared to Ceftolozane-Tazobactam Monotherapy for the Treatment of Severe Infections: A Systematic Review and Meta-Analysis
Source: Antibiotics (Basel). 2021 Jan 15;10(1):79. doi: 10.3390/antibiotics10010079 (PMC7830767; doi:10.3390/antibiotics10010079)
Supplement: Supplementary file 1 [file antibiotics-10-00079-s001.zip › supplementary tables_/Table S1.docx]

**Table S1 –** Record excluded (n=1142) and reasons

| **Year** | **Author** | **Title** | **DOI** | **Engaged** | **Included** | **Reason** |
| --- | --- | --- | --- | --- | --- | --- |
| 2006 |  | Ceftolozane and Tazobactam |  | NO | NO | BOOK |
| 2015 |  | Ceftolozane/Tazobactam (Zerbaxa)--a new intravenous antibiotic |  | NO | NO | BOOK |
| 2015 |  | New drugs: Drug news |  | NO | NO | NOT FOUND |
| 2016 |  |  | <http://www.embase.com/search/results?subaction=viewrecord&from=export&id=L626839491> | NO | NO | CONFERENCE PROCEEDINGS |
| 2016 |  | CADTH Rapid Response Reports |  | NO | NO | REVIEW |
| 2016 |  | Ceftolozane sulfate with tazobactam sodium | 10.18773/austprescr.2016.044 | NO | NO | REVIEW |
| 2017 |  | Correction: Physical compatibility of ceftolozane-tazobactam with selected i.v. drugs during simulated Y-site administration (American Journal of Health-System Pharmacy (2017) (e50)) | 10.2146/cor170002 | NO | NO | PHARMACOLOGICAL STUDY |
| 2018 |  | NICE Guidance - Complicated urinary tract infections: ceftolozane/tazobactam: © NICE (2016) Complicated urinary tract infections: ceftolozane/tazobactam | 10.1111/bju.14364 | NO | NO | GUIDELINES |
| 2012 | F. I. Abandeh, M. E. Drew and M. M. Sopirala | Carbapenem-hydrolyzing gram-negative bacteria: Current options for treatment and review of drugs in development | 10.2174/157489112799829675 | NO | NO | REVIEW |
| 2018 | A. L. K. Abia, E. Ubomba-Jaswa, C. Schmidt and M. A. Dippenaar | Where did they come from—Multi-drug resistant pathogenic Escherichia coli in a cemetery environment? | 10.3390/antibiotics7030073 | NO | NO | IN VITRO STUDY |
| 2019 | S. A. Abuhussain, C. Sutherland and D. Nicolau | Susceptibility of p aeruginosa to ceftolozane/tazobactam and ceftazidime/avibactam |  | NO | NO | IN VITRO STUDY |
| 2019 | S. A. Abuhussain, C. Sutherland and D. Nicolau | Antimicrobial susceptibility of pseudomonas aeruginosa isolates collected in ICU and non-ICU setting |  | NO | NO | IN VITRO STUDY |
| 2019 | V. Adamkova | The role of new antibiotics in intra-abdominal infections in the era of multi-resistant bacteria |  | NO | NO | REVIEW |
| 2018 | J. M. Aguado, J. T. Silva, M. Fernández-Ruiz, E. Cordero, J. Fortún, C. Gudiol, L. Martínez-Martínez, E. Vidal, L. Almenar, B. Almirante, R. Cantón, J. Carratalá, J. J. Caston, E. Cercenado, C. Cervera, J. M. Cisneros, M. G. Crespo-Leiro, V. Cuervas-Mons, J. Elizalde-Fernández, M. C. Fariñas, J. Gavaldà, M. J. Goyanes, B. Gutiérrez-Gutiérrez, D. Hernández, O. Len, R. López-Andujar, F. López-Medrano, P. Martín-Dávila, M. Montejo, A. Moreno, A. Oliver, A. Pascual, E. Pérez-Nadales, A. Román-Broto, R. San-Juan, D. Serón, A. Solé-Jover, M. Valerio, P. Muñoz and J. Torre-Cisneros | Management of multidrug resistant Gram-negative bacilli infections in solid organ transplant recipients: SET/GESITRA-SEIMC/REIPI recommendations | 10.1016/j.trre.2017.07.001 | NO | NO | REVIEW |
| 2019 | G. Aguilar, R. Ferriols, S. Martinez-Castro, C. Ezquer, E. Pastor, J. A. Carbonell, M. Alos and D. Navarro | Optimizing ceftolozane-tazobactam dosage in critically ill patients during continuous venovenous hemodiafiltration | 10.1186/s13054-019-2434-5 | NO | NO | CASE REPORT |
| 2020 | G. Aguilar, R. Ferriols, S. Martinez-Castro, C. Ezquer, E. Pastor, J. A. Carbonell, M. Alos and D. Navarro | Optimizing ceftolozane-tazobactam dosage during continuous renal replacement therapy: some nuances | 10.1186/s13054-019-2724-y | NO | NO | LETTER |
| 2020 | D. Aguilera-Alonso, L. Escosa-García, J. Saavedra-Lozano, E. Cercenado and F. Baquero-Artigao | Carbapenem-resistant Gram-negative bacterial infections in children | 10.1128/AAC.02183-19 | NO | NO | REVIEW |
| 2016 | S. L. Aitken, D. P. Kontoyiannis, A. M. Depombo, M. M. Bhatti, F. P. Tverdek, S. C. Gettys, D. P. Nicolau and C. A. Nunez | Use of ceftolozane/tazobactam in the treatment of multidrug-resistant pseudomonas aeruginosa bloodstream infection in a pediatric leukemia patient | 10.1097/INF.0000000000001228 | NO | NO | CASE REPORT |
| 2016 | A. Aiudi, B. Miller, G. Krishna, A. Adedoyin and A. Xiao | Pharmacokinetics, safety, and tolerability of ceftolozane/tazobactam in healthy Japanese, Chinese, and white subjects | 10.1111/fcp.12227 | NO | NO | PHARMACOLOGICAL STUDY |
| 2019 | F. Z. Akçam | Carbapenem-resistant enterobacteria: How to cope? | 10.5578/flora.68459 | NO | NO | REVIEW |
| 2020 | S. Akyuz, M. Parlak and H. Guducuoglu | [In-vitro Activity of Ceftolozane-Tazobactam in Combination with Various Antibiotics Against Multidrug-resistant Acinetobacter baumannii Isolated from Intensive Care Patients] | 10.5578/mb.68981 | NO | NO | IN VITRO STUDY |
| 2017 | A. Alatoom | Monotherapy of ceftazidime–avibactam and ceftolozane–tazobactam against multidrug-resistant organisms | 10.1016/j.ijid.2017.06.022 | NO | NO | LETTER |
| 2017 | A. Alatoom, H. Elsayed, K. Lawlor, L. AbdelWareth, R. El-Lababidi, L. Cardona, M. Mooty, M. F. Bonilla, A. Nusair and I. Mirza | Comparison of antimicrobial activity between ceftolozane–tazobactam and ceftazidime–avibactam against multidrug-resistant isolates of Escherichia coli, Klebsiella pneumoniae, and Pseudomonas aeruginosa | 10.1016/j.ijid.2017.06.007 | NO | NO | IN VITRO STUDY |
| 2019 | A. Albasanz-Puig, C. Gudiol, R. Parody, C. Tebe, M. Akova, R. Araos, A. Bote, A. S. Brunel, S. Calik, L. Drgona, E. Garcia, P. Hemmati, F. Herrera, K. Y. Ibrahim, B. Isler, S. Kanj, W. Kern, G. Maestro de la Calle, A. Manzur, J. I. Marin, I. Marquez-Gomez, P. Martin-Davila, M. Mikulska, J. M. Montejo, M. Montero, H. M. P. Morales, I. Morales, A. Novo, C. Oltolini, M. Peghin, J. L. Del Pozo, P. Puerta-Alcalde, I. Ruiz-Camps, O. R. Sipahi, R. Tilley, L. Yanez, M. Z. R. Gomes and J. Carratala | Impact of antibiotic resistance on outcomes of neutropenic cancer patients with Pseudomonas aeruginosa bacteraemia (IRONIC study): study protocol of a retrospective multicentre international study | 10.1136/bmjopen-2018-025744 | YES | NO | IN VITRO STUDY |
| 2020 | I. Alekseeva, S. Lob, M. Hackel, K. Young, M. Motyl and D. Sahm | Activity of ceftolozane/tazobactam and imipenem/relebactam against clinical P. aeruginosa isolates collected in the Middle East – SMART 2016-2018 | 10.1016/j.jiph.2020.01.075 | NO | NO | IN VITRO STUDY |
| 2018 | M. A. Alessa, T. A. Almangour, A. Alhossan, M. A. Alkholief, M. Alhokail and D. E. Tabb | Ceftolozane-tazobactam for the treatment of multidrug-resistant Pseudomonas aeruginosa pneumonia in a patient receiving intermittent hemodialysis | 10.2146/ajhp170056 | NO | NO | CASE REPORT |
| 2019 | W. Alfouzan, R. Dhar and D. Nicolau | In vitro activity of newer and conventional antimicrobial agents, including fosfomycin and colistin, against selected gram-negative bacilli in Kuwait | 10.1016/j.jiph.2018.10.023 | NO | NO | IN VITRO STUDY |
| 2018 | W. Alfouzan, R. Dhar and D. P. Nicolau | In vitro activity of newer and conventional antimicrobial agents, including fosfomycin and colistin, against selected gram-negative Bacilli in Kuwait | 10.3390/pathogens7030075 | NO | NO | IN VITRO STUDY |
| 2019 | S. S. Almarzoky Abuhussain, C. A. Sutherland and D. P. Nicolau | In vitro potency of antipseudomonal β-lactams against blood and respiratory isolates of P. Aeruginosa collected from US hospitals | 10.21037/jtd.2019.05.13 | NO | NO | IN VITRO STUDY |
| 2020 | S. S. Almarzoky Abuhussain, C. A. Sutherland and D. P. Nicolau | Single β-lactams versus combinations as empiric therapy for infections with Pseudomonas aeruginosa: assessing the in vitro susceptibility | 10.1080/23744235.2019.1673900 | NO | NO | IN VITRO STUDY |
| 2017 | F. Alvarez Lerma, R. Munoz Bermudez, S. Grau, M. P. Gracia Arnillas, L. Sorli, L. Recasens and M. Mico Garcia | Ceftolozane-tazobactam for the treatment of ventilator-associated infections by colistin-resistant Pseudomonas aeruginosa |  | NO | NO | CASE REPORT |
| 2017 | P. G. Ambrose, O. Lomovskaya, D. C. Griffith, M. N. Dudley and B. VanScoy | β-Lactamase inhibitors: what you really need to know | 10.1016/j.coph.2017.09.001 | NO | NO | REVIEW |
| 2017 | P. G. Ambrose, B. D. VanScoy, M. Trang, J. McCauley-Miller, H. Conde, S. M. Bhavnani, D. C. Alexander and L. V. Friedrich | Pharmacokinetics-pharmacodynamics of CB-618 in combination with cefepime, ceftazidime, ceftolozane, or meropenem: The pharmacological basis for a stand-alone β-lactamase inhibitor | 10.1128/AAC.00630-17 | NO | NO | PHARMACOLOGICAL STUDY |
| 2015 | A. N. Amin and D. Deruelle | Healthcare-associated infections, infection control and the potential of new antibiotics in development in the USA | 10.2217/fmb.15.33 | NO | NO | REVIEW |
| 2020 | D. Amore, Y. Pecoraro, C. Carillo, G. Russo, C. Poggi, M. Anile, A. Pagini, M. Bassi, S. Cagnetti, E. Mottola, F. G. D'Agostino, J. Vannucci, S. Mantovani, F. Pugliese, T. De Giacomo, E. A. Rendina, F. Venuta and D. Diso | Use of Ceftazidime-Avibactam and Ceftolozane-Tazobactam After Lung Transplantation | 10.1016/j.transproceed.2020.02.051 | NO | NO | CASE REPORT |
| 2016 | J. Y. Ang, N. Abdel-Haq, F. Zhu, A. K. Thabit, D. P. Nicolau, M. J. Satlin and D. Van Duin | Multidrug-resistant Pseudomonas aeruginosa infection in a child with cystic fibrosis | 10.1128/AAC.00705-16 | NO | NO | CASE REPORT |
| 2020 | J. Y. Ang, A. Arrieta, J. S. Bradley, Z. Zhang, B. Yu, M. L. Rizk, M. G. Johnson and E. G. Rhee | Ceftolozane/Tazobactam in Neonates and Young Infants: The Challenges of Collecting Pharmacokinetics and Safety Data in This Vulnerable Patient Population | 10.1055/s-0039-3402719 | NO | NO | THIS IS A SUBGROUP ANALYSIS OF A PHASE 1, NONCOMPARATIVE, OPEN-LABEL, MULTICENTER STUDY THAT CHARACTERIZED THE PK, SAFETY, AND TOLERABILITY OF A SINGLE INTRAVENOUS (IV) DOSE OF CEFTOLOZANE/TAZOBACTAM IN PEDIATRIC PATIENTS WITH PROVEN/SUSPECTED GRAM-NEGATIVE INFECTION OR RECEIVING PERIOPERATIVE PROPHYLAXIS. |
| 2019 | A. Antonelli, T. Giani, V. Di Pilato, E. Riccobono, G. Perriello, A. Mencacci and G. M. Rossolini | KPC-31 expressed in a ceftazidime/avibactam-resistant Klebsiella pneumoniae is associated with relevant detection issues | 10.1093/jac/dkz156 | NO | NO | LETTER |
| 2012 | P. C. Appelbaum | 2012 and beyond: Potential for the start of a second pre-antibiotic era? | 10.1093/jac/dks213 | NO | NO | OUT OF TOPIC |
| 2019 | J. Arca-Suarez, P. Fraile-Ribot, J. C. Vazquez-Ucha, G. Cabot, M. Martinez-Guitian, E. Lence, C. Gonzalez-Bello, A. Beceiro, M. Rodriguez-Iglesias, F. Galan-Sanchez, G. Bou and A. Oliver | Challenging Antimicrobial Susceptibility and Evolution of Resistance (OXA-681) during Treatment of a Long-Term Nosocomial Infection Caused by a Pseudomonas aeruginosa ST175 Clone | 10.1128/aac.01110-19 | NO | NO | CASE REPORT |
| 2017 | C. Årdal, H. S. Blix, J. Plahte and J. A. Røttingen | An antibiotic’s journey from marketing authorization to use, Norway | 10.2471/BLT.16.172874 | NO | NO | OUT OF TOPIC |
| 2019 | F. Arena, L. H. De Angelis, E. Maglioni, M. Contorni, M. I. Cassetta, A. Novelli and G. M. Rossolini | Ceftolozane-tazobactam pharmacokinetics during extracorporeal membrane oxygenation in a lung transplant recipient | 10.1128/AAC.02131-18 | NO | NO | CASE REPORT |
| 2018 | S. J. R. Arends, D. Shortridge, M. Castanheira, J. M. Streit and R. K. Flamm | Global surveillance: Susceptibility of ceftolozane-tazobactam against escherichia coli, Klebsiella pneumoniae, and pseudomonas aeruginosa isolates collected from bloodstream infections in the United States from 2015 to 2017 | 10.1093/ofid/ofy210.884 | NO | NO | IN VITRO STUDY |
| 2018 | S. J. R. Arends, D. Shortridge, L. R. Duncan, J. M. Streit and R. K. Flamm | Antimicrobial activity of ceftolozane-tazobactam tested against contemporary (2015-2017) gram-negative isolates from patients with pneumonia in US medical centers | 10.1093/ofid/ofy210.1314 | NO | NO | IN VITRO STUDY |
| 2020 | E. Argudo, J. Riera, S. Luque, I. Los-Arcos, M. Lopez-Meseguer, A. Sandiumenge, X. Nuvials, S. Grau and R. Ferrer | Effects of the extracorporeal membrane oxygenation circuit on plasma levels of ceftolozane | 10.1177/0267659119864813 | NO | NO | CASE REPORT |
| 2016 | A. Arizpe, K. R. Reveles, S. D. Patel and S. L. Aitken | Updates in the Management of Cephalosporin-Resistant Gram-Negative Bacteria | 10.1007/s11908-016-0552-7 | NO | NO | REVIEW |
| 2006 | R. Armer, P. Warne and J. Witherington | Recent disclosures of clinical drug candidates |  | NO | NO | CONFERENCE PROCEEDINGS |
| 2016 | E. S. Armstrong, D. J. Farrell, M. Palchak and J. N. Steenbergen | In Vitro activity of ceftolozane-tazobactam against anaerobic organisms identified during the ASPECT-cIAI study | 10.1128/AAC.01964-15 | YES | NO | IN VITRO STUDY |
| 2016 | E. S. Armstrong, J. A. Mikulca, D. J. Cloutier, C. A. Bliss and J. N. Steenbergen | Outcomes of high-dose levofloxacin therapy remain bound to the levofloxacin minimum inhibitory concentration in complicated urinary tract infections | 10.1186/s12879-016-2057-2 | YES | NO | NO C/T DATA |
| 2018 | T. E. Asempa, L. M. Avery, J. M. Kidd, J. L. Kuti and D. P. Nicolau | Physical compatibility of plazomicin with select i.v. Drugs during simulated Y-site administration | 10.2146/ajhp170839 | NO | NO | PHARMACOLOGICAL STUDY |
| 2018 | E. A. Ashley, J. Recht, A. Chua, D. Dance, M. Dhorda, N. V. Thomas, N. Ranganathan, P. Turner, P. J. Guerin, N. J. White and N. P. Day | An inventory of supranational antimicrobial resistance surveillance networks involving low- and middle-income countries since 2000 | 10.1093/jac/dky026 | NO | NO | OUT OF TOPIC |
| 2018 | L. B. S. Aulin, P. A. Valitalo, M. L. Rizk, S. A. G. Visser, G. Rao, P. H. van der Graaf and J. G. C. van Hasselt | Validation of a Model Predicting Anti-infective Lung Penetration in the Epithelial Lining Fluid of Humans | 10.1007/s11095-017-2336-7 | NO | NO | OUT OF TOPIC |
| 2019 | L. M. Avery, I. H. Chen, S. Reyes, D. P. Nicolau and J. L. Kuti | Assessment of the Physical Compatibility of Eravacycline and Common Parenteral Drugs During Simulated Y-site Administration | 10.1016/j.clinthera.2019.08.005 | NO | NO | OUT OF TOPIC |
| 2019 | L. M. Avery, C. A. Sutherland and D. P. Nicolau | In vitro investigation of synergy among fosfomycin and parenteral antimicrobials against carbapenemase-producing Enterobacteriaceae | 10.1016/j.diagmicrobio.2019.05.014 | NO | NO | IN VITRO STUDY |
| 2019 | L. M. Avery, C. A. Sutherland and D. P. Nicolau | Prevalence of in vitro synergistic antibiotic interaction between fosfomycin and nonsusceptible antimicrobials in carbapenemresistant Pseudomonas aeruginosa | 10.1099/jmm.0.000984 | NO | NO | IN VITRO STUDY |
| 2015 | S. Avin, A. Avinash, N. Patil, S. K. Kunder, A. Pathak and K. L. Bairy | Recent advances in the development of antibacterial agents |  | NO | NO | REVIEW |
| 2017 | C. Aye, M. Williams and R. Horvath | Multidrug Resistant Pseudomonas Mycotic Pseudoaneurysm following Cardiac Transplant Bridged by Ventricular Assistant Device | 10.1155/2017/1402320 | NO | NO | CASE REPORT |
| 2017 | R. Badal, G. Coombs, D. Drinkovic, J. Ellem, N. George, A. Hubber, T. Korman, I. Rapajic-Moran, S. Roberts and S. Taylor | Study for monitoring antimicrobial resistance trends (SMART) in Australia and New Zealand (ANZ), 2002-2016 |  | NO | NO | IN VITRO STUDY |
| 2017 | M. S. Bader, M. Loeb and A. A. Brooks | An update on the management of urinary tract infections in the era of antimicrobial resistance | 10.1080/00325481.2017.1246055 | NO | NO | REVIEW |
| 2019 | M. S. Bader, M. Loeb, D. Leto and A. A. Brooks | Treatment of urinary tract infections in the era of antimicrobial resistance and new antimicrobial agents | 10.1080/00325481.2019.1680052 | NO | NO | REVIEW |
| 2018 | A. L. Bailey, T. Armstrong, H. P. Dwivedi, G. A. Denys, J. Hindler, S. Campeau, M. Traczewski, R. Humphries and C. A. Burnham | Multicenter Evaluation of the Etest Gradient Diffusion Method for Ceftolozane-Tazobactam Susceptibility Testing of Enterobacteriaceae and Pseudomonas aeruginosa | 10.1128/jcm.00717-18 | NO | NO | OUT OF TOPIC |
| 2016 | T. M. Baker and M. J. Satlin | The growing threat of multidrug-resistant Gram-negative infections in patients with hematologic malignancies | 10.1080/10428194.2016.1193859 | NO | NO | REVIEW |
| 2019 | S. Baklouti, C. Massip, C. Mane, H. Guet-Revillet, M. Murris, D. Concordet and P. Gandia | Comment on: In vitro activity of seven β-lactams including ceftolozane/tazobactam and ceftazidime/avibactam against Burkholderia cepacia complex, Burkholderia gladioli and other non-fermentative Gram-negative bacilli isolated from cystic fibrosis patients | 10.1093/jac/dkz307 | NO | NO | IN VITRO STUDY |
| 2019 | A. S. Ballaben, R. Galetti, L. N. Andrade, J. C. Ferreira, D. de Oliveira Garcia, P. da Silva, Y. Doi and A. L. C. Darini | Plasmid Carrying blaCTX-M-2 and blaGES-1 in Extensively Drug-Resistant Pseudomonas aeruginosa from Cerebrospinal Fluid | 10.1128/AAC.00186-19 | NO | NO | IN VITRO STUDY |
| 2011 | R. Bals, D. Hubert and B. Tümmler | Antibiotic treatment of CF lung disease: From bench to bedside | 10.1016/S1569-1993(11)60019-2 | YES | NO | REVIEW |
| 2016 | M. Barbieri, D. Crocchiolo and M. A. Veneziano | Budget impact analysis of ceftolozane/tazobactam (zerbaxa) for the treatment of complicated intra-abdominal infections and complicated urinary tract infections in the Italian setting |  | YES | NO | THE AIM OF THIS STUDY IS TO ESTIMATE THE BUDGET IMPACT FOLLOWING THE INTRODUCTION OF ZERBAXA IN THE TREATMENT OF COMPLICATED INTRA-ABDOMINAL INFEC-TIONS (CIAIS) AND COMPLICATED URINARY TRACT INFECTIONS (CUTIS) BOTH FOR THE ITALIAN NATIONAL HEALTH SERVICE (NHS) AND AT A REGIONAL LEVEL. |
| 2019 | J. R. Bariola, T. Khadem and M. H. Nguyen | Uptake and initial use of novel antimicrobial agents at community hospitals in western Pennsylvania | 10.1017/ice.2019.220 | YES | NO | WE UNDER-TOOK A REVIEW OF THE INITIAL USE OF 7 NEW ANTIMICROBIAL AGENTS(ORITAVANCIN, DALBAVANCIN, TEDIZOLID, CEFTOLOZANE-TAZOBACTAM, CEF-TAZIDIME-AVIBACTAM, PERAMIVIR, AND ISAVUCONOZONIUM) AFTERFOOD AND DRUG ADMINISTRATION (FDA) APPROVAL AT 6 COMMUNITYHOSPITALS IN WESTERN PENNSYLVAN |
| 2018 | G. Barlow | Clinical challenges in antimicrobial resistance | 10.1038/s41564-018-0121-y | NO | NO | COMMENT |
| 2018 | M. D. Barnes, M. A. Taracila, J. D. Rutter, C. R. Bethel, I. Galdadas, A. M. Hujer, E. Caselli, F. Prati, J. P. Dekker, K. M. Papp-Wallace, S. Haider and R. A. Bonomo | Deciphering the evolution of cephalosporin resistance to ceftolozane-tazobactam in pseudomonas aeruginosa | 10.1128/mBio.02085-18 | YES | NO | OUT OF TOPIC |
| 2017 | M. D. Barnes, M. L. Winkler, M. A. Taracila, M. G. Page, E. Desarbre, B. N. Kreiswirth, R. K. Shields, M. H. Nguyen, C. Clancy, B. Spellberg, K. M. Papp-Wallace and R. A. Bonomo | Klebsiella pneumoniae carbapenemase-2 (KPC-2), substitutions at ambler position Asp179, and resistance to ceftazidime-avibactam: Unique antibiotic-resistant phenotypes emerge from β-lactamase protein engineering | 10.1128/mBio.00528-17 | NO | NO | OUT OF TOPIC |
| 2015 | S. L. Barriere | Clinical, economic and societal impact of antibiotic resistance | 10.1517/14656566.2015.983077 | NO | NO | EDITORIAL |
| 2018 | M. Bartoletti, M. Giannella, S. Tedeschi and P. Viale | Multidrug-Resistant Bacterial Infections in Solid Organ Transplant Candidates and Recipients | 10.1016/j.idc.2018.04.004 | NO | NO | REVIEW |
| 2018 | A. Bass, R. Echols, S. Portsmouth and A. Howell | Heterogeneity of recent phase 3 CUTI clinical trials with new antibiotics | 10.1093/ofid/ofy210.1350 | YES | NO | ABSTRACT |
| 2018 | M. Bassetti, M. Akova and M. Tumbarello | Treatment and mortality of Klebslella pneumoniae infections in critically ill patients: should we do and predict them better? | 10.1007/s00134-018-5390-7 | NO | NO | EDITORIAL |
| 2013 | M. Bassetti, M. Merelli, C. Temperoni and A. Astilean | New antibiotics for bad bugs: Where are we? | 10.1186/1476-0711-12-22 | NO | NO | REVIEW |
| 2016 | M. Bassetti, D. Pecori and M. Peghin | Multidrug-resistant Gram-negative bacteria-resistant infections: Epidemiology, clinical issues and therapeutic options | 10.4081/itjm.2016.802 | NO | NO | REVIEW |
| 2019 | M. Bassetti, M. Peghin, A. Vena and D. R. Giacobbe | Treatment of Infections Due to MDR Gram-Negative Bacteria | 10.3389/fmed.2019.00074 | NO | NO | REVIEW |
| 2015 | M. Bassetti and E. Righi | Development of novel antibacterial drugs to combat multiple resistant organisms | 10.1007/s00423-015-1280-4 | NO | NO | REVIEW |
| 2015 | M. Bassetti and E. Righi | Ceftolozane/tazobactam for the treatment of complicated urinary tract and intra-abdominal infections | 10.2217/fmb.14.112 | YES | NO | REVIEW |
| 2016 | M. Bassetti, E. Righi and A. Carnelutti | New therapeutic options for respiratory tract infections | 10.1097/QCO.0000000000000251 | NO | NO | REVIEW |
| 2018 | M. Bassetti, E. Righi, A. Russo and A. Carnelutti | New Antibiotics for Pneumonia | 10.1016/j.ccm.2018.08.007 | NO | NO | REVIEW |
| 2018 | M. Bassetti, A. Russo, A. Carnelutti, A. La Rosa and E. Righi | Antimicrobial resistance and treatment: an unmet clinical safety need | 10.1080/14740338.2018.1488962 | NO | NO | REVIEW |
| 2018 | M. Bassetti, A. Vena, N. Castaldo, E. Righi and M. Peghin | New antibiotics for ventilator-associated pneumonia | 10.1097/QCO.0000000000000438 | NO | NO | REVIEW |
| 2018 | M. Bassetti, A. Vena, A. Croxatto, E. Righi and B. Guery | How to manage Pseudomonas aeruginosa infections | 10.7573/dic.212527 | NO | NO | REVIEW |
| 2018 | M. Bassetti, A. Vena, A. Russo, A. Croxatto, T. Calandra and B. Guery | Rational approach in the management of Pseudomonas aeruginosa infections | 10.1097/QCO.0000000000000505 | NO | NO | REVIEW |
| 2015 | M. Berrazeg, K. Jeannot, V. Y. Ntsogo Enguene, I. Broutin, S. Loeffert, D. Fournier and P. Plesiat | Mutations in beta-Lactamase AmpC Increase Resistance of Pseudomonas aeruginosa Isolates to Antipseudomonal Cephalosporins | 10.1128/aac.00825-15 | NO | NO | IN VITRO STUDY |
| 2015 | E. Bettiol and S. Harbarth | Development of new antibiotics: Taking off finally? | 10.4414/smw.2015.14167 | NO | NO | REVIEW |
| 2018 | M. R. Bidell and T. P. Lodise | Suboptimal Clinical Response Rates with Newer Antibiotics Among Patients with Moderate Renal Impairment: Review of the Literature and Potential Pharmacokinetic and Pharmacodynamic Considerations for Observed Findings | 10.1002/phar.2184 | NO | NO | REVIEW |
| 2019 | L. Blanchard-Jacquet, R. Meghnagi, M. Lafaurie, B. Bercot, I. Madelaine and S. Touratier |  | 10.1016/j.medmal.2019.04.151 | YES | NO | ABSTRACT |
| 2016 | O. Blennow and P. Ljungman | The challenge of antibiotic resistance in haematology patients | 10.1111/bjh.13816 | NO | NO | REVIEW |
| 2017 | K. G. Blumenthal, P. G. Wickner, S. Hurwitz, N. Pricco, A. E. Nee, K. Laskowski, E. S. Shenoy and R. P. Walensky | Tackling inpatient penicillin allergies: Assessing tools for antimicrobial stewardship | 10.1016/j.jaci.2017.02.005 | NO | NO | OUT OF TOPIC |
| 2018 | M. Boattini, G. Bianco, L. Biancone, R. Cavallo and C. Costa | Ralstonia mannitolilytica bacteraemia: A case report and literature review |  | NO | NO | CASE REPORT |
| 2019 | E. C. Bodo, A. Caffrey, V. Lopes, J. A. Cusumano, L. A. Puzniak, E. McCann and K. LaPlante | Evaluation of multidrug-resistant pseudomonas aeruginosa isolates in patients receiving ceftolozane/tazobactam | 10.1093/ofid/ofz360.1969 | YES | NO | IN VITRO STUDY |
| 2014 | J. R. Bogner | New antibiotics prior to approval: Is this the end of the innovative stagnation? | 10.1007/s00108-014-3616-5 | NO | NO | REVIEW |
| 2019 | R. A. Bonomo and S. R. Evans | Reply to Humphrey and Spafford | 10.1093/cid/ciz228 | NO | NO | LETTER |
| 2013 | H. W. Boucher, G. H. Talbot, D. K. Benjamin, J. Bradley, R. J. Guidos, R. N. Jones, B. E. Murray, R. A. Bonomo and D. Gilbert | 10 × '20 progress - Development of new drugs active against gram-negative bacilli: An update from the infectious diseases society of America | 10.1093/cid/cit152 | NO | NO | REVIEW |
| 2019 | T. Boulant, A. B. Jousset, R. A. Bonnin, A. Barrail-Tran, A. Borgel, S. Oueslati, T. Naas and L. Dortet | A 2.5-Year Within-Patient Evolution of Pseudomonas aeruginosa Isolates with in Vivo Acquisition of Ceftolozane-Tazobactam and Ceftazidime-Avibactam Resistance upon Treatment | 10.1128/AAC.01637-19 | NO | NO | CASE REPORT |
| 2019 | M. Bour, D. Fournier, T. Jove, A. Pouzol, G. Miltgen, F. Janvier, K. Jeannot and P. Plesiat | Acquisition of class C beta-lactamase PAC-1 by ST664 strains of Pseudomonas aeruginosa | 10.1128/aac.01375-19 | NO | NO | CASE REPORT |
| 2018 | H. Bouxom, D. Fournier, K. Bouiller, D. Hocquet and X. Bertrand | Which non-carbapenem antibiotics are active against extended-spectrum β-lactamase-producing Enterobacteriaceae? | 10.1016/j.ijantimicag.2018.03.014 | NO | NO | IN VITRO STUDY |
| 2019 | D. A. Boyd, L. F. Lisboa, R. Rennie, G. G. Zhanel, T. C. Dingle and M. R. Mulvey | Identification of a novel metallo-β-lactamase, CAM-1, in clinical Pseudomonas aeruginosa isolates from Canada | 10.1093/jac/dkz066 | NO | NO | IN VITRO STUDY |
| 2015 | T. Boyles | Ceftolozane-tazobactam versus levofloxacin in urinary tract infection | 10.1016/S0140-6736(15)00260-3 | NO | NO | LETTER |
| 2018 | J. S. Bradley, J. Y. Ang, A. C. Arrieta, K. B. Larson, M. L. Rizk, L. Caro, S. Yang, B. Yu, M. G. Johnson and E. G. Rhee | Pharmacokinetics and Safety of Single Intravenous Doses of Ceftolozane/Tazobactam in Children with Proven or Suspected Gram-Negative Infection | 10.1097/INF.0000000000002170 | YES | NO | PHARMACOLOGICAL STUDY |
| 2016 | D. N. Bremmer, D. P. Nicolau, P. Burcham, A. Chunduri, G. Shidham and K. A. Bauer | Ceftolozane/Tazobactam Pharmacokinetics in a Critically Ill Adult Receiving Continuous Renal Replacement Therapy | 10.1002/phar.1744 | NO | NO | CASE REPORT |
| 2014 | C. Bretonniere, D. Boutoille, J. Caillon, C. Desessard, C. Guitton, G. Potel and C. Jacqueline | In vivo efficacy of ceftolozane against Pseudomonas aeruginosa in a rabbit experimental model of pneumonia: comparison with ceftazidime, piperacillin/tazobactam and imipenem | 10.1016/j.ijantimicag.2014.04.017 | NO | NO | CASE REPORT |
| 2020 | F. Briano, L. Magnasco, F. Toscanini, F. Dodi, R. Ungaro, A. Marchese and C. Viscoli | A Case Report of Bacterial Peritonitis Caused by MBL-Producing Citrobacter werkmanii in Peritoneal Dialysis | 10.1007/s42399-019-00192-0 | NO | NO | CASE REPORT |
| 2018 | R. K. Brookman, A. Harrington, J. A. Hindler, M. Traczewski, S. Campeau, S. DesJarlais, D. Beasley, C. J. Hastey and D. Roe-Carpenter | Multicenter evaluation of ceftolozane/tazobactam MIC results for enterobacteriaceae and pseudomonas aeruginosa using microscan dried gram negative MIC Panels |  | NO | NO | IN VITRO STUDY |
| 2015 | D. Brown | Antibiotic resistance breakers: Can repurposed drugs fill the antibiotic discovery void? | 10.1038/nrd4675 | NO | NO | REVIEW |
| 2020 | P. Brown and M. Battistella | Principles of drug dosing in sustained low efficiency dialysis (SLED) and review of antimicrobial dosing literature | 10.3390/pharmacy8010033 | NO | NO | REVIEW |
| 2016 | D. J. Buehrle, R. K. Shields, L. Chen, B. Hao, E. G. Press, A. Alkrouk, B. A. Potoski, B. N. Kreiswirth, C. J. Clancy and M. H. Nguyen | Evaluation of the in vitro activity of ceftazidime-avibactam and ceftolozane-tazobactam against meropenem-resistant Pseudomonas aeruginosa isolates | 10.1128/AAC.02969-15 | NO | NO | IN VITRO STUDY |
| 2010 | C. C. Bulik, H. Christensen and D. P. Nicolau | In vitro potency of CXA-101, a novel cephalosporin, against Pseudomonas aeruginosa displaying various resistance phenotypes, including multidrug resistance | 10.1128/AAC.00912-09 | NO | NO | IN VITRO STUDY |
| 2012 | C. C. Bulik, P. R. Tessier, R. A. Keel, C. A. Sutherland and D. P. Nicolau | In vivo comparison of CXA-101 (FR264205) with and without tazobactam versus piperacillin-tazobactam using human simulated exposures against phenotypically diverse gram-negative organisms | 10.1128/AAC.01752-10 | NO | NO | PRECLINICAL STUDY |
| 2017 | Z. P. Bulman, L. Chen, T. J. Walsh, M. J. Satlin, Y. Qian, J. B. Bulitta, C. A. Peloquin, P. N. Holden, R. L. Nation, J. Li, B. N. Kreiswirth and B. T. Tsuji | Polymyxin combinations combat Escherichia coli harboring mcr-1 and blaNDM-5: Preparation for a postantibiotic Era | 10.1128/mBio.00540-17 | NO | NO | OUT OF TOPIC |
| 2020 | A. R. Buonomo, A. E. Maraolo, R. Scotto, M. Foggia, E. Zappulo, P. Congera, S. Parente and I. Gentile | Efficacy and safety of ceftolozane/tazobactam as therapeutic option for complicated skin and soft tissue infections by MDR/XDR Pseudomonas aeruginosa in patients with impaired renal function: a case series from a single-center experience | 10.1007/s15010-020-01390-y | NO | NO | CASE SERIES |
| 2019 | A. Burillo, V. de Egea, R. Onori, P. Martin-Rabadan, E. Cercenado, L. Jimenez-Navarro, P. Munoz and E. Bouza | Gradient diffusion antibiogram used directly on bronchial aspirates for a rapid diagnosis of ventilator-associated pneumonia | 10.1186/s13756-019-0640-1 | NO | NO | IN VITRO STUDY |
| 2017 | J. P. Burnham, S. T. Micek and M. H. Kollef | Augmented renal clearance is not a risk factor for mortality in Enterobacteriaceae bloodstream infections treated with appropriate empiric antimicrobials | 10.1371/journal.pone.0180247 | YES | NO | OUT OF TOPIC |
| 2006 | J. Burt | Developments in antibacterial compounds. 16-19 December 2005, Washington, DC, USA |  | NO | NO | BOOK |
| 2012 | K. Bush | Improving known classes of antibiotics: An optimistic approach for the future | 10.1016/j.coph.2012.06.003 | NO | NO | REVIEW |
| 2015 | K. Bush | A resurgence of β-lactamase inhibitor combinations effective against multidrug-resistant Gram-negative pathogens | 10.1016/j.ijantimicag.2015.08.011 | NO | NO | REVIEW |
| 2018 | K. Bush | Synergistic antibiotic combinations | 10.1007/7355_2017_23 | NO | NO | REVIEW |
| 2010 | K. Bush and M. J. MacIelag | New β-lactam antibiotics and β-lactamase inhibitors | 10.1517/13543776.2010.515588 | NO | NO | REVIEW |
| 2017 | K. Bush and M. G. P. Page | What we may expect from novel antibacterial agents in the pipeline with respect to resistance and pharmacodynamic principles | 10.1007/s10928-017-9506-4 | NO | NO | REVIEW |
| 2019 | D. A. Butler, M. Biagi, X. Tan, S. Qasmieh, Z. P. Bulman and E. Wenzler | Multidrug Resistant Acinetobacter baumannii: Resistance by Any Other Name Would Still be Hard to Treat | 10.1007/s11908-019-0706-5 | NO | NO | REVIEW |
| 2013 | M. S. Butler, M. A. Blaskovich and M. A. Cooper | Antibiotics in the clinical pipeline in 2013 | 10.1038/ja.2013.86 | NO | NO | REVIEW |
| 2017 | M. S. Butler, M. A. Blaskovich and M. A. Cooper | Antibiotics in the clinical pipeline at the end of 2015 | 10.1038/ja.2016.72 | NO | NO | REVIEW |
| 2011 | M. S. Butler and M. A. Cooper | Antibiotics in the clinical pipeline in 2011 | 10.1038/ja.2011.44 | NO | NO | REVIEW |
| 2013 | J. D. Buynak | β-Lactamase inhibitors: A review of the patent literature (2010 - 2013) | 10.1517/13543776.2013.831071 | NO | NO | REVIEW |
| 2018 | V. R. Caballero, S. A. Abuhussain, J. L. Kuti and D. P. Nicolau | Efficacy of human-simulated exposures of ceftolozane-tazobactam alone and in combination with amikacin or colistin against multidrug-resistant pseudomonas aeruginosa in an in vitro pharmacodynamic model | 10.1128/AAC.02384-17 | NO | NO | IN VITRO STUDY |
| 2014 | G. Cabot, S. Bruchmann, X. Mulet, L. Zamorano, B. Moya, C. Juan, S. Haussler and A. Oliver | Pseudomonas aeruginosa ceftolozane-tazobactam resistance development requires multiple mutations leading to overexpression and structural modification of AmpC | 10.1128/aac.02462-13 | NO | NO | IN VITRO STUDY |
| 2018 | G. Cabot, L. Florit-Mendoza, I. Sánchez-Diener, L. Zamorano and A. Oliver | Deciphering β-lactamase-independent β-lactam resistance evolution trajectories in Pseudomonas aeruginosa | 10.1093/jac/dky364 | NO | NO | IN VITRO STUDY |
| 2019 | N. Cabrera, T. T. Tran, W. R. Miller, A. Q. Dinh, B. Hanson, J. M. Munita, S. A. Shelburne, S. L. Aitken, K. W. Garey, L. A. Puzniak and C. A. Arias | Clinical and microbiological outcomes associated with real-world use of ceftolozane/tazobactam | 10.1093/ofid/ofz360.1926 | YES | NO | IN VITRO STUDY |
| 2015 | D. J. Cada, D. E. Baker and R. J. Bindler | Ledipasvir/sofosbuvir | 10.1310/hpj5003-224 | NO | NO | MONOGRAPH |
| 2015 | D. J. Cada, J. Wageman, D. E. Baker and T. L. Levien | Ceftolozane/tazobactam | 10.1310/hpj5006-526 | NO | NO | MONOGRAPH |
| 2018 | K. M. Caflisch, S. M. Schmidt-Malan, J. N. Mandrekar, M. J. Karau, J. P. Nicklas, L. B. Williams and R. Patel | Antibacterial activity of reduced iron clay against pathogenic bacteria associated with wound infections | 10.1016/j.ijantimicag.2018.07.018 | NO | NO | OUT OF TOPIC |
| 2018 | J. Campanini-Salinas, J. Andrades-Lagos, J. Mella-Raipan and D. Vásquez-Velásquez | Novel classes of antibacterial drugs in clinical development, a hope in a post-antibiotic era | 10.2174/1568026618666180816162846 | NO | NO | REVIEW |
| 2017 | F. J. Candel González, M. Peñuelas, C. Lejárraga, T. Emilov, C. Rico, I. Díaz, C. Lázaro, J. M. Viñuela-Prieto and M. Matesanz | Update in infectious diseases 2017 |  | NO | NO | REVIEW |
| 2012 | R. Cantón and J. Bryan | Global antimicrobial resistance: from surveillance to stewardship. Part 2: Stewardship initiatives | 10.1586/eri.12.140 | NO | NO | CONFERENCE PROCEEDINGS |
| 2020 | R. Cantón, A. Oliver, J. I. Alós, N. de Benito, G. Bou, J. Campos, J. Calvo, A. Canut, J. Castillo, E. Cercenado, M. Á Domínguez, F. Fernández-Cuenca, J. Guinea, N. Larrosa, J. Liñares, L. López-Cerero, A. López-Navas, F. Marco, B. Mirelis, M. Á Moreno-Romo, M. I. Morosini, F. Navarro, J. Oteo, Á Pascual, E. Pérez-Trallero, M. Pérez-Vázquez, A. Soriano, C. Torres, J. Vila and L. Martínez-Martínez | Recommendations of the Spanish Antibiogram Committee (COESANT) for selecting antimicrobial agents and concentrations for in vitro susceptibility studies using automated systems | 10.1016/j.eimc.2019.01.017 | NO | NO | REVIEW |
| 2019 | M. L. Cantón-Bulnes, Á Hurtado Martínez, L. López-Cerero, Á Arenzana Seisdedos, V. Merino-Bohorquez and J. Garnacho-Montero | A case of pan-resistant Burkholderia cepacia complex bacteremic pneumonia, after lung transplantation treated with a targeted combination therapy | 10.1111/tid.13034 | NO | NO | CASE REPORT |
| 2019 | N. Carbonell, G. Aguilar, R. Ferriols, R. Huerta, J. Ferreres, M. Calabuig, M. Juan, C. Ezquer, J. Colomina and M. L. Blasco | Ceftolozane Pharmacokinetics in a Septic Critically Ill Patient under Different Extracorporeal Replacement Therapies | 10.1128/aac.01782-19 | NO | NO | CASE REPORT |
| 2016 | L. Caro, K. Larson, D. Nicolau, J. DeWaele, J. Kuti, E. Gadzicki, B. Yu and E. Rhee | PK/PD and safety of 3 G ceftolozane/tazobactam in critically ill augmented renal clearance patients | 10.1097/01.ccm.0000509338.57932.11 | YES | NO | PHARMACOLOGICAL STUDY |
| 2020 | L. Caro, D. P. Nicolau, J. J. De Waele, J. L. Kuti, K. B. Larson, E. Gadzicki, B. Yu, Z. Zeng, A. Adedoyin and E. G. Rhee | Lung penetration, bronchopulmonary pharmacokinetic/pharmacodynamic profile and safety of 3 g of ceftolozane/tazobactam administered to ventilated, critically ill patients with pneumonia | 10.1093/jac/dkaa049 | YES | NO | PHARMACOLOGICAL STUDY |
| 2019 | C. G. Carvalhaes, M. Castanheira, R. E. Mendes and H. S. Sader | Antimicrobial activity of ceftazidime-avibactam and comparator agents tested against gram-negative organisms isolated from patients with bloodstream infections in United States medical centers (2017-2018) | 10.1093/ofid/ofz360.204 | NO | NO | IN VITRO STUDY |
| 2019 | C. G. Carvalhaes, M. Castanheira, H. S. Sader, R. K. Flamm and D. Shortridge | Antimicrobial activity of ceftolozane–tazobactam tested against gram-negative contemporary (2015–2017) isolates from hospitalized patients with pneumonia in US medical centers | 10.1016/j.diagmicrobio.2018.11.021 | YES | NO | IN VITRO STUDY |
| 2019 | C. G. Carvalhaes, R. E. Mendes, R. K. Flamm and H. S. Sader | Comparative antimicrobial susceptibility of gram-negative bacteria isolated from patients with bloodstream infections and pneumonia when tested against tazobactam combinations | 10.1093/ofid/ofz360.1872 | NO | NO | IN VITRO STUDY |
| 2017 | N. Castaldo, F. Givone, M. Peghin, E. Righi, A. Sartor and M. Bassetti | Multidrug-resistant Pseudomonas aeruginosa skin and soft-tissue infection successfully treated with ceftolozane/tazobactam | 10.1016/j.jgar.2017.02.012 | NO | NO | CASE REPORT |
| 2019 | M. Castanheira, T. B. Doyle, C. Hubler, M. Healy, C. J. Smith and R. E. Mendes | Î²-lactam resistance mechanisms in pseudomonas aeruginosa isolates analyzed using whole-genome sequencing (WGS) and transcriptions analysis and their impact in resistance to new Î²-lactam/Î²-lactamase inhibitors | 10.1093/ofid/ofz360.669 | NO | NO | IN VITRO STUDY |
| 2019 | M. Castanheira, T. B. Doyle, R. E. Mendes and H. S. Sader | Comparative activities of ceftazidime-avibactam and ceftolozane-tazobactam against Enterobacteriaceae isolates producing extended-spectrum -lactamases from U.S. Hospitals | 10.1128/AAC.00160-19 | NO | NO | IN VITRO STUDY |
| 2018 | M. Castanheira, L. R. Duncan, R. E. Mendes, H. S. Sader and D. Shortridge | Activity of ceftolozane-tazobactam against pseudomonas aeruginosa and enterobacteriaceae isolates collected from respiratory tract specimens of hospitalized patients in the United States during 2013 to 2015 | 10.1128/AAC.02125-17 | NO | NO | IN VITRO STUDY |
| 2016 | M. Castanheira, M. A. Griffin, L. M. Deshpande, R. E. Mendes, R. N. Jones and R. K. Flamm | Detection of mcr-1 among Escherichia coli clinical isolates collected worldwide as part of the SENTRY Antimicrobial Surveillance Program in 2014 and 2015 | 10.1128/AAC.01267-16 | NO | NO | IN VITRO STUDY |
| 2019 | M. Castanheira, M. D. Huband, R. K. Flamm and H. S. Sader | Antimicrobial activity of the novel β-lactam enhancer combination cefepime-zidebactam (WCK-5222) tested against gram-negative bacteria isolated in United States medical centers from patients with bloodstream infections | 10.1093/ofid/ofz360.1462 | NO | NO | IN VITRO STUDY |
| 2019 | M. Castanheira, M. G. Johnson, B. Yu, J. A. Huntington, P. Carmelitano, C. Bruno, E. G. Rhee and M. Motyl | Molecular characterization of baseline enterobacteriaceae and pseudomonas aeruginosa from a phase 3 nosocomial pneumonia (ASPECT-NP) clinical trial | 10.1093/ofid/ofz360.692 | YES | NO | IN VITRO STUDY |
| 2019 | M. Castanheira, J. Lindley, H. Huynh, R. E. Mendes and O. Lomovskaya | Activity of novel β-lactamase inhibitor QPX7728 combined with β-lactam agents when tested against carbapenem-resistant enterobacteriaceae (CRE) isolates | 10.1093/ofid/ofz360.745 | NO | NO | IN VITRO STUDY |
| 2014 | M. Castanheira, J. C. Mills, D. J. Farrell and R. N. Jones | Mutation-Driven β-Lactam resistance mechanisms among contemporary ceftazidime-nonsusceptible pseudomonas aeruginosa isolates from U.S. hospitals | 10.1128/AAC.03681-14 | NO | NO | IN VITRO STUDY |
| 2017 | J. J. Caston, A. De la Torre, I. Ruiz-Camps, M. L. Sorli, V. Torres and J. Torre-Cisneros | Salvage Therapy with Ceftolozane-Tazobactam for Multidrug-Resistant Pseudomonas aeruginosa Infections | 10.1128/aac.02136-16 | YES | NO | CASE SERIES |
| 2013 | V. Cattoir | Infections caused by resistant gram-negative bacilli: New molecules and combinations | 10.1016/j.antinf.2013.09.001 | NO | NO | REVIEW |
| 2019 | L. J. Caverly, T. Spilker, L. M. Kalikin, T. Stillwell, C. Young, D. B. Huang and J. J. LiPuma | In Vitro Activities of beta-Lactam-beta-Lactamase Inhibitor Antimicrobial Agents against Cystic Fibrosis Respiratory Pathogens | 10.1128/aac.01595-19 | NO | NO | IN VITRO STUDY |
| 2016 | E. Cerceo, S. B. Deitelzweig, B. M. Sherman and A. N. Amin | Multidrug-Resistant Gram-Negative Bacterial Infections in the Hospital Setting: Overview, Implications for Clinical Practice, and Emerging Treatment Options | 10.1089/mdr.2015.0220 | NO | NO | REVIEW |
| 2016 | W. Chaijamorn, A. Shaw, S. J. Lewis and B. Mueller | Ceftolozane/tazobactam clearance during in vitro continuous renal replacement therapy (CRRT) | 10.1093/ofid/ofw172.1515 | NO | NO | IN VITRO STUDY |
| 2017 | W. Chaijamorn, A. R. Shaw, S. J. Lewis and B. A. Mueller | Ex vivo Ceftolozane/Tazobactam Clearance during Continuous Renal Replacement Therapy | 10.1159/000455897 | YES | NO | PRECLINICAL STUDY |
| 2015 | H. F. Chambers | Challenging resistance |  | NO | NO | REVIEW |
| 2018 | E. Chan, P. Martelli, S. W. Hui, J. L. L. Teng, S. K. P. Lau and P. C. Y. Woo | In Vitro Susceptibility of Ceftolozane-Tazobactam against Burkholderia pseudomallei | 10.1128/aac.00103-18 | NO | NO | IN VITRO STUDY |
| 2019 | E. Chan, A. K. L. Wu, C. W. S. Tse, S. K. P. Lau and P. C. Y. Woo | In vitro susceptibility of Salmonella enterica serovar Typhi to ceftolozane/tazobactam | 10.1093/jac/dky418 | NO | NO | IN VITRO STUDY |
| 2015 | J. F. W. Chan and K. Y. Yuen | A new ASPECT for complicated urinary tract infections | 10.1016/S0140-6736(14)62482-X | YES | NO | COMMENT |
| 2012 | G. Chandorkar, J. Huntington, T. Parsons, M. Gotfried and O. Umeh | Penetration of ceftolozane/tazobactam and piperacillin/ tazobactam into the epithelial lining fluid of healthy volunteers | 10.1111/j.1469-0691.2012.03802.x | YES | NO | OUT OF TOPIC |
| 2012 | G. Chandorkar, J. A. Huntington, M. H. Gotfried, K. A. Rodvold and O. Umeh | Intrapulmonary penetration of ceftolozane/tazobactam and piperacillin/tazobactam in healthy adult subjects | 10.1093/jac/dks246 | YES | NO | OUT OF TOPIC |
| 2013 | G. Chandorkar, S. Mouksassi, E. Hershberger and G. Krishna | Population pharmacokinetic analysis of ceftolozane/tazobactam in healthy volunteers and patients | 10.1002/phar.1356 | NO | NO | PHARMACOLOGICAL STUDY |
| 2014 | G. Chandorkar, A. Xiao, M. S. Mouksassi, E. Hershberger and G. Krishna | Population pharmacokinetics of ceftolozane/tazobactam in healthy volunteers, subjects with varying degrees of renal function and patients with bacterial infections | 10.1002/jcph.395 | YES | NO | PHARMACOLOGICAL STUDY |
| 2017 | K. Chellapandi, T. K. Dutta, I. Sharma, S. Mandal, N. S. Kumar and L. Ralte | Prevalence of multi drug resistant enteropathogenic and enteroinvasive Escherichia coli isolated from children with and without diarrhea in Northeast Indian population | 10.1186/s12941-017-0225-x | YES | NO | IN VITRO STUDY |
| 2019 | C. Chen, Y. Zhang, S. L. Yu, Y. Zhou, S. Y. Yang, J. L. Jin, S. Chen, P. Cui, J. Wu, N. Jiang and W. H. Zhang | Tracking Carbapenem-Producing Klebsiella pneumoniae Outbreak in an Intensive Care Unit by Whole Genome Sequencing | 10.3389/fcimb.2019.00281 | NO | NO | IN VITRO STUDY |
| 2019 | I. H. Chen, J. M. Kidd, K. Abdelraouf and D. P. Nicolau | Comparative in vivo antibacterial activity of human-simulated exposures of cefiderocol and ceftazidime against stenotrophomonas maltophilia in the murine thigh model | 10.1128/AAC.01558-19 | NO | NO | PRECLINICAL STUDY |
| 2018 | L. Chen and B. N. Kreiswirth | Convergence of carbapenem-resistance and hypervirulence in Klebsiella pneumoniae | 10.1016/S1473-3099(17)30517-0 | NO | NO | COMMENT |
| 2019 | L. Chen, Y. Su, L. Quan, Y. Zhang and L. Du | Clinical trials focusing on drug control and prevention of ventilator-associated pneumonia: A comprehensive analysis of trials registered on ClinicalTrials.gov | 10.3389/fphar.2018.01574 | NO | NO | OUT OF TOPIC |
| 2018 | M. Chen, M. Zhang, P. Huang, Q. Lin, C. Sun, H. Zeng and Y. Deng | Novel β-lactam/β-lactamase inhibitors versus alternative antibiotics for the treatment of complicated intra-abdominal infection and complicated urinary tract infection: a meta-analysis of randomized controlled trials | 10.1080/14787210.2018.1429912 | NO | NO | META-ANALYSIS |
| 2019 | X. J. C. Chen, K. Fong, D. Altshuler, Y. Dubrovskaya, E. Louie, N. Amoroso, R. Goldenberg and J. Papadopoulos | Evaluation of Pharmacy-Developed Antibiotic Desensitization Protocols | 10.1177/1060028018801959 | YES | NO | OUT OF TOPIC |
| 2020 | I. L. Cheng, Y. H. Chen, C. C. Lai and H. J. Tang | The use of ceftolozane-tazobactam in the treatment of complicated intra-abdominal infections and complicated urinary tract infections—A meta-analysis of randomized controlled trials | 10.1016/j.ijantimicag.2019.11.015 | NO | NO | META-ANALYSIS |
| 2016 | F. Chiarella, J. González-Del Castillo, F. J. Candel, E. J. García-Lamberechts, M. J. Núñez-Orantos, F. J. Martín-Sánchez, I. G. Marques, E. M. F. de Pinedo, L. P. Ordoño, V. Á Rodríguez, M. A. R. Núñez, J. M. López Á, M. T. Aparicio, N. R. L. Martínez, R. R. Alonso, J. G. Criado, E. R. Gorriz, M. B. Sabater, M. M. Portillo Cano, A. J. G. Buigues, I. H. Sanz, A. G. Querejeta, J. J. G. del Rio, J. L. O. Martínez, J. A. R. Guerrero, M. B. Araujo, F. L. Roca, J. M. P. Monzo, S. B. Camacho, F. L. Alfonsin, M. C. M. Navarro, E. G. L. Ros, J. M. F. Amez and E. M. Pérez | Key issues in the infected patient care in the Emergency Department |  | NO | NO | GUIDELINES |
| 2015 | J. C. Cho, M. A. Fiorenza and S. J. Estrada | Ceftolozane/Tazobactam: A Novel Cephalosporin/β-Lactamase Inhibitor Combination | 10.1002/phar.1609 | NO | NO | REVIEW |
| 2018 | K. Choi, A. T. Truong, S. T. Hall, L. B. Robinson and V. Huang | Prolonged use of novel meropenem-vaborbactam in carbape-nem-resistant enterobacteriaceae: A case report | 10.1002/jac5.1059 | NO | NO | CASE REPORT |
| 2014 | S. Chopra, E. D. Beaulieu and J. P. Malerich | Natural products as anti-infective agents | 10.2174/9781608058600114020005 | NO | NO | BOOK |
| 2018 | J. Cies, W. Moore, N. Gilliam, A. Enache and A. Chopra | Ceftolozane-tazobactam ex vivo pharmacokinetics in an extracorporeal membrane oxygenation circuit | 10.1097/01.ccm.0000528886.38266.01 | NO | NO | PHARMACOLOGICAL STUDY |
| 2020 | J. J. Cies, W. S. Moore, N. Giliam, T. Low, A. Enache and A. Chopra | Oxygenator impact on ceftolozane and tazobactam in extracorporeal membrane oxygenation circuits | 10.1097/PCC.0000000000002174 | NO | NO | TO DETERMINE THE OXYGENATOR IMPACT ON ALTERATIONS OF CEFTOLOZANE/TAZOBACTAM IN A CONTEMPORARY NEONATAL/PEDIATRIC (1/4-INCH) AND ADOLESCENT/ADULT (3/8-INCH) EXTRACORPOREAL MEMBRANE OXYGENATION CIRCUIT INCLUDING THE QUADROX-I OXYGENATOR (MAQUET, WAYNE, NJ). |
| 2019 | D. Clerici, C. Oltolini, R. Greco, F. Pavesi, F. Giglio, S. Mastaglio, F. Lorentino, L. Lazzari, P. Scarpellini, J. Peccatori, M. Bernardi, C. Corti, A. Castagna and F. Ciceri | Outcomes of infections by multi-drug resistant gram-negative bacteria in acute leukemia treated with high dose chemotherapy | https://www.embase.com/a/#/search/results?subaction=viewrecord&rid=1&page=1&id=L629699945 | YES | NO | CONFERENCE PROCEEDINGS |
| 2015 | D. Cluck, P. Lewis, B. Stayer, J. Spivey and J. Moorman | Ceftolozane-tazobactam: A new-generation cephalosporin | 10.2146/ajhp150049 | NO | NO | REVIEW |
| 2012 | A. R. M. Coates and G. Halls | Antibiotics in phase II and III clinical trials | 10.1007/978-3-642-28951-4-11 | NO | NO | REVIEW |
| 2010 | P. Cole and S. Vasiliou | Highlights from the 50th interscience conference on antimicrobial agents and chemotherapy (ICAAC) | 10.1358/dof.2010.035.012.1583180 | NO | NO | CONFERENCE PROCEEDINGS |
| 2009 | P. Cole, S. Vasiliou and H. Font | Anti-infective innovations: Highlights from the 49th Interscience Conference on Antimicrobial Agents And Chemotherapy (ICAAC) |  | NO | NO | CONFERENCE PROCEEDINGS |
| 2019 | M. Collura, S. Giordano, G. Traverso, L. Termini, F. Ficili, A. Ferlisi, O. Bologna, C. Iaria, E. Parisi, T. Pensabene, M. Bertolino and M. A. Orlando | Ceftolozane/tazobactam for the treatment of MDR Pseudomonas aeruginosa in a patient suffering for Cystic Fibrosis waiting for lung transplant | 10.1186/s13052-019-0631-0 | NO | NO | CASE REPORT |
| 2019 | C. A. Colque, P. E. Tomatis, A. G. Albarracín, G. Dotta, D. M. Moreno, G. Hedemann, A. J. Moyano, R. Bonomo, H. Johansen, S. Molin, A. J. Vila and A. M. Smania | Chronic use of β-lactams can select ampc mediated preresistance to last generation ceftolozane in Pseudomonas aeruginosa |  | NO | NO | REVIEW |
| 2016 | E. Concia, F. Mazzaferri and M. Cordioli | New antibiotic development: Barriers and opportunities | 10.4081/itjm.2016.790 | NO | NO | REVIEW |
| 2019 | A. Connolly, C. Keane, E. McKone and C. Gallagher | Treatment of adult cystic fibrosis patients with ceftolozane-tazobactam with a previous adverse drug reaction to piperacillin-tazobactam | 10.1016/S1569-1993(19)30407-2 | YES | NO | ABSTRACT |
| 2016 | K. A. Connor | Newer Intravenous Antibiotics in the Intensive Care Unit: Ceftaroline, Ceftolozane-Tazobactam, and Ceftazidime-Avibactam | 10.4037/aacnacc2016612 | YES | NO | REVIEW |
| 2020 | D. A. Contreras, S. P. Fitzwater, D. D. Nanayakkara, J. Schaenman, G. M. Aldrovandi, O. B. Garner and S. Yang | Coinfections of two strains of NDM-1- And OXA-232-coproducing Klebsiella pneumoniae in a kidney transplant patient | 10.1128/AAC.00948-19 | NO | NO | CASE REPORT |
| 2013 | W. A. Craig and D. R. Andes | In vivo activities of ceftolozane, a new cephalosporin, with and without tazobactam against Pseudomonas aeruginosa and Enterobacteriaceae, including strains with extended-spectrum β-lactamases, in the thighs of neutropenic mice | 10.1128/AAC.01590-12 | NO | NO | PRECLINICAL STUDY |
| 2019 | R. L. Crass, K. A. Rodvold, B. A. Mueller and M. P. Pai | Renal dosing of antibiotics: Are we jumping the gun? | 10.1093/cid/ciy790 | NO | NO | REVIEW |
| 2017 | J. Creighton, H. Heffernan and J. Howard | Isolation of seven distinct carbapenemase-producing Gram-negative organisms from a single patient | 10.1093/jac/dkw378 | YES | NO | CASE REPORT |
| 2020 | M. Criscuolo and E. M. Trecarichi | Ceftazidime/avibactam and ceftolozane/tazobactam for multidrug-resistant gram negatives in patients with hematological malignancies: Current experiences | 10.3390/antibiotics9020058 | NO | NO | REVIEW |
| 2020 | G. T. Cuba, G. Rocha-Santos, R. Cayo, A. P. Streling, C. S. Nodari, A. C. Gales, A. C. C. Pignatari, D. P. Nicolau and C. R. V. Kiffer | In vitro synergy of ceftolozane/tazobactam in combination with fosfomycin or aztreonam against MDR Pseudomonas aeruginosa | 10.1093/jac/dkaa095 | NO | NO | IN VITRO STUDY |
| 2019 | J. A. Cusumano, E. Bodo, V. Lopes, K. W. McConeghy, L. A. Puzniak, E. McCann, K. L. LaPlante and A. R. Caffrey | Ceftolozane-Tazobactam utilization in the national veterans affairs healthcare system | 10.1002/pds.4864 | YES | NO | DATA NOT RELEVANT |
| 2018 | D. Daigle, J. Hamrick, C. Chatwin, N. Kurepina, B. N. Kreiswirth, R. K. Shields, A. Oliver, C. J. Clancy, M. H. Nguyen, D. Pevear and L. Xerri | Cefepime/VNRX-5133 broad-spectrum activity is maintained against emerging KPC-and PDC-variants in multidrug-resistant k. pneumoniae and p. aeruginosa | 10.1093/ofid/ofy210.1201 | NO | NO | IN VITRO STUDY |
| 2017 | C. E. Dandoy, M. I. Ardura, G. A. Papanicolaou and J. J. Auletta | Bacterial bloodstream infections in the allogeneic hematopoietic cell transplant patient: New considerations for a persistent nemesis | 10.1038/bmt.2017.14 | NO | NO | REVIEW |
| 2017 | A. M. Dassner, C. Sutherland, J. Girotto and D. P. Nicolau | In vitro Activity of Ceftolozane/Tazobactam Alone or with an Aminoglycoside Against Multi-Drug-Resistant Pseudomonas aeruginosa from Pediatric Cystic Fibrosis Patients | 10.1007/s40121-016-0141-y | NO | NO | IN VITRO STUDY |
| 2017 | B. Davido, O. Senard, P. de Truchis, J. Salomon and A. Dinh | Monotherapy of ceftazidime–avibactam and ceftolozane–tazobactam: two effective antimicrobial agents against multidrug-resistant organisms except for NDM-1 isolates | 10.1016/j.ijid.2017.06.021 | NO | NO | LETTER |
| 2017 | S. E. Davis, J. Ham, J. Hucks, A. Gould, R. Foster, D. Nicolau and P. B. Bookstaver | Use of continuous infusion ceftolozane-tazobactam with therapeutic drug monitoring in a patient with cystic fibrosis: A case report | 10.1002/phar.2052 | NO | NO | CASE REPORT |
| 2018 | G. V. De Socio, F. Di Donato, R. Paggi, C. Gabrielli, A. Belati, G. Rizza, M. Savoia, A. Repetto, E. Cenci and A. Mencacci | Laboratory automation reduces time to report of positive blood cultures and improves management of patients with bloodstream infection | 10.1007/s10096-018-3377-5 | NO | NO | IN VITRO STUDY |
| 2013 | C. D. de Souza Mendes and A. M. de Souza Antunes | Pipeline of known chemical classes of antibiotics | 10.3390/antibiotics2040500 | NO | NO | REVIEW |
| 2016 | J. J. De Waele | Abdominal Sepsis | 10.1007/s11908-016-0531-z | NO | NO | REVIEW |
| 2016 | D. Deak, K. Outterson, J. H. Powers and A. S. Kesselheim | Progress in the fight against multidrug-resistant bacteria? A review of U.S. food and drug administration-approved antibiotics, 2010-2015 | 10.7326/M16-0291 | NO | NO | REVIEW |
| 2017 | E. Del Barrio-Tofino, C. Lopez-Causape, G. Cabot, A. Rivera, N. Benito, C. Segura, M. M. Montero, L. Sorli, F. Tubau, S. Gomez-Zorrilla, N. Tormo, R. Dura-Navarro, E. Viedma, E. Resino-Foz, M. Fernandez-Martinez, C. Gonzalez-Rico, I. Alejo-Cancho, J. A. Martinez, C. Labayru-Echverria, C. Duenas, I. Ayestaran, L. Zamorano, L. Martinez-Martinez, J. P. Horcajada and A. Oliver | Genomics and Susceptibility Profiles of Extensively Drug-Resistant Pseudomonas aeruginosa Isolates from Spain | 10.1128/aac.01589-17 | NO | NO | IN VITRO STUDY |
| 2019 | E. Del Barrio-Tofino, I. Sanchez-Diener, L. Zamorano, S. Cortes-Lara, C. Lopez-Causape, G. Cabot, G. Bou, L. Martinez-Martinez and A. Oliver | Association between Pseudomonas aeruginosa O-antigen serotypes, resistance profiles and high-risk clones: results from a Spanish nationwide survey | 10.1093/jac/dkz346 | NO | NO | IN VITRO STUDY |
| 2019 | E. Del Barrio-Tofino, L. Zamorano, S. Cortes-Lara, C. Lopez-Causape, I. Sanchez-Diener, G. Cabot, G. Bou, L. Martinez-Martinez and A. Oliver | Spanish nationwide survey on Pseudomonas aeruginosa antimicrobial resistance mechanisms and epidemiology | 10.1093/jac/dkz147 | NO | NO | IN VITRO STUDY |
| 2016 | V. Del Bono and D. R. Giacobbe | Bloodstream infections in internal medicine | 10.1080/21505594.2016.1140296 | NO | NO | REVIEW |
| 2019 | P. Del Giacomo, A. R. Losito and M. Tumbarello | The role of carbapenem-resistant pathogens in cSSTI and how to manage them | 10.1097/QCO.0000000000000528 | NO | NO | REVIEW |
| 2020 | M. Delgado-Valverde, M. D. C. Conejo, L. Serrano, F. Fernandez-Cuenca and A. Pascual | Activity of cefiderocol against high-risk clones of multidrug-resistant Enterobacterales, Acinetobacter baumannii, Pseudomonas aeruginosa and Stenotrophomonas maltophilia | 10.1093/jac/dkaa117 | NO | NO | IN VITRO STUDY |
| 2019 | A. J. Denisuik, L. A. Garbutt, A. R. Golden, H. J. Adam, M. Baxter, K. A. Nichol, P. Lagace-Wiens, A. J. Walkty, J. A. Karlowsky, D. J. Hoban, M. R. Mulvey and G. G. Zhanel | Antimicrobial-resistant pathogens in Canadian ICUs: results of the CANWARD 2007 to 2016 study | 10.1093/jac/dky477 | NO | NO | IN VITRO STUDY |
| 2019 | D. DePestel, S. Lob, D. Hoban, K. Young, M. Motyl, J. Raddatz and D. Sahm | Ceftolozane-tazobactam and imipenem-relebactam in vitro activity vs. MDR P aeruginosa from U.S. ICUs |  | NO | NO | IN VITRO STUDY |
| 2018 | Z. Z. Deris and S. Kumar | Rational use of intravenous polymyxin B and colistin: A review |  | NO | NO | REVIEW |
| 2017 | S. Deshayes, A. Coquerel and R. Verdon | Neurological Adverse Effects Attributable to β-Lactam Antibiotics: A Literature Review | 10.1007/s40264-017-0578-2 | NO | NO | REVIEW |
| 2019 | M. D. Dibirov, N. N. Khachatryan, A. I. Isaev, G. S. Karsotyan, E. E. Alimova and E. A. Kostyuk | New possibilities in antibacterial treatment of intra-abdominal infections caused by multiresistant microflora | 10.17116/hirurgia201912174 | NO | NO | NOT IN ENGLISH |
| 2015 | Z. C. Dietch, P. M. Shah and R. G. Sawyer | Advances in Intra-abdominal Sepsis: What Is New? | 10.1007/s11908-015-0497-2 | NO | NO | REVIEW |
| 2018 | B. Dietl, I. Sanchez, P. Arcenillas, E. Cuchi, L. Gomez, F. J. Gonzalez de Molina, L. Boix-Palop, J. Nicolas and E. Calbo | Ceftolozane/tazobactam in the treatment of osteomyelitis and skin and soft-tissue infections due to extensively drug-resistant Pseudomonas aeruginosa: clinical and microbiological outcomes | 10.1016/j.ijantimicag.2017.11.003 | YES | NO | CASE SERIES |
| 2017 | G. Dimopoulos and M. Akova | An overview on severe infections in Europe | 10.1007/s00134-016-4650-7 | NO | NO | REVIEW |
| 2017 | A. Dinh, B. Davido, R. Calin, J. Paquereau, C. Duran, F. Bouchand, V. Phe, E. Chartier-Kastler, M. Rottman, J. Salomon, P. Plesiat and A. Potron | Ceftolozane/tazobactam for febrile UTI due to multidrug-resistant Pseudomonas aeruginosa in a patient with neurogenic bladder | 10.1038/scsandc.2017.19 | NO | NO | CASE REPORT |
| 2017 | A. Dinh, B. Wyplosz, S. Kerneis, D. Lebeaux, F. Bouchand, C. Duran, G. Beraud, P. Lazaro, B. Davido, S. Henard, E. Canoui, T. Ferry and M. Wolff | Use of ceftolozane/tazobactam as salvage therapy for infections due to extensively drug-resistant Pseudomonas aeruginosa | 10.1016/j.ijantimicag.2017.04.001 | NO | NO | LETTER |
| 2018 | M. Dirat, D. Mizab, P. Renaudin and M. Villiet | Assessment of good use of new cephalosporins in a university hospital centre | 10.1007/s11096-017-0565-9 | NO | NO | REVIEW |
| 2017 | J. Dobias, V. Denervaud-Tendon, L. Poirel and P. Nordmann | Activity of the novel siderophore cephalosporin cefiderocol against multidrug-resistant Gram-negative pathogens | 10.1007/s10096-017-3063-z | NO | NO | IN VITRO STUDY |
| 2019 | C. Doern | Who needs susceptibility testing? Know your mechanisms of resistance | 10.1016/j.clinmicnews.2019.06.003 | NO | NO | OUT OF TOPIC |
| 2019 | Y. Doi | Treatment Options for Carbapenem-resistant Gram-negative Bacterial Infections | 10.1093/cid/ciz830 | NO | NO | REVIEW |
| 2015 | Y. Doi and D. L. Paterson | Carbapenemase-producing Enterobacteriaceae | 10.1055/s-0035-1544208 | NO | NO | REVIEW |
| 2019 | D. Dolce, N. Ravenni, S. Campana, E. Camera, C. Braggion and G. Taccetti | Ceftazidime-Avibactam and Ceftolozane/Tazobactam: New antimicrobials against multidrug-resistant Pseudomonas aeruginosa in Cystic Fibrosis | 10.1186/s13052-019-0631-0 | NO | NO | CONFERENCE PROCEEDINGS |
| 2018 | R. Domalaon, T. Idowu, G. G. Zhanel and F. Schweizer | Antibiotic hybrids: The next generation of agents and adjuvants against gram-negative pathogens? | 10.1128/CMR.00077-17 | NO | NO | REVIEW |
| 2010 | S. Donadio, S. Maffioli, P. Monciardini, M. Sosio and D. Jabes | Antibiotic discovery in the twenty-first century: Current trends and future perspectives | 10.1038/ja.2010.62 | NO | NO | REVIEW |
| 2015 | P. Doshi | Speeding new antibiotics to market: A fake fix? | 10.1136/bmj.h1453 | NO | NO | EDITORIAL |
| 2015 | R. Draenert, U. Seybold, E. Grützner and J. R. Bogner | Novel antibiotics: Are we still in the pre–post-antibiotic era? | 10.1007/s15010-015-0749-y | NO | NO | REVIEW |
| 2018 | D. F. Driscoll | Potentially important therapeutic interactions between antibiotics, and a specially engineered emulsion drug vehicle containing krill-oil-based phospholipids and omega-3 fatty acids | 10.3390/antibiotics7010022 | YES | NO | PHARMACOLOGICAL STUDY |
| 2016 | G. L. Drusano, W. Hope, A. MacGowan and A. Louie | Suppression of emergence of resistance in pathogenic bacteria: Keeping our powder dry, part 2 | 10.1128/AAC.02231-15 | NO | NO | REVIEW |
| 2014 | M. S. Dryden | Novel antibiotic treatment for skin and soft tissue infection | 10.1097/QCO.0000000000000050 | NO | NO | REVIEW |
| 2016 | L. R. Duncan, H. S. Sader, R. K. Flamm and M. Castanheira | Ceftolozane/tazobactam activity tested against bacterial bloodstream isolates from multiple infection sources | 10.1093/ofid/ofw172.1372 | NO | NO | IN VITRO STUDY |
| 2016 | C. R. Durand, M. Alsharhan and K. C. Willett | New and emerging antibiotics for complicated intra-abdominal infections | 10.1097/MJT.0000000000000433 | NO | NO | REVIEW |
| 2016 | C. Eckmann, K. Huber and B. Crabein | Ceftolozane/Tazobactam - A new option for the treatment of infections due to multiresistant gram-negative organisms |  | NO | NO | REVIEW |
| 2018 | C. Eckmann, M. Kaffarnik, M. Schappacher, R. Otchwemah and B. Grabein | [Multidrug resistant gram-negative bacteria : Clinical management pathway for patients undergoing elective interventions in visceral surgery] | 10.1007/s00104-017-0476-2 | NO | NO | REVIEW |
| 2018 | C. Eckmann, L. J. Rojas and S. Lyon | Know your enemy: managing resistant Gram-negative infections | 10.2217/fmb-2018-0202 | YES | NO | CONFERENCE PROCEEDINGS |
| 2015 | C. Eckmann and J. Solomkin | Ceftolozane/tazobactam for the treatment of complicated intra-abdominal infections | 10.1517/14656566.2015.994504 | NO | NO | REVIEW |
| 2018 | G. Edwards, M. Smith, A. Downes, M. Cullen, R. J. Bright-Thomas, A. M. Jones and P. J. Barry | In vitro susceptibility to ceftolozane/tazobactam in Pseudomonas aeruginosa isolates at an adult cystic fibrosis centre |  | NO | NO | IN VITRO STUDY |
| 2015 | J. K. Edwards | Infection: Ceftolozane-tazobactam gives a new ASPECT to the fight against antimicrobial resistance | 10.1038/nrurol.2015.103 | NO | NO | COMMENT |
| 2015 | J. K. Edwards | Urinary tract infection: Ceftolozane-tazobactam gives a new ASPECT to the fight against antimicrobial resistance | 10.1038/nrneph.2015.77 | NO | NO | COMMENT |
| 2020 | M. B. Ekkelenkamp, R. Canton, M. Diez-Aguilar, M. M. Tunney, D. F. Gilpin, F. Bernardini, G. E. Dale, J. S. Elborn, J. R. Bayjanov and A. Fluit | Susceptibility of Pseudomonas aeruginosa Recovered from Cystic Fibrosis Patients to Murepavadin and 13 Comparator Antibiotics | 10.1128/aac.01541-19 | NO | NO | IN VITRO STUDY |
| 2018 | N. G. El Chakhtoura, E. Saade, A. Iovleva, M. Yasmin, B. Wilson, F. Perez and R. A. Bonomo | Therapies for multidrug resistant and extensively drug-resistant non-fermenting gram-negative bacteria causing nosocomial infections: a perilous journey toward ‘molecularly targeted’ therapy | 10.1080/14787210.2018.1425139 | NO | NO | REVIEW |
| 2018 | A. Elabor, E. Molnar, M. King and J. Gallagher | Ceftolozane/tazobactam for the treatment of multidrug-resistant pseudomonas aeruginosa infections in immunocompromised patients: A multi-center study | 10.1093/ofid/ofy210.2035 | YES | NO | ABSTRACT |
| 2019 | S. Elizabeth Davis, J. Ham, J. Hucks, A. Gould, R. Foster, J. Ann Justo, D. P. Nicolau and P. B. Bookstaver | Use of continuous infusion ceftolozane-tazobactam with therapeutic drug monitoring in a patient with cystic fibrosis | 10.1093/ajhp/zxz011 | NO | NO | CASE REPORT |
| 2019 | D. El-Sayed and A. Nouvong | Infection Protocols for Implants | 10.1016/j.cpm.2019.06.007 | NO | NO | REVIEW |
| 2019 | C. Emeraud, L. Escaut, A. Boucly, N. Fortineau, R. A. Bonnin, T. Naas and L. Dortet | Aztreonam plus clavulanate, tazobactam, or avibactam for treatment of infections caused by metallo-lactamase-producing gram-negative bacteria | 10.1128/AAC.00010-19 | NO | NO | CASE REPORT |
| 2019 | L. Escaut, G. Cuzon, N. Fortineau, C. Humbert, I. Bukreyeva, N. Anguel, E. Lefevre, L. Dortet, T. Naas and A. Barrail-Tran | Prescription de ceftolozane/tazobactam et ceftazidime/avibactam dans un CHU | 10.1016/j.medmal.2019.04.150 | YES | NO | NOT IN ENGLISH |
| 2019 | L. Escola-Verge, C. Pigrau and B. Almirante | Ceftolozane/tazobactam for the treatment of complicated intra-abdominal and urinary tract infections: current perspectives and place in therapy | 10.2147/idr.S180905 | NO | NO | REVIEW |
| 2014 | M. Estabrook, B. Bussell, S. L. Clugston and K. Bush | In vitro activity of ceftolozane-tazobactam as determined by broth dilution and agar diffusion assays against recent U.S. Escherichia coli isolates from 2010 to 2011 carrying CTX-M-type extended-Spectrum β-lactamases | 10.1128/JCM.02357-14 | NO | NO | IN VITRO STUDY |
| 2020 | S. Etemadi, H. Ebrahimzadeh Leylabadlo and R. Ghotaslou | AmpC β-lactamase among Enterobacteriaceae: A new insight | 10.1016/j.genrep.2020.100673 | NO | NO | REVIEW |
| 2017 | E. S. Euctr | Ceftolozane/tazobactam (MK-7625A) plus Metronidazole versus Meropenem in pediatric complicated intra-abdominal infection |  | YES | NO | PROTOCOL |
| 2018 | F. R. Euctr | Comparison of short infusion versus prolonged infusion of ceftolozane-tazobactam among patients with ventilator associated-pneumonia to Pseudomonas aeruginosa in intensive care units |  | YES | NO | PHARMACOLOGICAL STUDY |
| 2017 | P. L. Euctr | Ceftolozane/tazobactam (MK-7625A) versus Meropenem in Pediatric Complicated Urinary Tract Infection |  | YES | NO | PROTOCOL |
| 2016 | S. Evans, T. T. T. Tran, A. Hujer, C. Hill, K. M. Hujer, J. R. Mediavilla, C. Manca, T. N. Domitrovic, B. N. Kreiswirth, R. Patel, M. R. Jacobs, F. Perez, L. Chen, R. Sampath, T. Hall, C. Marzan, V. Fowler, H. Chambers and R. A. Bonomo | Choosing ceftazidime/avibactam and ceftolozane/tazobactam as empiric therapies against pseudomonas aeruginosa (PA) using rapid molecular diagnostics (RMDS): Primers iv | 10.1093/ofid/ofw172.1598 | NO | NO | IN VITRO STUDY |
| 2019 | S. R. Evans, T. T. T. Tran, A. M. Hujer, C. B. Hill, K. M. Hujer, J. R. Mediavilla, C. Manca, T. N. Domitrovic, F. Perez, M. Farmer, K. M. Pitzer, B. M. Wilson, B. N. Kreiswirth, R. Patel, M. R. Jacobs, L. Chen, V. G. Fowler, H. F. Chambers and R. A. Bonomo | Rapid Molecular Diagnostics to Inform Empiric Use of Ceftazidime/Avibactam and Ceftolozane/Tazobactam Against Pseudomonas aeruginosa: PRIMERS IV | 10.1093/cid/ciy801 | NO | NO | IN VITRO STUDY |
| 2020 | C. Ezquer, J. Colomina, M. Luisa Blascoa, N. Carbonell, G. Aguilar, R. Ferriols, R. Huerta, J. Ferreres, M. Calabuig and M. Juan | Ceftolozane pharmacokinetics in a septic critically ill patient under different extracorporeal replacement therapies | 10.1128/AAC.01782-19 | NO | NO | CASE REPORT |
| 2018 | C. Ezquer-Garin, R. Ferriols-Lisart, M. Alos-Alminana, G. Aguilar-Aguilar, J. F. Belda-Nacher and J. A. Carbonell | Validated HPLC-UV detection method for the simultaneous determination of ceftolozane and tazobactam in human plasma | 10.4155/bio-2017-0257 | NO | NO | OUT OF TOPIC |
| 2017 | M. Falagas, T. Skalidis, K. Vardakas, N. Legakis, S. Tsiplakou, V. Papaioannou, E. Trikka-Graphakos, N. Charalampakis, C. Sereti, E. Lebessi, E. Bozavoutoglou, E. Vogiatzakis, H. Moraiti, A. Xanthaki, M. Toutouza, K. Fountoulis, E. Perivolioti, H. Kraniotaki, M. Bournia, S. Vourli, E. Peteinaki, S. Chatzipanagiotou, A. Ioannidis, A. Makri, M. Daskalaki, E. Staikou, K. Tzannetou, H. Prifti, K. Mentessidou, A. Maltezou, E. Platsouka, N. Skarmoutsou, I. Spiliopoulou, M. Christofidou, M. Falagas, K. Vardakas, N. Triarides, N. Legakis, N. Legakis, T. Skalidis, M. Falagas and M. Kyriakidou | Activity of cefiderocol (S-649266) against carbapenem-resistant Gram-negative bacteria collected from inpatients in Greek hospitals | 10.1093/jac/dkx049 | NO | NO | IN VITRO STUDY |
| 2018 | I. Falces-Romero, E. Cendejas-Bueno, M. Laplaza-González, L. Escosa-García, C. Schuffelmann-Gutiérrez, B. Calderón-Llopis, J. Pemán, P. de la Oliva and J. García-Rodríguez | T2Candida® to guide antifungal and lengh of treatment of candidemia in a pediatric multivisceral transplant recipient | 10.1016/j.mmcr.2018.05.006 | NO | NO | CASE REPORT |
| 2016 | V. Falco, J. Burgos, E. Papiol, R. Ferrer and B. Almirante | Investigational drugs in phase I and phase II clincial trials for the treatment of hospital-acquired pneumonia | 10.1517/13543784.2016.1168803 | NO | NO | REVIEW |
| 2016 | M. Falcone and D. Paterson | Spotlight on ceftazidime/avibactam: A new option for MDR Gram-negative infections | 10.1093/jac/dkw239 | NO | NO | REVIEW |
| 2017 | R. Fanci, P. Pecile, E. Riccobono, G. M. Rossolini and A. Bosi | Multidrug-resistant gram negative bacteremia in hematologic patients: Incidence and infection control practices in a monocentric experience |  | NO | NO | REVIEW |
| 2019 | L. Farooq, Z. Memon, M. O. Ismail and S. Sadiq | Frequency and antibiogram of multi-drug resistant pseudomonas aeruginosa in a tertiary care hospital of pakistan | 10.12669/pjms.35.6.930 | NO | NO | IN VITRO STUDY |
| 2013 | D. J. Farrell, R. K. Flamm, H. S. Sader and R. N. Jones | Antimicrobial activity of ceftolozane-tazobactam tested against Enterobacteriaceae and Pseudomonas aeruginosa with various resistance patterns isolated in U.S. hospitals (2011-2012) | 10.1128/AAC.01802-13 | NO | NO | IN VITRO STUDY |
| 2014 | D. J. Farrell, H. S. Sader, R. K. Flamm and R. N. Jones | Ceftolozane/tazobactam activity tested against Gram-negative bacterial isolates from hospitalised patients with pneumonia in US and European medical centres (2012) | 10.1016/j.ijantimicag.2014.01.032 | NO | NO | IN VITRO STUDY |
| 2019 | C. Faure, H. Pluchart, L. Quarteroni, S. Quetant, J. Claustre, S. Chanoine and P. Bedouch | Multidrug resistant Pseudomonas aeruginosa infection in a lung transplant recipient with cystic fibrosis: Which doses for ceftazidime/avibactam? | 10.1007/s11096-018-0759-9 | NO | NO | IN VITRO STUDY |
| 2017 | P. Fernandes and E. Martens | Antibiotics in late clinical development | 10.1016/j.bcp.2016.09.025 | NO | NO | REVIEW |
| 2020 | M. Fernandez-Esgueva, A. I. Lopez-Calleja, X. Mulet, P. A. Fraile-Ribot, G. Cabot, R. Huarte, A. Rezusta and A. Oliver | Characterization of AmpC beta-lactamase mutations of extensively drug-resistant Pseudomonas aeruginosa isolates that develop resistance to ceftolozane/tazobactam during therapy | 10.1016/j.eimc.2020.01.017 | NO | NO | IN VITRO STUDY |
| 2018 | R. Ferriols Lisart, A. Pérez Pitarch, F. Ferriols Lisart and M. Alós Almiñana | An indirect comparison of efficacy and safety of ceftolozane/tazobactam, ceftazidime/avibactam versus meropenem in complicated intra-abdominal infections |  | NO | NO | REVIEW |
| 2018 | T. Ferry, F. Boucher, C. Fevre, T. Perpoint, J. Chateau, C. Petitjean, J. Josse, C. Chidiac, G. L’hostis, G. Leboucher and F. Laurent | Innovations for the treatment of a complex bone and joint infection due to XDR Pseudomonas aeruginosa including local application of a selected cocktail of bacteriophages | 10.1093/jac/dky263 | NO | NO | CASE REPORT |
| 2015 | J. D. Finklea, R. Hollaway, K. Lowe, J. Ciurca, B. Le, F. Lee and R. Jain | Ceftolozane/tazobactam sensitivity patterns in pseudomonas aeuruginosa isolates from cystic fibrosis patients | 10.1002/ppul.23297 | NO | NO | IN VITRO STUDY |
| 2019 | M. Fiore, S. Di Franco, A. Alfieri, M. B. Passavanti, M. C. Pace, M. E. Kelly, G. Damiani and S. Leone | Spontaneous bacterial peritonitis caused by Gram-negative bacteria: an update of epidemiology and antimicrobial treatments | 10.1080/17474124.2019.1621167 | NO | NO | REVIEW |
| 2017 | L. K. Flynt, M. P. Veve, L. P. Samuel and R. J. Tibbetts | Comparison of etest to broth microdilution for testing of susceptibility of Pseudomonas aeruginosa to ceftolozane-tazobactam | 10.1128/JCM.01920-16 | NO | NO | IN VITRO STUDY |
| 2018 | L. K. Flynt, M. P. Veve, L. P. Samuel and R. J. Tibbetts | Retraction: Comparison of etest to broth microdilution for testing of susceptibility of pseudomonas aeruginosa to ceftolozane-Tazobactam (Journal of Clinical Microbiology (2018) 56 (e00541-18) DOI: 10.1128/JCM.00541-18) | 10.1128/JCM.00541-18 | NO | NO | LETTER |
| 2018 | J. B. Forrester, L. L. Steed, B. A. Santevecchi, P. Flume, G. E. Palmer-Long and J. A. Bosso | In vitro activity of ceftolozane/tazobactam vs nonfermenting, gram- negative cystic fibrosis isolates | 10.1093/ofid/ofy158 | NO | NO | IN VITRO STUDY |
| 2016 | C. Forstner and M. W. Pletz | Multidrug-resistant organisms - Treatment strategies | 10.1055/s-0041-103158 | NO | NO | REVIEW |
| 2017 | R. Foster, A. P. Gould, J. A. Justo, S. Okoye, D. P. Nicolau and P. B. Bookstaver | Pharmacokinetic assessment of continuous infusion ceftolozane/ tazobactam for drug-resistant pseudomonas aeruginosa left ventricular assist device driveline infection | 10.1093/ofid/ofx163.635 | NO | NO | IN VITRO STUDY |
| 2018 | P. A. Fraile-Ribot, G. Cabot, X. Mulet, L. Periañez, M. Luisa Martín-Pena, C. Juan, J. L. Pérez and A. Oliver | Mechanisms leading to in vivo ceftolozane/tazobactam resistance development during the treatment of infections caused by MDR Pseudomonas aeruginosa | 10.1093/jac/dkx424 | NO | NO | IN VITRO STUDY |
| 2019 | P. A. Fraile-Ribot, C. Del Rosario-Quintana, C. Lopez-Causape, M. A. Gomis-Font, M. Ojeda-Vargas and A. Oliver | Emergence of Resistance to Novel beta-Lactam-beta-Lactamase Inhibitor Combinations Due to Horizontally Acquired AmpC (FOX-4) in Pseudomonas aeruginosa Sequence Type 308 | 10.1128/aac.02112-19 | NO | NO | LETTER |
| 2017 | P. A. Fraile-Ribot, X. Mulet, G. Cabot, E. Del Barrio-Tofino, C. Juan, J. L. Perez and A. Oliver | In Vivo Emergence of Resistance to Novel Cephalosporin-beta-Lactamase Inhibitor Combinations through the Duplication of Amino Acid D149 from OXA-2 beta-Lactamase (OXA-539) in Sequence Type 235 Pseudomonas aeruginosa | 10.1128/aac.01117-17 | NO | NO | IN VITRO STUDY |
| 2020 | P. A. Fraile-Ribot, L. Zamorano, R. Orellana, E. Del Barrio-Tofino, I. Sanchez-Diener, S. Cortes-Lara, C. Lopez-Causape, G. Cabot, G. Bou, L. Martinez-Martinez and A. Oliver | Activity of Imipenem-Relebactam against a Large Collection of Pseudomonas aeruginosa Clinical Isolates and Isogenic beta-Lactam-Resistant Mutants | 10.1128/aac.02165-19 | NO | NO | IN VITRO STUDY |
| 2019 | B. T. Franssens, A. C. Fluit and R. J. Rentenaar | Reproducibility between two readout methods of a commercial broth microdilution assay for Pseudomonas aeruginosa isolates from patients with Cystic Fibrosis | 10.1080/23744235.2018.1500705 | NO | NO | IN VITRO STUDY |
| 2018 | A. Frattari, V. Savini, E. Polilli, D. Cibelli, S. Talamazzi, D. Bosco, A. Consorte, P. Fazii and G. Parruti | Ceftolozane-tazobactam and Fosfomycin for rescue treatment of otogenous meningitis caused by XDR Pseudomonas aeruginosa: Case report and review of the literature | 10.1016/j.idcr.2018.e00451 | NO | NO | CASE REPORT |
| 2017 | T. Freeman, G. Eschenauer, T. Patel, T. Gandhi, L. Petty, C. Chenoweth, M. Santarossa and J. Nagel | Evaluating the need for antibiotic stewardship prospective audit and feedback on weekends | 10.1017/ice.2017.174 | NO | NO | OUT OF TOPIC |
| 2019 | M. Gajdács and F. Albericio | Antibiotic resistance: from the bench to patients | 10.3390/antibiotics8030129 | NO | NO | EDITORIAL |
| 2019 | L. Gambitta, E. Galfrascoli, E. Calzavara, V. Curci and M. L. A. Medaglia | From the surveillance of antibiotics prescriptions to limitation of multidrug resistant bacteria strains: The role of the hospital pharmacy in defining a carbapenem-sparing-strategy | 10.1136/ejhpharm-2019-eahpconf.201 | NO | NO | OUT OF TOPIC |
| 2015 | L. M. Gangcuangco, P. Clark, C. Stewart, G. Miljkovic and Z. Saul | Persistent bacteremia from Pseudomonas aeruginosa resistant to the novel antibiotics ceftolozane-tazobactam and ceftazidime-avibactam | 10.1111/tmi.12574 | NO | NO | CASE REPORT |
| 2016 | L. M. Gangcuangco, P. Clark, C. Stewart, G. Miljkovic and Z. K. Saul | Persistent Bacteremia from Pseudomonas aeruginosa with In Vitro Resistance to the Novel Antibiotics Ceftolozane-Tazobactam and Ceftazidime-Avibactam | 10.1155/2016/1520404 | NO | NO | IN VITRO STUDY |
| 2018 | Y. Gao and C. Shu | Progress in researches for ceftolozane/tazobactam and ceftazidime/ avibactam against Pseudomonas aeruginosa |  | NO | NO | REVIEW |
| 2013 | J. Garau | New antibiotics | 10.1016/S0924-8579(13)70207-3 | NO | NO | CONFERENCE PROCEEDINGS |
| 2019 | M. Garcia, B. Feal-Cortizas, C. Fernandez-Oliveira, F. Lamelo-Alfonsin and I. Martin-Herranz | Avoided costs using elastomeric infusion pumps in hospital at home to administrate parenteral antibiotics | 10.1111/ijcp.13328 | NO | NO | OUT OF TOPIC |
| 2019 | M. García-castillo, S. García-Fernández, J. Melo Cristino, C. Chaves, H. Ramos, M. Ribeiro, M. Feijó Pinto, J. Diogo, E. Gonçalves, L. Sancho, R. Ferreira, V. Alves, E. Ramalheira, J. Romano, L. Pássaro and R. Cantón | Ceftolozane-tazobactam in vitro activity in Pseudomonas aeruginosa and enterobacterales isolates recovered in intensive care units in Portugal. STEP study | 10.1186/s13054-019-2358-0 | NO | NO | IN VITRO STUDY |
| 2019 | S. Garcia-Fernandez, M. Garcia-Castillo, G. Bou, J. Calvo, E. Cercenado, M. Delgado, C. Pitart, X. Mulet, N. Tormo, D. L. Mendoza, J. Diaz-Reganon and R. Canton | Activity of ceftolozane/tazobactam against Pseudomonas aeruginosa and Enterobacterales isolates recovered from intensive care unit patients in Spain: The SUPERIOR multicentre study | 10.1016/j.ijantimicag.2019.02.004 | NO | NO | IN VITRO STUDY |
| 2020 | S. Garcia-Fernandez, M. Garcia-Castillo, J. Melo-Cristino, M. F. Pinto, E. Goncalves, V. Alves, A. R. Vieira, E. Ramalheira, L. Sancho, J. Diogo, R. Ferreira, D. Silva, C. Chaves, L. Passaro, L. Paixao and R. Canton | In vitro activity of ceftolozane-tazobactam against Enterobacterales and Pseudomonas aeruginosa causing urinary, intra-abdominal and lower respiratory tract infections in intensive care units in Portugal: The STEP multicenter study | 10.1016/j.ijantimicag.2020.105887 | NO | NO | IN VITRO STUDY |
| 2015 | A. García-Robles, V. Bosó-Ribelles, E. Romá-Sánchez and J. L. Poveda-Andrés | New chemotherapeutic agents against common infections: Clinical studies on new anti-infective agents | 10.2174/9781681080826115040007 | NO | NO | BOOK |
| 2018 | M. Garg, J. Talmud and Z. Repanshek | Commentary | 10.1016/j.annemergmed.2018.05.012 | NO | NO | COMMENT |
| 2010 | Y. Ge, M. J. Whitehouse, I. Friedland and G. H. Talbot | Pharmacokinetics and safety of CXA-101, a new antipseudomonal cephalosporin, in healthy adult male and female subjects receiving single- and multiple-dose intravenous infusions | 10.1128/AAC.01753-09 | NO | NO | PHARMACOLOGICAL STUDY |
| 2015 | M. S. Gelfand and K. O. Cleveland | Ceftolozane/tazobactam therapy of respiratory infections due to multidrug-resistant pseudomonas aeruginosa | 10.1093/cid/civ411 | NO | NO | LETTER |
| 2016 | S. Gelot and E. Nakhla | Intra-abdominal infections in adults |  | NO | NO | REVIEW |
| 2017 | I. Gentile, A. R. Buonomo, A. E. Maraolo, R. Scotto, F. De Zottis, G. Di Renzo and G. Borgia | Successful treatment of post-surgical osteomyelitis caused by XDR pseudomonas aeruginosa with ceftolozane/tazobactam monotherapy | 10.1093/jac/dkx172 | NO | NO | CASE REPORT |
| 2016 | I. Gentile, A. E. Maraolo and G. Borgia | What is the role of the new β-lactam/β-lactamase inhibitors ceftolozane/tazobactam and ceftazidime/avibactam? | 10.1080/14787210.2016.1233060 | NO | NO | EDITORIAL |
| 2017 | P. Geraldy, R. Krause and I. Zollner-Schwetz | Susceptibility of carbapenem-resistant Pseudomonas aeruginosa clinical isolates to novel cephalosporine antibiotics | 10.1007/s00508-017-1246-3 | NO | NO | IN VITRO STUDY |
| 2018 | A. Gerlach and J. Bazan | Ceftolozane/tazobactam for treatment of osteomyelitis due to multi-drug-resistant pseudomonas aeruginosa | 10.1093/ofid/ofy210.303 | YES | NO | ABSTRACT |
| 2016 | C. N. Geyer, R. C. Fowler, J. R. Johnson, B. Johnston, S. J. Weissman, P. Hawkey and N. D. Hanson | Evaluation of CTX-M steady-state mRNA, mRNA half-life and protein production in various STs of Escherichia coli | 10.1093/jac/dkv388 | NO | NO | IN VITRO STUDY |
| 2019 | G. Gherardi, G. Linardos, A. Pompilio, E. Fiscarelli and G. Di Bonaventura | Evaluation of in vitro activity of ceftolozane-tazobactam compared to other antimicrobial agents against Pseudomonas aeruginosa isolates from cystic fibrosis patients | 10.1016/j.diagmicrobio.2019.01.012 | NO | NO | IN VITRO STUDY |
| 2018 | D. R. Giacobbe, M. Bassetti, F. G. De Rosa, V. Del Bono, P. A. Grossi, F. Menichetti, F. Pea, G. M. Rossolini, M. Tumbarello, P. Viale and C. Viscoli | Ceftolozane/tazobactam: place in therapy | 10.1080/14787210.2018.1447381 | NO | NO | REVIEW |
| 2018 | D. R. Giacobbe, M. Mikulska and C. Viscoli | Recent advances in the pharmacological management of infections due to multidrug-resistant Gram-negative bacteria | 10.1080/17512433.2018.1549487 | NO | NO | REVIEW |
| 2020 | D. R. Giacobbe, C. Saffioti, A. R. Losito, M. Rinaldi, C. Aurilio, C. Bolla, S. Boni, G. Borgia, N. Carannante, G. Cassola, G. Ceccarelli, S. Corcione, D. Dalla Gasperina, F. G. De Rosa, C. Dentone, S. Di Bella, N. Di Lauria, M. Feasi, M. Fiore, S. Fossati, E. Franceschini, A. Gori, G. Granata, S. Grignolo, P. A. Grossi, G. Guadagnino, F. Lagi, A. E. Maraolo, V. Marinò, M. Mazzitelli, A. Mularoni, A. Oliva, M. C. Pace, A. Parisini, F. Patti, N. Petrosillo, V. Pota, F. Raffaelli, M. Rossi, A. Santoro, C. Tascini, C. Torti, E. M. Trecarichi, M. Venditti, P. Viale, A. Signori, M. Bassetti, V. Del Bono, M. Giannella, M. Mikulska, M. Tumbarello and C. Viscoli | Use of colistin in adult patients: A cross-sectional study | 10.1016/j.jgar.2019.06.009 | YES | NO | NO C/T DATA |
| 2016 | S. E. Giancola, M. V. Mahoney, T. E. Bias and E. B. Hirsch | Critical evaluation of ceftolozane–tazobactam for complicated urinary tract and intra-abdominal infections | 10.2147/TCRM.S83844 | NO | NO | REVIEW |
| 2018 | T. Giani, F. Arena, S. Pollini, V. Di Pilato, M. M. D'Andrea, L. H. De Angelis, M. Bassetti, G. M. Rossolini, C. Vismara, F. Luzzaro, R. Cavallo, P. A. Dusi, E. Pagani, M. Sarti, C. Farina, R. Rigoli, C. Scarparo, W. P. Pecile, M. G. Cusi, A. Mencacci, E. Manso, T. Spanu, M. Labonia, V. Tassi, G. Amato, S. Stefani, C. Giraldi and M. Rassu | Italian nationwide survey on pseudomonas aeruginosa from invasive infections: Activity of ceftolozane/tazobactam and comparators, and molecular epidemiology of carbapenemase producers | 10.1093/jac/dkx453 | NO | NO | IN VITRO STUDY |
| 2020 | M. Giannella, M. Bartoletti, M. Gatti and P. Viale | Advances in the therapy of bacterial bloodstream infections | 10.1016/j.cmi.2019.11.001 | NO | NO | REVIEW |
| 2009 | C. G. Giske, J. Ge and P. Nordmann | Activity of cephalosporin CXA-101 (FR264205) and comparators against extended-spectrum-β-lactamase-producing Pseudomonas aeruginosa | 10.1093/jac/dkp193 | NO | NO | COMMENT |
| 2016 | D. A. Goff and T. M. File | The Evolving Role of Antimicrobial Stewardship in Management of Multidrug Resistant Infections | 10.1016/j.idc.2016.02.012 | NO | NO | REVIEW |
| 2015 | D. A. Goff and M. J. Rybak | Global Antimicrobial Stewardship: Challenges and Successes from Frontline Stewards | 10.1007/s40121-015-0088-4 | NO | NO | REVIEW |
| 2015 | Y. Golan | Empiric therapy for hospital-acquired, Gram-negative complicated intra-abdominal infection and complicated urinary tract infections: A systematic literature review of current and emerging treatment options | 10.1186/s12879-015-1054-1 | NO | NO | REVIEW |
| 2020 | A. R. Golden, H. J. Adam, M. Baxter, A. Walkty, P. Lagacé-Wiens, J. A. Karlowsky and G. G. Zhanel | In Vitro Activity of Cefiderocol, a Novel Siderophore Cephalosporin, against Gram-Negative Bacilli Isolated from Patients in Canadian Intensive Care Units | 10.1016/j.diagmicrobio.2020.115012 | NO | NO | IN VITRO STUDY |
| 2019 | J. Gomez-Junyent, E. Benavent, Y. Sierra, C. El Haj, L. Soldevila, B. Torrejon, R. Rigo-Bonnin, F. Tubau, J. Ariza and O. Murillo | Efficacy of ceftolozane/tazobactam, alone and in combination with colistin, against multidrug-resistant Pseudomonas aeruginosa in an in vitro biofilm pharmacodynamic model | 10.1016/j.ijantimicag.2019.01.010 | NO | NO | IN VITRO STUDY |
| 2019 | J. A. Gonzales Zamora and Y. Varadarajalu | Fatal Curvularia brain abscess in a heart and kidney transplant recipient | 10.1016/j.idcr.2019.e00576 | NO | NO | CASE REPORT |
| 2017 | M. D. Gonzalez, A. R. McMullen, M. A. Wallace, M. P. Crotty, D. J. Ritchie and C. A. Burnham | Susceptibility of Ceftolozane-Tazobactam and Ceftazidime-Avibactam Against a Collection of β-Lactam-Resistant Gram-Negative Bacteria | 10.3343/alm.2017.37.2.174 | NO | NO | LETTER |
| 2015 | M. D. Gonzalez, M. A. Wallace, T. Hink, E. R. Dubberke and C. A. Burnham | Ceftolozane-tazobactam activity against phylogenetically diverse Clostridium difficile strains | 10.1128/aac.01670-15 | NO | NO | IN VITRO STUDY |
| 2017 | V. J. González Ramallo, M. M. Rubio, O. E. Cuxart and M. E. G. Leoni | Usefulness of hospital at home in nosocomial infections: Advantages and limitations |  | NO | NO | REVIEW |
| 2016 | K. J. Goodlet, D. P. Nicolau and M. D. Nailor | Ceftolozane/tazobactam and ceftazidime/avibactam for the treatment of complicated intra-abdominal infections | 10.2147/TCRM.S120811 | NO | NO | REVIEW |
| 2017 | K. J. Goodlet, D. P. Nicolau and M. D. Nailor | In vitro comparison of ceftolozane-tazobactam to traditional beta-lactams and ceftolozane-tazobactam as an alternative to combination antimicrobial therapy for Pseudomonas aeruginosa | 10.1128/AAC.01350-17 | NO | NO | IN VITRO STUDY |
| 2019 | I. M. Gould, C. Gunasekera and A. Khan | Antibacterials in the pipeline and perspectives for the near future | 10.1016/j.coph.2019.05.001 | NO | NO | REVIEW |
| 2017 | G. C. Graffius, B. M. Jocher, D. Zewge, H. M. Halsey, G. Lee, F. Bernardoni, X. Bu, R. Hartman and E. L. Regalado | Generic gas chromatography-flame ionization detection method for quantitation of volatile amines in pharmaceutical drugs and synthetic intermediates | 10.1016/j.chroma.2017.08.048 | NO | NO | OUT OF TOPIC |
| 2016 | A. Gramegna, B. Millar, F. Blasi, J. S. Elborn, D. Downey and J. E. Moore | Antimicrobial susceptibility of ceftabiprole and ceftolozane/tazobactam against CF and non-CF P. Aeruginosa | 10.1002/ppul.23576 | NO | NO | IN VITRO STUDY |
| 2018 | A. Gramegna, B. C. Millar, F. Blasi, J. S. Elborn, D. G. Downey and J. E. Moore | In vitro antimicrobial activity of ceftolozane/tazobactam against Pseudomonas aeruginosa and other non-fermenting Gram-negative bacteria in adults with cystic fibrosis | 10.1016/j.jgar.2018.03.002 | NO | NO | IN VITRO STUDY |
| 2017 | P. Grohs, G. Taieb, P. Morand, I. Kaibi, I. Podglajen, M. Lavollay, J. L. Mainardi and F. Compain | In vitro activity of ceftolozane-tazobactam against multidrug-resistant nonfermenting Gram-negative bacilli isolated from patients with cystic fibrosis | 10.1128/AAC.02688-16 | NO | NO | IN VITRO STUDY |
| 2017 | M. Grupper, J. L. Kuti and D. P. Nicolau | In vitro blood culture bottle inoculation of whole blood with clinically relevant antibiotic concentrations: a word of caution | 10.1007/s10096-016-2874-7 | NO | NO | IN VITRO STUDY |
| 2017 | M. Grupper, D. P. Nicolau, J. Aslanzadeh, L. K. Tanner and J. L. Kuti | Effects of clinically meaningful concentrations of antipseudomonal β-lactams on time to detection and organism growth in blood culture bottles | 10.1128/JCM.01241-17 | NO | NO | IN VITRO STUDY |
| 2017 | M. Grupper, D. P. Nicolau, L. Tanner and J. L. Kuti | The effect of clinical concentrations of meropenem (MEM), Ceftolozane/ tazobactam (C/T), and Ceftazidime/avaibactam (CZA) on Time to Detection (TTD) and Growth of Pseudomonas aeruginosa (PSA) in bioMerieux BacT/ ALERT®FA Plus Blood Culture (BC) Bottles | 10.1093/ofid/ofx163.1571 | NO | NO | IN VITRO STUDY |
| 2016 | M. Grupper, C. Sutherland and D. Nicolau | Ceftolozane/tazobactam and ceftazidime/ avibactam against meropenem-resistant pseudomonas aeruginosa | 10.1097/01.ccm.0000509328.02782.df | NO | NO | ABSTRACT |
| 2017 | M. Grupper, C. Sutherland and D. P. Nicolau | Multicenter evaluation of ceftazidime-avibactam and ceftolozane-tazobactam inhibitory activity against meropenem-nonsusceptible Pseudomonas aeruginosa from blood, respiratory tract, and wounds | 10.1128/AAC.00875-17 | NO | NO | IN VITRO STUDY |
| 2020 | C. Gudiol, A. Albasanz-Puig, J. Laporte-Amargós, N. Pallarès, A. Mussetti, I. Ruiz-Camps, P. Puerta-Alcalde, E. Abdala, C. Oltolini, M. Akova, M. Montejo, M. Mikulska, P. Martín-Dávila, F. Herrera, O. Gasch, L. Drgona, H. Paz Morales, A. S. Brunel, E. García, B. Isler, W. V. Kern, I. Morales, G. Maestro-De La Calle, M. Montero, S. S. Kanj, O. R. Sipahi, S. Calik, I. Márquez-Gómez, J. I. Marin, M. Z. R. Gomes, P. Hemmatti, R. Araos, M. Peghin, J. L. Del Pozo, L. Yáñez, R. Tilley, A. Manzur, A. Novo, J. Carratalà, G. Cuervo, F. Escrihuela-Vidal, F. Tubau, C. Tebé, M. R. Arias, J. Aguilar-Company, N. Larrosa, C. Cardozo, C. Garcia-Vidal, I. Karim-Yaqub, R. Greco, P. Cichero, C. M. Ayaz, R. Céspedes, L. López-Soria, L. Magnasco, J. Fortún, D. Torres, A. Boté, M. Espasa, M. H. Montaguti, P. Y. Bochud, O. Manuel, S. T. Carrasco, J. S. López, H. Bertz, S. Rieg, M. De Cueto, J. Rodríguez-Baño, M. Lizasoain, J. M. Aguado, J. P. Horcajada, S. El Zein, J. F. Jabbour, A. Uyan-Onal, A. Nazli-Zeka, B. Palop, L. C. Correa, A. Aparecida Da Silva Machado, J. P. S. Tonhá, G. Maschmeyer, J. Munita, M. Bassetti, N. Castaldo and P. Sangro Del Alcázar | Clinical predictive model of multidrug resistance in neutropenic cancer patients with bloodstream infection due to Pseudomonas aeruginosa | 10.1128/AAC.02494-19 | YES | NO | OUT OF TOPIC |
| 2019 | C. Gudiol, G. Cuervo and J. Carratala | Optimizing therapy of bloodstream infection due to extended-spectrum beta-lactamase-producing Enterobacteriaceae | 10.1097/mcc.0000000000000646 | NO | NO | REVIEW |
| 2019 | K. Gupta and S. Dash | Efficacy of newer β lactams/ β lactamase inhibitors for treatment of multidrug resistant gram negative infections in burn patients | 10.1016/j.burns.2019.07.044 | NO | NO | LETTER |
| 2018 | G. Gustinetti, G. Cangemi, R. Bandettini and E. Castagnola | Pharmacokinetic/pharmacodynamic parameters for treatment optimization of infection due to antibiotic resistant bacteria: a summary for practical purposes in children and adults | 10.1080/1120009X.2017.1377909 | NO | NO | PHARMACOLOGICAL STUDY |
| 2016 | G. Gustinetti and M. Mikulska | Bloodstream infections in neutropenic cancer patients: A practical update | 10.1080/21505594.2016.1156821 | NO | NO | REVIEW |
| 2017 | T. Gustot, J. Fernandez, G. Szabo, A. Albillos, A. Louvet, R. Jalan, R. Moreau and C. Moreno | Sepsis in alcohol-related liver disease | 10.1016/j.jhep.2017.06.013 | NO | NO | REVIEW |
| 2016 | M. Hackel, M. Tsuji, R. Echols and D. Sahm | In vitro antibacterial activity of S-649266 against gram-negative clinical strains collected in North America and Europe, 2015 | 10.1093/ofid/ofw172.1376 | NO | NO | IN VITRO STUDY |
| 2018 | M. A. Hackel, M. Tsuji, Y. Yamano, R. Echols, J. A. Karlowsky and D. F. Sahm | In vitro activity of the siderophore cephalosporin, cefiderocol, against carbapenem-nonsusceptible and multidrug-resistant isolates of gram-negative bacilli collected worldwide in 2014 to 2016 | 10.1128/AAC.01968-17 | NO | NO | IN VITRO STUDY |
| 2017 | M. A. Hackel, M. Tsuji, Y. Yamano, R. Echols, J. A. Karlowsky and D. F. Sahma | In vitro activity of the siderophore cephalosporin, cefiderocol, against a recent collection of clinically relevant gram-negative Bacilli from North America and Europe, including carbapenem-nonsusceptible isolates (SIDERO-WT-2014 study) | 10.1128/AAC.00093-17 | NO | NO | IN VITRO STUDY |
| 2018 | J. B. Hagemann, N. Pfennigwerth, S. G. Gatermann, H. von Baum and A. Essig | KPC-2 carbapenemase-producing Pseudomonas aeruginosa reaching Germany | 10.1093/jac/dky105 | NO | NO | CASE REPORT |
| 2016 | A. W. Hahn, R. Jain and D. H. Spach | New Approaches to Antibiotic Use and Review of Recently Approved Antimicrobial Agents | 10.1016/j.mcna.2016.03.012 | NO | NO | REVIEW |
| 2018 | M. Hakki and J. S. Lewis | Ceftolozane-tazobactam therapy for multidrug-resistant Pseudomonas aeruginosa infections in patients with hematologic malignancies and hematopoietic-cell transplant recipients | 10.1007/s15010-018-1125-5 | NO | NO | CASE REPORT |
| 2020 | B. A. Hansen, Ø Wendelbo, Ø Bruserud, A. L. Hemsing, K. A. Mosevoll and H. Reikvam | Febrile neutropenia in acute leukemia. Epidemiology, etiology, pathophysiology and treatment | 10.4084/MJHID.2020.009 | NO | NO | REVIEW |
| 2020 | H. Harmanjeet, H. Jani, S. T. R. Zaidi, T. Wanandy, R. L. Castelino, K. Sud, G. M. Peterson and R. P. Patel | Stability of ceftolozane and tazobactam in different peritoneal dialysis solutions | 10.1177/0896860820902590 | NO | NO | OUT OF TOPIC |
| 2019 | D. E. Hart, J. C. Gallagher, L. A. Puzniak and E. B. Hirsch | Ceftolozane-Tazobactam (C/T) treatment outcomes in immunocompromised (IC) patients with multidrug-resistant (MDR) pseudomonas aeruginosa (PA) infections | 10.1093/ofid/ofz360.1959 | YES | NO | ABSTRACT |
| 2019 | R. Hartl, H. Kerschner, R. Gattringer, S. Lepuschitz, F. Allerberger, S. Sorschag, W. Ruppitsch and P. Apfalter | Whole-Genome Analysis of a Human Enterobacter mori Isolate Carrying a blaIMI-2 Carbapenemase in Austria | 10.1089/mdr.2018.0098 | NO | NO | CASE REPORT |
| 2017 | R. Hartl, H. Kerschner, S. Lepuschitz, W. Ruppitsch, F. Allerberger and P. Apfalter | Detection of the mcr-1 gene in a multidrug-resistant Escherichia coli isolate from an Austrian patient | 10.1128/AAC.02623-16 | NO | NO | CASE REPORT |
| 2018 | S. Hassan, M. D. Kahn, N. Saraiya and P. Nori | Treatment of a complex orthopaedic infection due to extensively drug-resistant Pseudomonas aeruginosa | 10.1136/bcr-2017-223202 | NO | NO | CASE REPORT |
| 2012 | J. Hatcher, R. Dhillon and B. S. Azadian | Antibiotic resistance mechanisms in the intensive care unit 1A02 2C03 3A13 | 10.1177/175114371201300407 | NO | NO | REVIEW |
| 2019 | W. Hatem Amer, S. Abdel Rahman Elshweikh and N. Mohammad Hablas | Comparative Study Between β-Lactam/β-Lactamase Inhibitors as Alternatives for Carbapenems in the Treatment of Extended-Spectrum β-Lactamase-Producing Enterobacteriaceae | 10.1097/IPC.0000000000000716 | NO | NO | IN VITRO STUDY |
| 2018 | P. M. Hawkey, R. E. Warren, D. M. Livermore, C. A. M. McNulty, D. A. Enoch, J. A. Otter and A. P. R. Wilson | Treatment of infections caused by multidrug-resistant gram-negative bacteria: Report of the British society for antimicrobial chemotherapy/healthcare infection society/british infection association joint working party | 10.1093/jac/dky027 | NO | NO | REVIEW |
| 2016 | B. He, C. Lu, G. Zheng, X. He, M. Wang, G. Chen, G. Zhang and A. Lu | Combination therapeutics in complex diseases | 10.1111/jcmm.12930 | NO | NO | REVIEW |
| 2019 | A. Hecker, M. Reichert, C. J. Reuß, T. Schmoch, J. G. Riedel, E. Schneck, W. Padberg, M. A. Weigand and M. Hecker | Intra-abdominal sepsis: new definitions and current clinical standards | 10.1007/s00423-019-01752-7 | NO | NO | REVIEW |
| 2020 | A. J. Heffernan, F. B. Sime, J. Sun, J. Lipman, A. Kumar, K. Andrews, D. Ellwood, K. Grimwood and J. Roberts | β-lactam antibiotic versus combined β-lactam antibiotics and single daily dosing regimens of aminoglycosides for treating serious infections: A meta-analysis | 10.1016/j.ijantimicag.2019.10.020 | NO | NO | META-ANALYSIS |
| 2015 | S. Heinzl | Complicated urinary tract infections: Ceftolozane/tazobactam is effective in the case of resistant germs |  | NO | NO | BOOK |
| 2018 | A. Henderson, E. Tan, K. L. McCarthy and D. L. Paterson | Activity of ceftolozane/tazobactam against a collection of Pseudomonas aeruginosa isolates from bloodstream infections in Australia | 10.1016/j.pathol.2018.08.009 | NO | NO | IN VITRO STUDY |
| 2013 | A. Hennessey and M. C. Scott | The GAIN Act: Facilitating antibiotic drug development |  | NO | NO | OUT OF TOPIC |
| 2019 | M. Henry, J. Puwula, J. John Schoen, L. Puzniak, J. Raddatz, T. Van Schooneveld and S. Bergman | Treatment of pseudomonas infection with beta-lactams including ceftolozane-tazobactamin solid organ transplant recipients | 10.1111/ajt.15406 | YES | NO | ABSTRACT |
| 2018 | M. Henry, J. Schoen, L. Puzniak, J. Raddatz, T. Van Schooneveld and S. J. Bergman | Ceftolozane-tazobactam use and outcomes at an academic transplant center | 10.1002/jac5.1059 | YES | NO | ABSTRACT |
| 2020 | F. Hernández, P. Lasalvia, J. Garzón, C. Castañeda-Cardona, C. López, C. Beltran, M. Rojas and D. Rosselli | Cost-effectiveness of ceftolozane/tazobactam for the treatment of complicated intraabdominal and urinary tract infections in Colombia | 10.22354/in.v24i1.821 | NO | NO | COST-EFFECTIVENESS ANALYSIS |
| 2019 | C. Hernández-Gómez, E. De La Cadena, M. F. Mojica, A. Correa, M. Perengüez, S. Gutiérrez, C. Pallares, C. López, M. M. R. Rojas and M. V. Villegas | Comparative in vitro activity of ceftolozane/tazobactam and comparator agents against enterobacteriaceae and pseudomonas aeruginosa clinical isolates in Colombia | 10.1093/ofid/ofz360.1445 | NO | NO | IN VITRO STUDY |
| 2017 | A. Hernandez-Tejedor, C. D. Merino-Vega, A. Martin-Vivas, R. Ruiz de Luna-Gonzalez, A. Delgado-Iribarren, A. Gaban-Diez, I. Temprano-Gomez, N. de la Calle-Pedrosa, A. I. Gonzalez-Jimenez and A. Algora-Weber | Successful treatment of multidrug-resistant Pseudomonas aeruginosa breakthrough bacteremia with ceftolozane/tazobactam | 10.1007/s15010-016-0944-5 | NO | NO | CASE REPORT |
| 2016 | T. Hesterkamp | Antibiotics clinical development and pipeline | 10.1007/82_2015_451 | NO | NO | REVIEW |
| 2018 | Y. Hidaka, N. Kishi, S. Matsumoto, T. Yoshinari and M. Mori | In vitro antimicrobial activity of ceftolozane/tazobactam against clinical isolates from Japan |  | NO | NO | IN VITRO STUDY |
| 2020 | N. T. Hillock, J. Karnon, J. Turnidge and T. L. Merlin | Estimating the utilisation of unregistered antimicrobials in Australia | 10.1016/j.idh.2019.12.001 | NO | NO | OUT OF TOPIC |
| 2018 | E. Hirsch, D. Hart, A. Piche, A. Cubillos, K. Beaulac, A. Bandali, J. Raddatz, K. Boeser, B. Dionne, L. Puzniak, M. V. Mahoney and E. Gancher | A multi-center evaluation of outcomes following treatment with ceftolozane-tazobactam | 10.1002/jac5.1059 | YES | NO | ABSTRACT |
| 2020 | E. B. Hirsch, H. V. Brigman, P. C. Zucchi, A. Chen, J. C. Anderson, G. M. Eliopoulos, N. Cheung, A. Gilbertsen, R. C. Hunter, C. L. Emery and T. E. Bias | Ceftolozane-tazobactam and ceftazidime-avibactam activity against beta-lactam-resistant Pseudomonas aeruginosa and extended-spectrum beta-lactamase-producing Enterobacterales clinical isolates from U.S. medical centers | 10.1016/j.jgar.2020.04.017 | NO | NO | IN VITRO STUDY |
| 2017 | E. B. Hirsch, P. Zucchi, N. Cheung, K. Krevolin, C. Emery and T. Bias | Activity of ceftolozane/tazobactam (C/T) and ceftazidime/avibactam (CZA) against extended-spectrum b-lactamase (ESBL)-producing enterobacteriaceae and multidrug-resistant (MDR) pseudomonas aeruginosa isolates | 10.1093/ofid/ofx163.927 | NO | NO | IN VITRO STUDY |
| 2019 | S. Ho, L. Nguyen, T. Trinh and C. MacDougall | Recognizing and Overcoming Resistance to New Beta-Lactam/Beta-Lactamase Inhibitor Combinations | 10.1007/s11908-019-0690-9 | NO | NO | REVIEW |
| 2013 | M. C. Hong, D. I. Hsu and M. Bounthavong | Ceftolozane/tazobactam: A novel antipseudomonal cephalosporin and β-lactamase-inhibitor combination | 10.2147/IDR.S36140 | NO | NO | REVIEW |
| 2019 | P. M. Honore, A. Mugisha, L. Barreto Gutierrez, S. Redant, K. Kaefer, A. Gallerani and D. De Bels | Optimizing ceftolozane-tazobactam dosage during continuous renal replacement therapy: Additional insights | 10.1186/s13054-019-2692-2 | NO | NO | LETTER |
| 2017 | P. M. Honore and H. D. Spapen | Eravacycline for treatment of complicated intra-abdominal infections: The fire is not ignited! | 10.21037/atm.2017.08.15 | NO | NO | EDITORIAL |
| 2018 | C. Hooper, S. Elsayed, A. M. Bombassaro, C. Bailey and L. Bondy | Successful treatment of chronic spinal osteomyelitis caused by multidrug resistant pseudomonas aeruginosa with ceftolozane-tazobactam and surgical intervention |  | NO | NO | CASE REPORT |
| 2019 | J. P. Horcajada, M. Montero, A. Oliver, L. Sorli, S. Luque, S. Gomez-Zorrilla, N. Benito and S. Grau | Epidemiology and Treatment of Multidrug-Resistant and Extensively Drug-Resistant Pseudomonas aeruginosa Infections | 10.1128/cmr.00031-19 | NO | NO | REVIEW |
| 2017 | S. C. Hsueh, Y. T. Huang, C. H. Liao, Y. J. Lee and P. R. Hsueh | In vitro activities of cefiderocol (S-649266), ceftazidimeavibactam, ceftolozane-tazobactam, and other comparative drugs against Pseudomonas aeruginosa, Acinetobacter baumannii and Stenotrophomonas maltophilia associated with bloodstream infections |  | NO | NO | IN VITRO STUDY |
| 2019 | S. C. Hsueh, Y. J. Lee, Y. T. Huang, C. H. Liao, M. Tsuji and P. R. Hsueh | In vitro activities of cefiderocol, ceftolozane/tazobactam, ceftazidime/avibactam and other comparative drugs against imipenem-resistant Pseudomonas aeruginosa and Acinetobacter baumannii, and Stenotrophomonas maltophilia, all associated with bloodstream infections in Taiwan | 10.1093/jac/dky425 | NO | NO | IN VITRO STUDY |
| 2017 | T. D. Huang, P. Bogaerts, C. Berhin, M. Hoebeke, C. Bauraing and Y. Glupczynski | Increasing proportion of carbapenemase-producing Enterobacteriaceae and emergence of a MCR-1 producer through a multicentric study among hospital-based and private laboratories in Belgium from September to November 2015 | 10.2807/1560-7917.ES.2017.22.19.30530 | NO | NO | IN VITRO STUDY |
| 2020 | W. Huang, J. El Hamouche, G. Wang, M. Smith, C. Yin, A. Dhand, N. Dimitrova and J. T. Fallon | Integrated genome-wide analysis of an isogenic pair of pseudomonas aeruginosa clinical isolates with differential antimicrobial resistance to ceftolozane/tazobactam, ceftazidime/avibactam, and piperacillin/tazobactam | 10.3390/ijms21031026 | NO | NO | IN VITRO STUDY |
| 2018 | A. Hujer, W. Long, R. Olsen, B. N. Kreiswirth, S. R. Evans, J. Musser and R. Bonomo | Predicting β-lactam resistance using whole genome sequencing (WGS) in Klebsiella pneumoniae: The challenge of β-lactam inhibitors | 10.1093/ofid/ofy209.166 | NO | NO | IN VITRO STUDY |
| 2018 | R. M. Humphries, J. A. Hindler, P. Magnano, A. Wong-Beringer, R. Tibbetts and S. A. Miller | Performance of ceftolozane-tazobactam etest, MIC test strips, and disk diffusion compared to reference broth microdilution for-Lactam-Resistant Pseudomonas aeruginosa isolates | 10.1128/JCM.01633-17 | NO | NO | IN VITRO STUDY |
| 2017 | R. M. Humphries, J. A. Hindler, A. Wong-Beringer and S. A. Miller | Activity of ceftolozane-tazobactam and ceftazidime-avibactam against beta-lactam-resistant Pseudomonas aeruginosa isolates | 10.1128/AAC.01858-17 | NO | NO | IN VITRO STUDY |
| 2018 | R. M. Humphries, S. Kircher, A. Ferrell, K. M. Krause, R. Malherbe, A. Hsiung and C. A. D. Burnham | The continued value of disk diffusion for assessing antimicrobial susceptibility in clinical laboratories: Report from the clinical and laboratory standards institute methods development and standardization working group | 10.1128/JCM.00437-18 | NO | NO | IN VITRO STUDY |
| 2019 | J. A. Huntington, B. Yu, L. Li, E. Jensen, C. Bruno and E. G. Rhee | Outcomes in patients with renal impairment from a phase 3 clinical trial for ceftolozane-tazobactam (C/T) treatment of nosocomial pneumonia (ASPECT-NP) | 10.1093/ofid/ofz360.1905 | YES | NO | ABSTRACT |
| 2015 | D. A. Hussar | New drugs 2015, part 3 | 10.1097/01.NURSE.0000471408.58270.66 | NO | NO | BOOK |
| 2020 | T. Idowu, G. G. Zhanel and F. Schweizer | A dimer, but not monomer, of tobramycin potentiates Ceftolozane against multidrug-resistant and extensively drug-resistant Pseudomonas aeruginosa and delays resistance development | 10.1128/AAC.02055-19 | NO | NO | IN VITRO STUDY |
| 2018 | C. Inserra, A. Zovi, L. Zampogna, V. Scalzi, M. Piacenza and G. M. Zerega | Monitoring the use of high cost antibiotics at Sacco Hospital | 10.1007/s11096-017-0565-9 | NO | NO | OUT OF TOPIC |
| 2016 | A. Iovleva, F. Perez, S. H. Marshall, A. M. Hujer, M. R. Jacobs, U. Stiefel, A. Ray and R. A. Bonomo | Ceftazidime/avibactam and ceftolozane/tazobactam in treatment of pulmonary infections by imipenem resistant pseudomonas aeruginosa | 10.1093/ofid/ofw172.1551 | YES | NO | IN VITRO STUDY |
| 2019 | A. Iregui, Z. Khan, D. Landman and J. M. Quale | Activity of imipenem-relebactam and ceftolozane-tazobactam against a contemporary collection of gram-negative bacteria from New York City | 10.1093/ofid/ofz360.763 | NO | NO | IN VITRO STUDY |
| 2019 | S. Irum, R. F. Potter, R. Kamran, Z. Mustafa, M. A. Wallace, C. A. D. Burnham, G. Dantas and S. Andleeb | Draft genome sequence of a blaNDM-1- And blaPME-1-harboring pseudomonas aeruginosa clinical isolate from Pakistan | 10.1128/MRA.00107-19 | NO | NO | IN VITRO STUDY |
| 2019 | L. Issakhanian and P. Behzadi | Antimicrobial agents and urinary tract infections | 10.2174/1381612825999190619130216 | NO | NO | REVIEW |
| 2020 | J. F. Jabbour, S. L. Sharara and S. S. Kanj | Treatment of multidrug-resistant Gram-negative skin and soft tissue infections | 10.1097/QCO.0000000000000635 | NO | NO | REVIEW |
| 2017 | D. M. Jacobs, M. C. Safir, D. Huang, F. Minhaj, A. Parker and G. G. Rao | Triple combination antibiotic therapy for carbapenemase-producing Klebsiella pneumoniae: A systematic review | 10.1186/s12941-017-0249-2 | NO | NO | SYSTEMATIC REVIEW |
| 2017 | C. Jacqueline, K. Howland and L. Chesnel | In vitro activity of ceftolozane/tazobactam in combination with other classes of antibacterial agents | 10.1016/j.jgar.2017.04.003 | NO | NO | IN VITRO STUDY |
| 2013 | C. Jacqueline, A. Roquilly, C. Desessard, D. Boutoille, A. Broquet, V. Le Mabecque, G. Amador, G. Potel, J. Caillon and K. Asehnoune | Efficacy of ceftolozane in a murine model of Pseudomonas aeruginosa acute pneumonia: In vivo antimicrobial activity and impact on host inflammatory response | 10.1093/jac/dks343 | NO | NO | PRECLINICAL STUDY |
| 2017 | S. János | Clinical aspects of severe infections caused by antibiotic-resistant Gram-negative bacteria : The Empire strikes back? | 10.1556/650.2017.30858 | NO | NO | REVIEW |
| 2018 | S. S. Jean, N. Y. Lee, H. J. Tang, M. C. Lu, W. C. Ko and P. R. Hsueh | Carbapenem-resistant enterobacteriaceae infections: Taiwan aspects | 10.3389/fmicb.2018.02888 | NO | NO | REVIEW |
| 2018 | S. S. Jean, M. C. Lu, Z. Y. Shi, S. H. Tseng, T. S. Wu, P. L. Lu, P. L. Shao, W. C. Ko, F. D. Wang and P. R. Hsueh | In vitro activity of ceftazidime–avibactam, ceftolozane–tazobactam, and other comparable agents against clinically important Gram-negative bacilli: Results from the 2017 surveillance of multicenter antimicrobial resistance in Taiwan (SMART) | 10.2147/IDR.S175679 | NO | NO | IN VITRO STUDY |
| 2019 | K. Jeannot, M. Danassie, P. Triponney, M. Bour, T. Gueudet, R. Beyrouthy, R. Bonnet and P. Plésiat | A novel IncQ plasmid carrying gene blaCTX-M-3 in Pseudomonas aeruginosa | 10.1093/jac/dky454 | NO | NO | LETTER |
| 2016 | P. Jog | ‘Rationale’ of antibiotic therapy–Think before you ink ! | 10.1007/s13312-016-0928-7 | NO | NO | EDITORIAL |
| 2018 | J. R. Johnson and T. A. Russo | Acute pyelonephritis in adults | 10.1056/NEJMcp1702758 | NO | NO | CASE REPORT |
| 2017 | B. D. Johnston, P. Thuras and J. R. Johnson | Activity of ceftolozane-tazobactam (C/T) against escherichia coli isolates from U.S. veterans in relation to co-resistance and sequence type 131 (ST131) H30/H30RX status | 10.1093/ofid/ofx163.921 | NO | NO | IN VITRO STUDY |
| 2016 | J. C. Jolliff, J. Ho, J. Joson, A. Heidari and R. Johnson | Treatment of Polymicrobial Osteomyelitis with Ceftolozane-Tazobactam: Case Report and Sensitivity Testing of Isolates | 10.1155/2016/1628932 | NO | NO | CASE REPORT |
| 2020 | B. M. Jones, K. Huelfer and C. M. Bland | Clinical and safety evaluation of continuously infused ceftolozane/ tazobactam in the outpatient setting | 10.1093/ofid/ofaa014 | YES | NO | CASE SERIES |
| 2017 | B. M. Jones, B. Smith and C. M. Bland | Use of Continuous-Infusion Ceftolozane/Tazobactam in a Multidrug-Resistant Pseudomonas aeruginosa Urinary Tract Infection in the Outpatient Setting | 10.1177/1060028017701938 | YES | NO | LETTER |
| 2018 | S. Jorgensen, T. D. Trinh, E. J. Zasowski, A. M. Lagnf, S. Bhatia, S. Simon, S. Estrada, J. Rosenberg, M. Steed, S. L. Davis and M. J. Rybak | Multidrug-resistant gram-negative infections treated with ceftolozane-tazobactam: Impact of delayed initiation | 10.1093/ofid/ofy210.2037 | YES | NO | ABSTRACT |
| 2019 | Jprn jRCTs | Study on pharmacokinetics of prophylactic antibiotics for surgical site infection by ESBL-producing Escherichia coli after lower gastrointestinal surgery |  | NO | NO | PHARMACOLOGICAL STUDY |
| 2019 | C. Juan | Optimizing the management of Pseudomonas aeruginosa infections with tools for the detection of resistance mechanisms and pharmacokinetic/pharmacodynamic (PK/PD) analysis | 10.1016/j.eimc.2019.09.002 | NO | NO | EDITORIAL |
| 2010 | C. Juan, L. Zamorano, J. L. Perez, Y. Ge and A. Oliver | Activity of a new antipseudomonal cephalosporin, CXA-101 (FR264205), against carbapenem-resistant and multidrug-resistant Pseudomonas aeruginosa clinical strains | 10.1128/aac.00834-09 | NO | NO | IN VITRO STUDY |
| 2019 | M. Kakara, K. Larson, H. P. Feng, M. Shiomi, H. Yoshitsugu and M. L. Rizk | Population pharmacokinetics of tazobactam/ceftolozane in Japanese patients with complicated urinary tract infection and complicated intra-abdominal infection | 10.1016/j.jiac.2018.11.005 | NO | NO | PHARMACOLOGICAL STUDY |
| 2019 | A. C. Kalil and A. P. Zavascki | Can ceftolozane–tazobactam treat nosocomial pneumonia? | 10.1016/S1473-3099(19)30523-7 | NO | NO | COMMENT |
| 2017 | S. Kanazawa, R. Nakamura, M. Tsuji, T. Sato and Y. Yamano | In vivo efficacy of cefiderocol against carbapenem-resistant gram- negative bacilli in murine urinary tract infection models shuhei matsumoto, master's degree | 10.1093/ofid/ofx163.1208 | NO | NO | PRECLINICAL STUDY |
| 2017 | S. Kanazawa, T. Sato, N. Kohira, T. Ito-Horiyama, M. Tsuji and Y. Yamano | Susceptibility of imipenem-susceptible but meropenem-resistant blaIMP-6-carrying Enterobacteriaceae to various antibacterials, including the siderophore cephalosporin cefiderocol | 10.1128/AAC.00576-17. | NO | NO | LETTER |
| 2016 | H. Kandil, E. Cramp and T. Vaghela | Trends in Antibiotic Resistance in Urologic Practice | 10.1016/j.euf.2016.09.006 | NO | NO | REVIEW |
| 2019 | N. Kanwar, C. J. Harrison, M. Pence and R. Selvarangan | Comparative in vitro antipseudomonal activity of ceftolozane/ tazobactam against pseudomonas aeruginosa isolates from children with cystic fibrosis | 10.1093/ofid/ofz360.1451 | NO | NO | IN VITRO STUDY |
| 2014 | I. Karaiskos and H. Giamarellou | Multidrug-resistant and extensively drug-resistant Gram-negative pathogens: Current and emerging therapeutic approaches | 10.1517/14656566.2014.914172 | NO | NO | REVIEW |
| 2020 | I. Karaiskos and H. Giamarellou | Carbapenem-sparing strategies for ESBL producers: When and how | 10.3390/antibiotics9020061 | NO | NO | REVIEW |
| 2019 | I. Karaiskos, S. Lagou, K. Pontikis, V. Rapti and G. Poulakou | The "Old" and the "New" Antibiotics for MDR Gram-Negative Pathogens: For Whom, When, and How | 10.3389/fpubh.2019.00151 | NO | NO | REVIEW |
| 2020 | S. Karakonstantis, E. I. Kritsotakis and A. Gikas | Pandrug-resistant gram-negative bacteria: A systematic review of current epidemiology, prognosis and treatment options | 10.1093/jac/dkz401 | NO | NO | SYSTEMATIC REVIEW |
| 2019 | J. A. Karlowsky, M. A. Hackel, M. Tsuji, Y. Yamano, R. Echols and D. F. Sahm | In Vitro Activity of Cefiderocol, a Siderophore Cephalosporin, Against Gram-Negative Bacilli Isolated by Clinical Laboratories in North America and Europe in 2015-2016: SIDERO-WT-2015 | 10.1016/j.ijantimicag.2018.11.007 | NO | NO | IN VITRO STUDY |
| 2020 | J. A. Karlowsky, K. M. Kazmierczak, K. Young, M. R. Motyl and D. F. Sahm | In vitro activity of ceftolozane/tazobactam against phenotypically defined extended-spectrum β-lactamase (ESBL)-positive isolates of Escherichia coli and Klebsiella pneumoniae isolated from hospitalized patients (SMART 2016) | 10.1016/j.diagmicrobio.2019.114925 | NO | NO | IN VITRO STUDY |
| 2020 | J. A. Karlowsky, S. H. Lob, J. Raddatz, D. D. DePestel, K. Young, M. R. Motyl and D. F. Sahm | In Vitro Activity of Imipenem/Relebactam and Ceftolozane/Tazobactam against Clinical Isolates of Gram-Negative Bacilli with Difficult-to-Treat Resistance and Multidrug-Resistant Phenotypes - SMART United States 2015-2017 | 10.1093/cid/ciaa381 | NO | NO | IN VITRO STUDY |
| 2012 | G. Karras, V. Giannakaki, V. Kotsis and S. Miyakis | Novel antimicrobial agents against multi-drug-resistant gram-negative bacteria: An overview | 10.2174/157489112803521922 | NO | NO | REVIEW |
| 2018 | J. Katchanov, L. Asar, E. M. Klupp, A. Both, C. Rothe, C. Konig, H. Rohde, S. Kluge and F. P. Maurer | Carbapenem-resistant Gram-negative pathogens in a German university medical center: Prevalence, clinical implications and the role of novel beta-lactam/beta-lactamase inhibitor combinations | 10.1371/journal.pone.0195757 | YES | NO | DATA NOT RELEVANT |
| 2015 | T. L. Kauf, G. Medic, M. Dryden, P. Xu and M. Zilberberg | Use of surveillance data to examine the cost-effectiveness of alternative approaches to empiric antibiotic therapy in gram-negative nosocomial pneumonia |  | NO | NO | COST-EFFECTIVENESS ANALYSIS |
| 2017 | T. L. Kauf, V. S. Prabhu, G. Medic, R. H. Borse, B. Miller, J. Gaultney, S. S. Sen and A. Basu | Cost-effectiveness of ceftolozane/tazobactam compared with piperacillin/tazobactam as empiric therapy based on the in-vitro surveillance of bacterial isolates in the United States for the treatment of complicated urinary tract infections | 10.1186/s12879-017-2408-7 | NO | NO | COST-EFFECTIVENESS ANALYSIS |
| 2015 | K. S. Kaye and J. M. Pogue | Infections Caused by Resistant Gram-Negative Bacteria: Epidemiology and Management | 10.1002/phar.1636 | NO | NO | REVIEW |
| 2017 | D. Keating, I. Mc Carthy, D. Britton, S. Mc Dermott and K. Schaffer | In vitro susceptibility to ceftolozane/tazobactam in multi drug resistant Pseudomonas aeruginosa |  | NO | NO | IN VITRO STUDY |
| 2015 | W. V. Kern | New antibacterial agents on the market and in the pipeline | 10.1007/s00108-015-3705-0 | NO | NO | REVIEW |
| 2018 | T. Khadem and J. R. Bariola | Does the label matter: Initial use of newly approved antimicrobial agents in community hospitals without robust antimicrobial stewardship programs | 10.1093/ofid/ofy210.1536 | NO | NO | OUT OF TOPIC |
| 2018 | A. Khan, B. Hanson, A. Dinh, A. Wanger, L. Ostrosky-Zeichner, W. Miller and C. Arias | Four superbugs isolated from a single patient in the United States: E. coli (EC) and K. Pneumoniae (KP) harboring NDM-5, P. aeruginosa (PA) harboring NDM-1 and candida auris | 10.1093/ofid/ofy210.712 | NO | NO | CASE REPORT |
| 2018 | A. Khan, W. R. Miller and C. A. Arias | Mechanisms of antimicrobial resistance among hospital-associated pathogens | 10.1080/14787210.2018.1456919 | NO | NO | REVIEW |
| 2020 | A. Khan, W. C. Shropshire, B. Hanson, A. Q. Dinh, A. Wanger, L. Ostrosky-Zeichner, C. A. Arias and W. R. Miller | Simultaneous infection with enterobacteriaceae and pseudomonas aeruginosa harboring multiple carbapenemases in a returning traveler colonized with candida auris | 10.1128/AAC.01466-19 | NO | NO | CASE REPORT |
| 2019 | P. P. Khil, A. D. Chiang, J. Ho, J. H. Youn, J. K. Lemon, J. Gea-Banacloche, K. M. Frank, M. Parta, R. A. Bonomo and J. P. Dekkera | Dynamic emergence of mismatch repair deficiency facilitates rapid evolution of ceftazidime-avibactam resistance in pseudomonas aeruginosa acute infection | 10.1128/mBio.01822-19 | NO | NO | CASE REPORT |
| 2017 | S. Khoshnood, M. Heidary, R. Mirnejad, A. Bahramian, M. Sedighi and H. Mirzaei | Drug-resistant gram-negative uropathogens: A review | 10.1016/j.biopha.2017.08.006 | NO | NO | REVIEW |
| 2019 | M. G. Khrenova, A. V. Krivitskaya and V. G. Tsirelson | The QM/MM-QTAIM approach reveals the nature of the different reactivity of cephalosporins in the active site of L1 metallo-β-lactamase | 10.1039/c9nj00254e | NO | NO | IN VITRO STUDY |
| 2018 | J. M. Kidd, J. L. Kuti and D. P. Nicolau | Novel pharmacotherapy for the treatment of hospital-acquired and ventilator-associated pneumonia caused by resistant gram-negative bacteria | 10.1080/14656566.2018.1438408 | NO | NO | REVIEW |
| 2020 | J. M. Kidd, D. M. Livermore and D. P. Nicolau | The difficulties of identifying and treating Enterobacterales with OXA-48-like carbapenemases | 10.1016/j.cmi.2019.12.006 | NO | NO | COMMENT |
| 2015 | J. T. Kielstein and J. J. Schmidt | Ceftolozane-tazobactam versus levofloxacin in urinary tract infection | 10.1016/S0140-6736(15)00261-5 | NO | NO | COMMENT |
| 2017 | M. S. Kinch | 2014 in review: FDA approval of new drugs | 10.1016/j.drudis.2015.06.004 | NO | NO | REVIEW |
| 2019 | C. S. King, A. W. Brown, S. Aryal, K. Ahmad and S. Donaldson | Critical Care of the Adult Patient With Cystic Fibrosis | 10.1016/j.chest.2018.07.025 | NO | NO | REVIEW |
| 2016 | D. T. King, S. Sobhanifar and N. C. J. Strynadka | One ring to rule them all: Current trends in combating bacterial resistance to the β-lactams | 10.1002/pro.2889 | NO | NO | REVIEW |
| 2019 | A. Kirk, J. Pierce, M. Doll, K. Lee, A. Pakyz, J. Kim, J. D. Markley, G. Bearman and M. P. Stevens | The effectiveness of formulary restriction and preauthorization at an academic medical center | 10.1016/j.ajic.2018.12.026 | NO | NO | OUT OF TOPIC |
| 2018 | T. Kish | New antibiotics in development target highly resistant gram-negative organisms |  | NO | NO | REVIEW |
| 2020 | G. Kleoniki, T. Fotios-Panagiotis, K. Ioannis, P. Konstantinos, K. Emmanuel, P. Eftyxia and L. Moyssis | Disseminated Cryptococcosis in a Non-HIV-Infected Immunocompromised Patient | 10.1016/j.clinmicnews.2020.04.002 | NO | NO | CASE REPORT |
| 2016 | M. Koczorek | Multidrug-resistant gram-negative pathogens: Ceftolozane/tazobactam in complicated intra-abdominal and urinary tract infections | https://www.embase.com/a/#/search/results?subaction=viewrecord&rid=1&page=1&id=L611312317 | NO | NO | NOT IN ENGLISH |
| 2015 | M. H. Kollef | Ventilator-associated pneumonia: The role of emerging therapies and diagnostics | 10.1378/chest.14-2745 | NO | NO | EDITORIAL |
| 2017 | M. H. Kollef | Rebuttal From Dr Kollef | 10.1016/j.chest.2016.11.005 | NO | NO | EDITORIAL |
| 2015 | M. H. Kollef and C. V. Guillamet | If antibiotics did not exist | 10.1007/s00134-014-3533-z | NO | NO | EDITORIAL |
| 2019 | T. Kongnakorn, C. Eckmann, M. Bassetti, E. Tichy, R. Di Virgilio, N. Baillon-Plot and C. Charbonneau | Cost-effectiveness analysis comparing ceftazidime/avibactam (CAZ-AVI) as empirical treatment comparing to ceftolozane/tazobactam and to meropenem for complicated intra-abdominal infection (cIAI) | 10.1186/s13756-019-0652-x | NO | NO | COST-EFFECTIVENESS ANALYSIS |
| 2019 | D. Koulenti, A. Song, A. Ellingboe, M. H. Abdul-Aziz, P. Harris, E. Gavey and J. Lipman | Infections by multidrug-resistant Gram-negative Bacteria: What's new in our arsenal and what's in the pipeline? | 10.1016/j.ijantimicag.2018.10.011 | NO | NO | REVIEW |
| 2018 | J. Kranz, S. Schmidt, C. Lebert, L. Schneidewind, F. Mandraka, M. Kunze, S. Helbig, W. Vahlensieck, K. Naber, G. Schmiemann and F. M. Wagenlehner | The 2017 Update of the German Clinical Guideline on Epidemiology, Diagnostics, Therapy, Prevention, and Management of Uncomplicated Urinary Tract Infections in Adult Patients. Part II: Therapy and Prevention | 10.1159/000487645 | NO | NO | GUIDELINES |
| 2019 | A. Kratzer, U. Rothe and C. Dorn | Stability of ceftolozane/tazobactam in solution as infusion for prolonged or continuous application | 10.1136/ejhpharm-2019-eahpconf.633 | NO | NO | IN VITRO STUDY |
| 2019 | A. Kratzer, S. Schießer, P. Matzneller, B. Wulkersdorfer, M. Zeitlinger, J. Schlossmann, F. Kees and C. Dorn | Determination of total and free ceftolozane and tazobactam in human plasma and interstitial fluid by HPLC-UV | 10.1016/j.jpba.2018.09.044 | NO | NO | IN VITRO STUDY |
| 2020 | M. Kresken, B. Korber-Irrgang, M. Korte-Berwanger, N. Pfennigwerth, S. G. Gatermann and H. Seifert | Dissemination of carbapenem-resistant Pseudomonas aeruginosa isolates and their susceptibilities to ceftolozane-tazobactam in Germany | 10.1016/j.ijantimicag.2020.105959 | NO | NO | IN VITRO STUDY |
| 2020 | N. Krishnan, K. Rechcigl, A. Arcinegas, Z. Vaghaiwalla and G. Kaeley | An open wound with an open question: Is it ecthyma gangrenosum? | 10.1136/jim-2020-SRM.490 | NO | NO | CASE REPORT |
| 2017 | R. Kullar, F. M. Wagenlehner, M. W. Popejoy, J. Long, B. Yu and E. J. Goldstein | Does moderate renal impairment affect clinical outcomes in complicated intra-abdominal and complicated urinary tract infections? Analysis of two randomized controlled trials with ceftolozane/tazobactam | 10.1093/jac/dkw486 | YES | NO | OUT OF TOPIC |
| 2017 | S. T. Kumar, A. Yassin, T. Bhowmick and D. Dixit | Recommendations from the 2016 guidelines for the management of adults with hospital-acquired or ventilator-associated pneumonia |  | NO | NO | GUIDELINES |
| 2020 | S. C. Kuo, C. E. Liu, P. L. Lu, Y. S. Chen, M. C. Lu, W. C. Ko, P. R. Hsueh, Y. C. Chuang and F. D. Wang | Activity of ceftolozane-tazobactam against Gram-negative pathogens isolated from lower respiratory tract infections in the Asia-Pacific region: SMART 2015-2016 | 10.1016/j.ijantimicag.2020.105883 | NO | NO | IN VITRO STUDY |
| 2017 | K. E. Kurtzhalts, K. A. Mergenhagen, A. Manohar and C. S. Berenson | Successful treatment of multidrug-resistant Pseudomonas aeruginosa pubic symphysis osteomyelitis with ceftolozane/tazobactam | 10.1136/bcr-2016-217005 | NO | NO | CASE REPORT |
| 2016 | J. L. Kuti, I. M. Ghazi, R. Quintiliani, E. Shore and D. P. Nicolau | Treatment of multidrug-resistant Pseudomonas aeruginosa with ceftolozane/tazobactam in a critically ill patient receiving continuous venovenous haemodiafiltration | 10.1016/j.ijantimicag.2016.06.005 | NO | NO | CASE REPORT |
| 2015 | J. L. Kuti, R. S. Pettit, N. Neu, J. J. Cies, C. Lapin, M. S. Muhlebach, K. J. Novak, S. T. Nguyen, L. Saiman and D. P. Nicolau | Microbiological activity of ceftolozane/tazobactam, ceftazidime, meropenem, and piperacillin/tazobactam against Pseudomonas aeruginosa isolated from children with cystic fibrosis | 10.1016/j.diagmicrobio.2015.04.012 | NO | NO | IN VITRO STUDY |
| 2018 | C. C. Lai, Y. T. Lin, Y. T. Lin, M. C. Lu, Z. Y. Shi, Y. S. Chen, L. S. Wang, S. H. Tseng, C. N. Lin, Y. H. Chen, W. C. Ko, F. D. Wang and P. R. Hsueh | Clinical characteristics of patients with bacteraemia due to the emergence of mcr-1-harbouring Enterobacteriaceae in humans and pigs in Taiwan | 10.1016/j.ijantimicag.2018.08.015 | NO | NO | IN VITRO STUDY |
| 2018 | E. Lai and J. M. Bender | Antibiotic-resistant bacteria and the use of novel antibiotics in children | 10.3928/19382359-20180807-01 | NO | NO | REVIEW |
| 2017 | E. A. Lakota, J. C. Bader, S. M. Bhavnani, D. R. Andes and P. G. Ambrose | Traditional PK-PD indices for efficacy-can we do better? | 10.1093/ofid/ofx163.685 | NO | NO | PHARMACOLOGICAL STUDY |
| 2017 | S. Lally and L. Giblin | Challenges of vascular access in haemodialysis patients: A case report | 10.1007/s11845-017-1629-5 | NO | NO | CASE REPORT |
| 2016 | M. Lamb | Urinary tract infections: Causes and treatment update |  | NO | NO | REVIEW |
| 2018 | K. LaPlante, J. Cusumano and G. Tillotson | Colistin for the treatment of multidrug-resistant infections | 10.1016/S1473-3099(18)30611-X | NO | NO | COMMENT |
| 2017 | K. Larson, J. S. Bradley, A. Arrieta, S. Yang, B. Yu, M. G. Johnson, M. Rizk and E. Rhee | Plasma pharmacokinetics of ceftolozane/Tazobactam in pediatric subjects with cystic fibrosis | 10.1093/ofid/ofx163.679 | NO | NO | PHARMACOLOGICAL STUDY |
| 2017 | K. Larson, M. G. Johnson, M. L. Rizk, L. Caro, J. S. Bradley, J. Ang and E. Rhee | Ceftolozane/tazobactam dose evaluation for pediatric subjects with complicated intra-abdominal infection and complicated urinary tract infection | 10.1093/ofid/ofx163.1376 | YES | NO | PHARMACOLOGICAL STUDY |
| 2016 | K. Larson, B. Yu, A. Adedoyin, J. A. Huntington, L. Caro, A. Xiao, D. De Lucia, E. Hershberger and E. Rhee | Pharmacokinetics and safety of ceftolozane/tazobactam in adolescents and young children with proven or suspected gram-negative infection | 10.1093/ofid/ofw172.1513 | YES | NO | PHARMACOLOGICAL STUDY |
| 2019 | K. B. Larson, Y. T. Patel, S. Willavize, J. S. Bradley, E. G. Rhee, L. Caro and M. L. Rizk | Ceftolozane-tazobactam population pharmacokinetics and dose selection for further clinical evaluation in pediatric patients with complicated urinary tract or complicated intra-abdominal infections | 10.1128/AAC.02578-18 | NO | NO | PHARMACOLOGICAL STUDY |
| 2017 | K. B. Larson, Y. T. Patel, S. Willavize, M. L. Rizk, B. Yu, M. G. Johnson, E. G. Rhee and L. Caro | Ceftolozane/Tazobactam dose selection for pediatric patients (birth to<18 years) | 10.1007/s10928-017-9536-y | NO | NO | PHARMACOLOGICAL STUDY |
| 2018 | P. Lasalvia, D. Rosselli, C. Castañeda-Cardona, J. Garzon, F. Hernández, C. Beltran, M. Rojas, M. C. Lopez and E. M. Sarpong | Cost-effectiveness of ceftolozane/tazobactam as empiric therapy for the treatment of complicated urinary tract infections in Colombia |  | NO | NO | COST-EFFECTIVENESS ANALYSIS |
| 2019 | K. N. M. Latha and G. R. M. Babu | A novel approach for identification of possible GSK-3β inhibitors using computational virtual screening analysis of drugs | 10.1504/IJCBDD.2019.103596 | NO | NO | OUT OF TOPIC |
| 2019 | A. Lawandi, G. Leite, M. P. Cheng, B. Lefebvre, J. Longtin and T. C. Lee | In vitro synergy of β-lactam combinations against KPC-producing Klebsiella pneumoniae strains | 10.1093/jac/dkz389 | NO | NO | IN VITRO STUDY |
| 2018 | F. Lázaro-Perona, A. Sotillo, P. Troyano-Hernáez, R. Gómez-Gil, Á de la Vega-Bueno and J. Mingorance | Genomic path to pandrug resistance in a clinical isolate of Klebsiella pneumoniae | 10.1016/j.ijantimicag.2018.08.012 | NO | NO | IN VITRO STUDY |
| 2018 | A. C. Lee and A. L. Jones | Multi-resistant Pseudomonas aeruginosa ST235 in cystic fibrosis | 10.1016/j.prrv.2018.05.009 | NO | NO | IN VITRO STUDY |
| 2014 | C. S. Lee and Y. Doi | Therapy of infections due to carbapenem-resistant gram-negative pathogens | 10.3947/ic.2014.46.3.149 | NO | NO | REVIEW |
| 2020 | M. S. L. Lee, B. Ramakrishna, A. C. Moss, H. S. Gold and W. Branch-Elliman | Successful treatment of fulminant Clostridioides difficile infection with emergent fecal microbiota transplantation in a patient with acute myeloid leukemia and prolonged, severe neutropenia | 10.1111/tid.13216 | NO | NO | CASE REPORT |
| 2015 | C. Lee Ventola | The antibiotic resistance crisis: Part 2: Management strategies and new agents |  | NO | NO | REVIEW |
| 2015 | Y. R. Lee, D. McMahan, C. McCall and G. K. Perry | Complicated Intra-Abdominal Infections: The Old Antimicrobials and the New Players | 10.1007/s40265-015-0506-7 | NO | NO | REVIEW |
| 2019 | B. A. Lemkes, O. Richel, M. J. Bonten, P. D. van der Linden and W. J. Wiersinga | [New antibiotics: an overview] |  | NO | NO | REVIEW |
| 2019 | J. R. Lenhard, Z. P. Bulman, B. T. Tsuji and K. S. Kaye | Shifting gears: The future of polymyxin antibiotics | 10.3390/antibiotics8020042 | NO | NO | REVIEW |
| 2019 | M. Leone, J. A. Roberts, M. Bassetti, A. Bouglé, J. P. Lavigne, M. Legrand, M. Neely, J. A. Paiva, D. Payen, J. Rello, C. Roger, F. Sjövall and B. Jung | Update in antibiotic therapy in intensive care unit: report from the 2019 Nîmes International Symposium | 10.1016/j.accpm.2019.09.009 | NO | NO | CONFERENCE PROCEEDINGS |
| 2019 | S. Leone, G. Damiani, I. Pezone, M. E. Kelly, M. Cascella, A. Alfieri, M. C. Pace and M. Fiore | New antimicrobial options for the management of complicated intra-abdominal infections | 10.1007/s10096-019-03533-y | NO | NO | REVIEW |
| 2014 | A. J. Lepak, A. Reda, K. Marchillo, J. V. Hecker, W. A. Craig and D. Andes | Impact of MIC Range for Pseudomonas aeruginosa and Streptococcus pneumoniae on the Ceftolozane in Vivo pharmacokinetic/pharmacodynamic target | 10.1128/AAc.03572-14 | NO | NO | PHARMACOLOGICAL STUDY |
| 2020 | A. Lepape, A. Jean, J. De Waele, A. Friggeri, A. Savey, P. Vanhems, M. P. Gustin, D. L. Monnet, J. Garnacho-Montero and A. Kohlenberg | European intensive care physicians' experience of infections due to antibiotic-resistant bacteria | 10.1186/s13756-019-0662-8 | NO | NO | OUT OF TOPIC |
| 2017 | K. D. Leuthner, R. Kullar, B. Jayakumar, D. A. Hewlett, T. Nguyen and L. Puzniak | Real-world evaluation of ceftolozane/tazobactam (C/T) use and clinical outcomes at an Academic Medical Center in Las Vegas | 10.1093/ofid/ofx163.682 | YES | NO | ABSTRACT |
| 2018 | P. O. Lewis, D. B. Cluck, J. L. Tharp, M. A. Krolikowski and P. D. Patel | Failure of ceftolozane-tazobactam salvage therapy in complicated pneumonia with lung abscess | 10.1002/ccr3.1612 | NO | NO | CASE REPORT |
| 2017 | G. Li Bassi, E. Aguilera, T. Senussi, A. Motos, F. A. Iodone, Y. Hua, R. Martinez Alejos, J. Bobi, R. Amaro, L. Fernandez Barat, O. T. Ranzani, D. Nicolau, C. Sutherland, P. Pelosi, M. Antonelli and A. Torres | Bactericidal efficacy of ceftolozane/tazobactam in comparison with piperacillin/tazobactam in a model of severe pseudomonas aeruginosa pneumonia | 10.1164/ajrccm-conference.2017.B61 | NO | NO | PRECLINICAL STUDY |
| 2017 | G. Li Bassi, E. Aguilera Xiol, A. Motos, F. Pagliara, J. Bobi, T. Senussi, F. Idone, R. Amaro, L. Fernandez, M. Rigol, O. Ranzani, D. Nicolau, C. Sutherland, P. Pelosi, M. Antonelli and A. Torres | The effects of ceftolozane/tazobactam vs. piperacillin/tazobactam in a model of multi-resistant Pseudomonas aeruginosa pneumonia | 10.1186/s40635-017-0151-4 | NO | NO | PRECLINICAL STUDY |
| 2017 | G. Li Bassi, D. Nicolau, T. Senussi, A. Motos, C. Sutherland, F. A. Iodone, Y. Hua, R. Martinez Alejos, Q. Bobi, O. T. Ranzani, P. Pelosi, M. Antonelli and A. Torres | Characterization of the pharmacokinetic/pharmacodynamic profile of ceftolozane in an animal model of severe p. aeruginosa pneumonia | 10.1164/ajrccm-conference.2017.B61 | NO | NO | PHARMACOLOGICAL STUDY |
| 2012 | H. Li, Y. F. Luo, B. J. Williams, T. S. Blackwell and C. M. Xie | Structure and function of OprD protein in Pseudomonas aeruginosa: From antibiotic resistance to novel therapies | 10.1016/j.ijmm.2011.10.001 | NO | NO | CASE REPORT |
| 2020 | X. Li, K. Xiao, Z. Zhang, J. Yang, R. Wang, X. Shen, J. Pan, D. M. Irwin, R. A. Chen and Y. Shen | The recombination hot spots and genetic diversity of the genomes of African swine fever viruses | 10.1016/j.jinf.2019.08.007 | NO | NO | OUT OF TOPIC |
| 2019 | C. H. Liao, N. Y. Lee, H. J. Tang, S. S. Lee, C. F. Lin, P. L. Lu, J. J. Wu, W. C. Ko, W. S. Lee and P. R. Hsueh | Antimicrobial activities of ceftazidime-avibactam, ceftolozane-tazobactam, and other agents against Escherichia coli, Klebsiella pneumoniae, and Pseudomonas aeruginosa isolated from intensive care units in Taiwan: results from the Surveillance of Multicenter Antimicrobial Resistance in Taiwan in 2016 | 10.2147/idr.S193638 | NO | NO | IN VITRO STUDY |
| 2019 | J. Liao, H. Sheng, J. Sauri, R. Xiang and G. Martin | Structural elucidation of a dimeric impurity in the process development of ceftolozane using LC/HRMS and 2D-NMR | 10.1016/j.jpba.2019.05.057 | NO | NO | IN VITRO STUDY |
| 2019 | A. Liapikou | New antibiotics for hospital-acquired pneumonia |  | NO | NO | REVIEW |
| 2016 | A. Liapikou and A. Torres | Emerging drugs for nosocomial pneumonia | 10.1080/14728214.2016.1206077 | NO | NO | REVIEW |
| 2018 | B. Lima, S. Bodeau, L. Simon, C. Delette, Y. Zerbib, C. Lecaque, H. Dupont, K. Masmoudi, A. S. Lemaire-Hurtel and Y. Bennis | Validation of a simple and rapid HPLC-DAD method for the routine therapeutic drug monitoring of ceftolozane in critically ill patients | 10.1111/fcp.12371 | NO | NO | IN VITRO STUDY |
| 2017 | L. Y. Lin, M. Vail, D. Debabov and I. Critchley | Comparative in vitro activities of ceftazidime-avibactam and ceftolozane-tazobactam against characterized β-lactamase-producing pseudomonas aeruginosa | 10.1093/ofid/ofx163.897 | NO | NO | IN VITRO STUDY |
| 2016 | S. Y. Lin, C. H. Huang, W. C. Ko, Y. H. Chen and P. R. Hsueh | Recent developments in antibiotic agents for the treatment of complicated intra-abdominal infections | 10.1517/14656566.2016.1122756 | NO | NO | REVIEW |
| 2019 | W. T. Lin, C. C. Lai and C. U. Cheong | Novel β-lactam/β-lactamase combination versus meropenem for treating nosocomial pneumonia | 10.3390/antibiotics8040219 | NO | NO | REVIEW |
| 2017 | D. Lindquist, J. Burchette, B. Odle and D. Cluck | New antibiotics – old problems | 10.1111/ijpp.12261 | NO | NO | LETTER |
| 2019 | B. Lipový and M. Hanslianová | Efficacy of new cephalosporins in treatment of multidrug-resistant strains of gram-negative bacteria in burn patients | 10.1016/j.burns.2019.06.005 | NO | NO | COMMENT |
| 2019 | B. Lipový, M. Hanslianová, Y. Kaloudová, H. Řihová, M. Knoz, J. Holoubek and I. Suchánek | Ceftolozane/tazobactam in treating multifocal infection caused by a multiresistant pseudomonas aeruginosa strain in a severely burned patient |  | NO | NO | CASE REPORT |
| 2017 | J. LiPuma | Newly available broad-spectrum agents | 10.1002/ppul.23839 | NO | NO | REVIEW |
| 2015 | J. L. Liscio, M. V. Mahoney and E. B. Hirsch | Ceftolozane/tazobactam and ceftazidime/avibactam: Two novel β-lactam/β-lactamase inhibitor combination agents for the treatment of resistant Gram-negative bacterial infections | 10.1016/j.ijantimicag.2015.05.003 | NO | NO | REVIEW |
| 2014 | D. M. Livermore | Of stewardship, motherhood and apple pie | 10.1016/j.ijantimicag.2014.01.011 | NO | NO | COMMENT |
| 2015 | D. M. Livermore | What's new in epidemiology of extremely resistant Gram-negative pathogens? | http://www.embase.com/search/results?subaction=viewrecord&from=export&id=L71916065 | NO | NO | CONFERENCE PROCEEDINGS |
| 2017 | D. M. Livermore | Novel antibiotics on the horizon | https://www.embase.com/a/#/search/results?subaction=viewrecord&rid=1&page=1&id=L622094096 | NO | NO | CONFERENCE PROCEEDINGS |
| 2018 | D. M. Livermore, D. Meunier, K. L. Hopkins, M. Doumith, R. Hill, R. Pike, P. Staves and N. Woodford | Activity of ceftazidime/avibactam against problem Enterobacteriaceae and Pseudomonas aeruginosa in the UK, 2015-16 | 10.1093/jac/dkx438 | NO | NO | IN VITRO STUDY |
| 2010 | D. M. Livermore, S. Mushtaq and Y. Ge | Chequerboard titration of cephalosporin CXA-101 (FR264205) and tazobactam versus β-lactamase-producing Enterobacteriaceae | 10.1093/jac/dkq248 | NO | NO | IN VITRO STUDY |
| 2009 | D. M. Livermore, S. Mushtaq, Y. Ge and M. Warner | Activity of cephalosporin CXA-101 (FR264205) against Pseudomonas aeruginosa and Burkholderia cepacia group strains and isolates | 10.1016/j.ijantimicag.2009.03.021 | NO | NO | IN VITRO STUDY |
| 2017 | D. M. Livermore, S. Mushtaq, D. Meunier, K. L. Hopkins, R. Hill, R. Adkin, A. Chaudhry, R. Pike, P. Staves and N. Woodford | Activity of ceftolozane/tazobactam against surveillance and 'problem' Enterobacteriaceae, Pseudomonas aeruginosa and non-fermenters from the British Isles | 10.1093/jac/dkx136 | NO | NO | IN VITRO STUDY |
| 2020 | O. Ljungquist, C. Kampmann, F. Resman, K. Riesbeck and J. Tham | Probiotics for intestinal decolonization of ESBL-producing Enterobacteriaceae: a randomized, placebo-controlled clinical trial | 10.1016/j.cmi.2019.08.019 | NO | NO | OUT OF TOPIC |
| 2010 | L. I. Llarrull, S. A. Testero, J. F. Fisher and S. Mobashery | The future of the β-lactams | 10.1016/j.mib.2010.09.008 | NO | NO | REVIEW |
| 2015 | S. Lo Menzo, G. la Martire, G. Ceccarelli and M. Venditti | New insight on epidemiology and management of bacterial bloodstream infection in patients with hematological malignancies | 10.4084/MJHID.2015.044 | NO | NO | REVIEW |
| 2017 | S. Lob, M. Hackel, R. Badal, K. Young, M. Motyl and D. Sahm | Activity of ceftolozane-tazobactam against global pseudomonas aeruginosa and non-susceptible phenotypes: Smart 2016 | 10.1093/ofid/ofx163.940 | NO | NO | IN VITRO STUDY |
| 2019 | S. Lob, K. Kazmierczak, D. DePestel, J. Raddatz, K. Young, M. Motyl and D. F. Sahm | Cross-resistance of ceftolozane-tazobactam and imipenem-relebactam against clinical P. aeruginosa isolates: SMART United States 2016-2018 | 10.1093/ofid/ofz360.666 | NO | NO | IN VITRO STUDY |
| 2019 | S. Lob, K. Kazmierczak, J. Raddatz, D. DePestel, K. Young, M. Motyl and D. F. Sahm | Activity of ceftolozane-tazobactam and ceftazidime-avibactam against clinical P. Aeruginosa Isolates COLLECTED IN United States and CanadaSMART 2018 | 10.1093/ofid/ofz360.786 | NO | NO | IN VITRO STUDY |
| 2020 | S. H. Lob, D. J. Hoban, K. Young, M. R. Motyl and D. F. Sahm | Activity of ceftolozane–tazobactam and comparators against Pseudomonas aeruginosa from patients in different risk strata – SMART United States 2016–2017 | 10.1016/j.jgar.2019.07.017 | NO | NO | IN VITRO STUDY |
| 2019 | O. Lomovskaya, J. Lindley, D. Rubio-Aparicio, K. J. Nelson and M. Castanheira | In vitro activity of the β-lactamase inhibitor QPX7728 in combination with several β-lactams against acinetobacter baumannii (AB) and pseudomonas aeruginosa (PSA) | 10.1093/ofid/ofz360.749 | NO | NO | IN VITRO STUDY |
| 2020 | O. Lomovskaya, R. Tsivkovski, K. Nelson, D. Rubio-Aparicio, D. Sun, M. Totrov and M. N. Dudley | Spectrum of Beta-Lactamase Inhibition by the Cyclic Boronate QPX7728, an Ultra-Broad-Spectrum Beta-lactamase Inhibitor of Serine and Metallo Beta-Lactamases: Enhancement of Activity of Multiple Antibiotics Against Isogenic Strains Expressing Single β-lactamases | 10.1128/AAC.00212-20 | NO | NO | IN VITRO STUDY |
| 2014 | T. E. Long and J. T. Williams | Cephalosporins currently in early clinical trials for the treatment of bacterial infections | 10.1517/13543784.2014.930127 | NO | NO | REVIEW |
| 2019 | C. Longshaw, M. Tsuji, M. M. Hackel, D. F. Sahm and Y. Yamano | In vitro activity of cefiderocol (CFDC), a novel siderophore cephalosporin, against difficult-to-treat-resistant (DTR) gram-negative bacterial pathogens from the multi-national sentinel surveillance study, sidero-WT (2014-2017) | 10.1093/ofid/ofz360.747 | NO | NO | IN VITRO STUDY |
| 2019 | A. I. López-Calleja, E. M. Morales, R. N. Medina, M. F. Esgueva, J. S. Pareja, J. M. G. L. Moya, I. F. Cerón, J. V. Bayon and A. R. López | Antimicrobial activity of ceftolozane-tazobactam against multidrug-resistant and extensively drug-resistant Pseudomonas aeruginosa clinical isolates from a Spanish hospital |  | NO | NO | IN VITRO STUDY |
| 2018 | C. López-Causapé, R. Rubio, G. Cabot and A. Oliver | Evolution of the Pseudomonas aeruginosa aminoglycoside mutational resistome in vitro and in the cystic fibrosis setting | 10.1128/AAC.02583-17 | NO | NO | IN VITRO STUDY |
| 2019 | E. López-Jácome, R. Franco-Cendejas, H. Quezada, R. Morales-Espinosa, I. Castillo-Juárez, B. González-Pedrajo, A. M. Fernández-Presas, A. Tovar-García, V. Angarita-Zapata, P. Licona-Limón, M. Martínez-Vázquez and R. García-Contreras | The race between drug introduction and appearance of microbial resistance. Current balance and alternative approaches | 10.1016/j.coph.2019.04.016 | NO | NO | REVIEW |
| 2020 | I. Los-Arcos, J. Burgos, V. Falco and B. Almirante | An overview of ceftolozane sulfate + tazobactam for treating hospital acquired pneumonia | 10.1080/14656566.2020.1739269 | NO | NO | REVIEW |
| 2014 | C. Lucasti, E. Hershberger, B. Miller, S. Yankelev, J. Steenbergen, I. Friedland and J. Solomkin | Multicenter, double-blind, randomized, phase II trial to assess the safety and efficacy of ceftolozane-tazobactam plus metronidazole compared with meropenem in adult patients with complicated intra-abdominal infections | 10.1128/AAC.00049-14 | YES | NO | DATA NOT RELEVANT |
| 2017 | K. H. Luepke, K. J. Suda, H. Boucher, R. L. Russo, M. W. Bonney, T. D. Hunt and J. F. Mohr | Past, Present, and Future of Antibacterial Economics: Increasing Bacterial Resistance, Limited Antibiotic Pipeline, and Societal Implications | 10.1002/phar.1868 | NO | NO | REVIEW |
| 2018 | A. H. Ma and G. J. Hughes | Updates in Management of Complicated Urinary Tract Infections: A Focus on Multidrug-Resistant Organisms | 10.1097/MJT.0000000000000683 | NO | NO | REVIEW |
| 2016 | A. P. MacGowan, A. R. Noel, S. G. Tomaselli, D. Nicholls and K. E. Bowker | Pharmacodynamics of ceftolozane plus tazobactam studied in an In Vitro pharmacokinetic model of infection | 10.1128/AAC.00727-15 | NO | NO | IN VITRO STUDY |
| 2017 | S. H. Macvane | Antimicrobial Resistance in the Intensive Care Unit | 10.1177/0885066615619895 | NO | NO | REVIEW |
| 2017 | S. H. MacVane, R. Pandey, L. L. Steed, B. N. Kreiswirth and L. Chen | Emergence of ceftolozane-tazobactam-resistant Pseudomonas aeruginosa during treatment is mediated by a single AmpC structural mutation | 10.1128/AAC.01183-17 | NO | NO | CASE REPORT |
| 2019 | S. Maddocks, A. P. Fabijan, J. Ho, R. C. Y. Lin, N. L. Ben Zakour, C. Dugan, I. Kliman, S. Branston, S. Morales and J. R. Iredell | Bacteriophage therapy of ventilator-associated pneumonia and empyema caused by pseudomonas aeruginosa | 10.1164/rccm.201904-0839LE | NO | NO | CASE REPORT |
| 2020 | A. Mahmoud, A. Shah, K. Nutley, D. P. Nicolau, C. Sutherland, M. Jain, M. H. Scheetz and N. J. Rhodes | Clinical pharmacokinetics of ceftolozane and tazobactam in an obese patient receiving continuous venovenous haemodiafiltration: A patient case and literature review | 10.1016/j.jgar.2020.03.003 | NO | NO | CASE REPORT |
| 2017 | A. Majeed, S. Alarfaj, R. Darouiche and M. Mohajer | An update on emerging therapies for urinary tract infections | 10.1080/14728214.2017.1293650 | NO | NO | REVIEW |
| 2019 | M. Malan, W. Xuejingzi, J. Si and S. J. Quan | Hailey-hailey disease: The role of azathioprine an immunomodulator | 10.11604/pamj.2019.32.65.17877 | NO | NO | CASE REPORT |
| 2019 | S. Mancini, P. M. Keller, M. Greiner, V. Bruderer and F. Imkamp | Detection of NDM-19, a novel variant of the New Delhi metallo-β-lactamase with increased carbapenemase activity under zinc-limited conditions, in Switzerland | 10.1016/j.diagmicrobio.2019.06.003 | NO | NO | CASE REPORT |
| 2018 | S. Mancini, L. Poirel, M. Corthesy, G. Greub and P. Nordmann | Klebsiella pneumoniae co-producing KPC and RmtG, finally targeting Switzerland | 10.1016/j.diagmicrobio.2017.10.019 | NO | NO | CASE REPORT |
| 2019 | D. Mangioni, B. Viaggi, T. Giani, F. Arena, S. D'Arienzo, S. Forni, G. Tulli and G. M. Rossolini | Diagnostic stewardship for sepsis: The need for risk stratification to triage patients for fast microbiology workflows | 10.2217/fmb-2018-0329 | NO | NO | COMMENT |
| 2018 | M. Mantero, P. Rogliani, M. Cazzola, F. Blasi and M. Di Pasquale | Emerging antibacterial and antiviral drugs for treating respiratory tract infections | 10.1080/14728214.2018.1504020 | NO | NO | REVIEW |
| 2017 | A. E. Maraolo, M. Cascella, S. Corcione, A. Cuomo, S. Nappa, G. Borgia, F. G. De Rosa and I. Gentile | Management of multidrug-resistant Pseudomonas aeruginosa in the intensive care unit: state of the art | 10.1080/14787210.2017.1367666 | NO | NO | REVIEW |
| 2018 | A. E. Maraolo, M. Cascella, S. Corcione, A. Cuomo, S. Nappa, G. Borgia, F. G. De Rosa and I. Gentile | Response to: ‘Letter to the Editor: “Management of multidrug-resistant Pseudomonas aeruginosa in the Intensive Care Unit: state of the art”’ | 10.1080/14787210.2018.1473082 | NO | NO | LETTER |
| 2020 | A. E. Maraolo, M. Mazzitelli, E. M. Trecarichi, A. R. Buonomo, C. Torti and I. Gentile | Ceftolozane/tazobactam for difficult-to-treat Pseudomonas aeruginosa infections: A systematic review of its efficacy and safety for off-label indications | 10.1016/j.ijantimicag.2020.105891 | NO | NO | SYSTEMATIC REVIEW |
| 2010 | F. Marinelli and A. Tomasz | Antimicrobials | 10.1016/j.mib.2010.09.010 | NO | NO | EDITORIAL |
| 2011 | B. L. Mark, D. J. Vocadlo and A. Oliver | Providing β-lactams a helping hand: Targeting the AmpC β-lactamase induction pathway | 10.2217/fmb.11.128 | NO | NO | REVIEW |
| 2016 | A. M. Marr, A. Morgan and M. Salas | The importance of postmarketing surveillance in the identification of early safety signals of medication errors in patients receiving a new combination antibiotic | 10.1093/ofid/ofw172.1349 | NO | NO | OUT OF TOPIC |
| 2019 | E. Marrero, R. Delahanty and J. Alvarez | Mortality and los comparison: Ceftazidime/avibactam or ceftolozane/ tazobactam vs traditional therapy | <http://www.embase.com/search/results?subaction=viewrecord&from=export&id=L629628244> | YES | NO | CONFERENCE PROCEEDINGS |
| 2016 | H. D. Marston, D. M. Dixon, J. M. Knisely, T. N. Palmore and A. S. Fauci | Antimicrobial resistance | 10.1001/jama.2016.11764 | NO | NO | REVIEW |
| 2018 | T. Marston, K. J. Henry and R. Garcia-Orr | Permissive hypoxemia on ECMO in a patient with granulomatosis with polyangiitis and MDR pseudomonas superinfection |  | NO | NO | CASE REPORT |
| 2020 | J. Martens-Lobenhoffer, M. Hinderhofer, U. Troger and S. M. Bode-Boger | Stability of ceftolozane in human plasma and dried blood spots: Implications for transport and storage | 10.1016/j.vascn.2020.106692 | NO | NO | IN VITRO STUDY |
| 2019 | M. Martin-Cazana, S. Grau, C. Epalza, P. Branas, M. Flores, M. Olmedilla and D. Blazquez-Gamero | Successful ceftolozane-tazobactam rescue therapy in a child with endocarditis caused by multidrug-resistant Pseudomonas aeruginosa | 10.1111/jpc.14388 | NO | NO | CASE REPORT |
| 2020 | J. M. Martin-Guerra and M. Martin-Asenjo | Reply to "Hypokalaemia probably associated with ceftolozane/tazobactam treatment: Three case reports" | 10.1016/j.eimc.2019.09.004 | NO | NO | LETTER |
| 2016 | B. C. Martini | Ceftolozane/tazobactam is more effective than levofloxacin | http://www.embase.com/search/results?subaction=viewrecord&from=export&id=L607796098 | YES | NO | NOT IN ENGLISH |
| 2014 | E. Maseda, L. Aguilar, M. J. Gimenez and F. Gilsanz | Ceftolozane/tazobactam (CXA 201) for the treatment of intra-abdominal infections | 10.1586/14787210.2014.950230 | NO | NO | REVIEW |
| 2019 | C. Massip, C. Mathieu, C. Gaudru, V. Miaut, P. Floch, E. Oswald, C. Segonds and H. Guet-Revillet | In vitro activity of seven β-lactams including ceftolozane/tazobactam and ceftazidime/avibactam against Burkholderia cepacia complex, Burkholderia gladioli and other non-fermentative Gram-negative bacilli isolated from cystic fibrosis patients | 10.1093/jac/dky423 | NO | NO | IN VITRO STUDY |
| 2019 | M. Matt, V. Pagis, C. Duran, F. Bouchand, L. Noussair, M. de Laroche, A. Roux, M. Rottman, B. Davido and A. Dinh |  | 10.1016/j.medmal.2019.04.228 | YES | NO | NOT IN ENGLISH |
| 2017 | D. M. Mazer, C. Young, L. M. Kalikin, T. Spilker and J. J. LiPuma | In vitro activity of ceftolozane-tazobactam and other antimicrobial agents against burkholderia cepacia complex and burkholderia gladioli | 10.1128/AAC.00766-17 | NO | NO | IN VITRO STUDY |
| 2017 | M. Mazer-Amirshahi, A. Pourmand and L. May | Newly approved antibiotics and antibiotics reserved for resistant infections: Implications for emergency medicine | 10.1016/j.ajem.2016.10.034 | NO | NO | REVIEW |
| 2017 | J. E. Mazuski, J. M. Tessier, A. K. May, R. G. Sawyer, E. P. Nadler, M. R. Rosengart, P. K. Chang, P. J. O’Neill, K. P. Mollen, J. M. Huston, J. J. Diaz and J. M. Prince | The surgical infection society revised guidelines on the management of intra-abdominal infection | 10.1089/sur.2016.261 | NO | NO | GUIDELINES |
| 2019 | M. G. McCracken, H. J. Adam, J. M. Blondeau, A. J. Walkty, J. A. Karlowsky, D. J. Hoban, G. G. Zhanel and M. R. Mulvey | Characterization of carbapenem-resistant and XDR Pseudomonas aeruginosa in Canada: results of the CANWARD 2007-16 study | 10.1093/jac/dkz285 | NO | NO | IN VITRO STUDY |
| 2016 | K. E. McHugh, D. D. Rhoads, D. A. Wilson, K. B. Highland, S. S. Richter and G. W. Procop | Inquilinus limosus in pulmonary disease: case report and review of the literature | 10.1016/j.diagmicrobio.2016.09.006 | NO | NO | CASE REPORT |
| 2016 | J. A. McKinnell, J. Hindler, E. Epson, S. Horwich-Scholefield, L. Miller, J. Mendez, J. Martinez, J. Sinkowitz, D. Terashita, P. Marquez, S. Bhaurla, M. Moran, L. Pandes, C. Hershey and R. Humphries | Incomplete adoption of clinical laboratory standards institute breakpoints to detect Carbapenem-resistant organisms | 10.1093/ofid/ofw172.221 | NO | NO | OUT OF TOPIC |
| 2015 | M. J. Melchers, E. Mavridou, S. Seyedmousavi, A. C. Van Mil, C. Lagarde and J. W. Mouton | Plasma and epithelial lining fluid pharmacokinetics of ceftolozane and tazobactam alone and in combination in mice | 10.1128/AAC.04402-14 | NO | NO | PRECLINICAL STUDY |
| 2016 | M. J. Melchers, E. Mavridou, A. C. Van Mil, C. Lagarde and J. W. Mouton | Pharmacodynamics of ceftolozane combined with tazobactam against Enterobacteriaceae in a neutropenic mouse thigh model | 10.1128/AAC.01580-16 | NO | NO | PRECLINICAL STUDY |
| 2017 | M. J. Melchers, A. C. Van Mil, C. Lagarde, J. D. Hartigh and J. W. Mouton | Pharmacodynamics of cefepime combined with tazobactam against clinically relevant enterobacteriaceae in a neutropenic mouse thigh model | 10.1128/AAC.00267-17 | NO | NO | PRECLINICAL STUDY |
| 2015 | M. J. Melchers, A. C. van Mil and J. W. Mouton | In Vitro Activity of Ceftolozane Alone and in Combination with Tazobactam against Extended-Spectrum-beta-Lactamase-Harboring Enterobacteriaceae | 10.1128/aac.04498-14 | NO | NO | IN VITRO STUDY |
| 2017 | L. Meng, E. Mui, M. K. Holubar and S. C. Deresinski | Comprehensive Guidance for Antibiotic Dosing in Obese Adults | 10.1002/phar.2023 | NO | NO | GUIDELINES |
| 2017 | F. Menichetti, S. Giuliano and S. Fortunato | Are there any reasons to change our behavior in necrotizing fasciitis with the advent of new antibiotics? | 10.1097/QCO.0000000000000359 | NO | NO | REVIEW |
| 2018 | J. Mensa, J. Barberán, A. Soriano, P. Llinares, F. Marco, R. Cantón, G. Bou, J. G. del Castillo, E. Maseda, J. R. Azanza, J. Pasquau, C. García-Vidal, J. M. Reguera, D. Sousa, J. Gómez, M. Montejo, M. Borges, A. Torres, F. Alvarez-Lerma, M. Salavert, R. Zaragoza and A. Oliver | Antibiotic selection in the treatment of acute invasive infections by pseudomonas aeruginosa: Guidelines by the Spanish society of chemotherapy |  | NO | NO | GUIDELINES |
| 2016 | S. Merchant, E. Rhee, J. Hawkshead, R. Agarwal, V. Kapoor and N. Cossrow | Real-world evaluation of ceftolozane/ tazobactam in a large cohort of hospitals in the United States | 10.1097/01.ccm.0000509350.03675.fa | YES | NO | NOT COMPARABLE DATA |
| 2017 | K. Meyer, M. Santarossa, L. H. Danziger and E. Wenzler | Compatibility of ceftazidime-avibactam, ceftolozane- tazobactam, and piperacillin-tazobactam with vancomycin in dextrose 5% in water | 10.1310/hpj5203-221 | NO | NO | OUT OF TOPIC |
| 2015 | A. Micklus and S. Muntner | Market watch: Biopharma deal-making in 2014: A record year for M&A value | 10.1038/nrd4536 | NO | NO | OUT OF TOPIC |
| 2019 | J. K. Midturi and S. Ranganath | Prevention and Treatment of Multidrug-Resistant Organisms in End-Stage Renal Disease | 10.1053/j.ackd.2018.09.003 | NO | NO | REVIEW |
| 2019 | H. Mikamo, K. Monden, Y. Miyasaka, T. Horiuchi, G. Fujimoto, T. Fukuhara, T. Yoshinari, E. G. Rhee and T. Shizuya | The efficacy and safety of tazobactam/ceftolozane in combination with metronidazole in Japanese patients with complicated intra-abdominal infections | 10.1016/j.jiac.2018.10.012 | YES | NO | NOT COMPARABLE DATA |
| 2016 | H. Mikamo, A. Yuasa, K. Wada, B. Crawford and N. Sugimoto | Optimal treatment for complicated intra-abdominal infections in the era of antibiotic resistance: A systematic review and meta-analysis of the efficacy and safety of combined therapy with metronidazole | 10.1093/ofid/ofw143 | NO | NO | SYSTEMATIC REVIEW |
| 2016 | G. Mikucionyte, L. Zamorano, A. Vitkauskiene, C. López-Causapé, C. Juan, X. Mulet and A. Oliver | Nosocomial dissemination of VIM-2-producing ST235 Pseudomonas aeruginosa in Lithuania | 10.1007/s10096-015-2529-0 | NO | NO | IN VITRO STUDY |
| 2012 | B. Miller, E. Hershberger, D. Benziger, M. Trinh and I. Friedland | Pharmacokinetics and safety of intravenous ceftolozane-tazobactam in healthy adult subjects following single and multiple ascending doses | 10.1128/AAC.06349-11 | NO | NO | PHARMACOLOGICAL STUDY |
| 2017 | J. E. Miller, M. Wilenzick, N. Ritcey, J. S. Ross and M. M. Mello | Measuring clinical trial transparency: An empirical analysis of newly approved drugs and large pharmaceutical companies | 10.1136/bmjopen-2017-017917 | NO | NO | OUT OF TOPIC |
| 2019 | M. Mills, A. MacWhinnie and T. Do | Evaluating the impact of ceftolozane/tazobactam on clinical outcomes in patients with multi-drug-resistant pseudomonas aeruginosa pneumonia | 10.1093/ofid/ofz360.1964 | YES | NO | ABSTRACT |
| 2020 | H. C. Mirza, E. Hortac, A. A. Kocak, M. H. Demirkaya, B. Yayla, A. U. Guclu and A. Basustaoglu | In vitro activity of ceftolozane-tazobactam and ceftazidime-avibactam against clinical isolates of meropenem-non-susceptible Pseudomonas aeruginosa: A two-centre study | 10.1016/j.jgar.2019.09.016 | NO | NO | IN VITRO STUDY |
| 2018 | A. Mischnik, C. Lubbert and N. T. Mutters | [New betalactam antibiotics and betalactamase inhibitors against multidrug-resistant Gram-negative bacteria] | 10.1007/s00108-018-0508-0 | NO | NO | REVIEW |
| 2018 | S. Mitchell and R. M. Humphries | New and Novel Agents Targeting Resistant Gram-Negative Bacteria: A Review for the Clinical Microbiologist | 10.1016/j.clinmicnews.2018.08.007 | NO | NO | REVIEW |
| 2019 | Y. Mo, M. Lorenzo, S. Farghaly, K. Kaur and S. T. Housman | What's new in the treatment of multidrug-resistant gram-negative infections? | 10.1016/j.diagmicrobio.2018.08.007 | NO | NO | REVIEW |
| 2019 | A. R. Modi, N. S. Majhail, L. Rybicki, V. Athans, K. Carlstrom, P. Srinivas, D. M. Lang, R. Sobecks and C. S. Kovacs | Penicillin allergy skin testing as an antibiotic stewardship intervention reduces alternative antibiotic exposures in hematopoietic stem cell transplant recipients | 10.1111/tid.13175 | NO | NO | OUT OF TOPIC |
| 2018 | S. Mofidifar, F. Sohraby, M. Bagheri and H. Aryapour | Repurposing existing drugs for new AMPK activators as a strategy to extend lifespan: a computer-aided drug discovery study | 10.1007/s10522-018-9744-x | NO | NO | PHARMACOLOGICAL STUDY |
| 2019 | M. F. Mojica, R. Rios, E. De La Cadena, A. Correa, L. Diaz, L. V. Millan, A. K. Hernandez, J. Reyes, C. Hernández-Gómez, M. A. Radice, P. Castañeda-Méndez, D. A. Jaime-Villalón, A. C. Gales, J. M. Munita, C. López, M. M. R. Rojas and M. V. Villegas | Molecular mechanisms leading to ceftolozane-tazobactam resistance in clinical isolates of pseudomonas aeruginosa from five latin american countries | 10.1093/ofid/ofz360.681 | NO | NO | IN VITRO STUDY |
| 2018 | L. Molloy, I. Abdulhamid, R. Srivastava and J. Ang | Effectiveness and safety of ceftolozane/tazobactam (TOL/TAZ) use for carbapenem-resistant pseudomonas infections in children | 10.1093/ofid/ofy210.2076 | YES | NO | ABSTRACT |
| 2020 | L. Molloy, I. Abdulhamid, R. Srivastava and J. Y. Ang | Ceftolozane/tazobactam treatment of multidrug-resistant pseudomonas aeruginosa infections in children | 10.1097/INF.0000000000002593 | NO | NO | CASE REPORT |
| 2017 | E. Molnar, E. Heil, K. Claeys, J. Hiles and J. Gallagher | Ceftolozane-tazobactam for the treatment of multi drug-resistant pseudomonas aeruginosa (mdrpa) infections | 10.1093/ofid/ofx163.643 | NO | NO | ABSTRACT |
| 2018 | F. Monaco, G. D. Mento, N. Cuscino, P. G. Conaldi and B. Douradinha | Infant colonisation with Escherichia coli and Klebsiella pneumoniae strains co-harbouring blaOXA-48 and blaNDM-1 carbapenemases genes: a case report | 10.1016/j.ijantimicag.2018.04.018 | NO | NO | CASE REPORT |
| 2017 | M. Monogue and D. P. Nicolau | In vitro antibacterial activity of ceftolozane/tazobactam (C/T) alone and in combination with other antimicrobial agents against multidrug-resistant (MDR) Pseudomonas aeruginosa (PSA) | 10.1093/ofid/ofx163.688 | NO | NO | IN VITRO STUDY |
| 2016 | M. L. Monogue, J. L. Kuti and D. P. Nicolau | Optimizing Antibiotic Dosing Strategies for the Treatment of Gram-negative Infections in the Era of Resistance | 10.1586/17512433.2016.1133286 | NO | NO | PHARMACOLOGICAL STUDY |
| 2018 | M. L. Monogue and D. P. Nicolau | Antibacterial activity of ceftolozane/tazobactam alone and in combination with other antimicrobial agents against MDR Pseudomonas aeruginosa | 10.1093/jac/dkx483 | NO | NO | PHARMACOLOGICAL STUDY |
| 2019 | M. L. Monogue and D. P. Nicolau | Pharmacokinetics-pharmacodynamics of β-lactamase inhibitors: are we missing the target? | 10.1080/14787210.2019.1647781 | NO | NO | REVIEW |
| 2016 | M. L. Monogue, R. S. Pettit, M. Muhlebach, J. J. Cies, D. P. Nicolau and J. L. Kuti | Population pharmacokinetics and safety of ceftolozane-tazobactam in adult cystic fibrosis patients admitted with acute pulmonary exacerbation | 10.1128/AAC.01566-16 | NO | NO | PHARMACOLOGICAL STUDY |
| 2017 | M. L. Monogue, S. M. Stainton, A. Baummer-Carr, A. K. Shepard, J. F. Nugent, J. L. Kuti and D. P. Nicolau | Pharmacokinetics and tissue penetration of ceftolozane-tazobactam in diabetic patients with lower limb infections and healthy adult volunteers | 10.1128/AAC.01449-17 | NO | NO | PHARMACOLOGICAL STUDY |
| 2019 | M. L. Monogue, J. Tabor-Rennie, K. Abdelraouf and D. P. Nicolau | In Vivo Efficacy of WCK 5222 (Cefepime-Zidebactam) against Multidrug-Resistant Pseudomonas aeruginosa in the Neutropenic Murine Thigh Infection Model | 10.1128/AAC.00233-19 | NO | NO | PRECLINICAL STUDY |
| 2018 | S. Monsalvo, P. Balsalobre, N. D. Herrero, L. Solán, A. Fernandez-Cruz, M. Machado, M. D. Vigil-Escribano, J. Anguita, J. L. Díez-Martín and M. Kwon | Weekly screening for multidrug-resistant organisms identifies high number of colonizations in patients undergoing stem cell transplantation | 10.1182/blood-2018-99-118713 | NO | NO | OUT OF TOPIC |
| 2017 | A. Montero, P. S. Aranda, F. Gilsanz and E. Maseda | Antimicrobial management in nosocomial peritonitis: Microbiota, drug and time |  | NO | NO | REVIEW |
| 2020 | M. Montero, S. Domene Ochoa, C. Lopez-Causape, B. VanScoy, S. Luque, L. Sorli, N. Campillo, A. Angulo-Brunet, E. Padilla, N. Prim, V. Pomar, A. Rivera, S. Grau, P. G. Ambrose, A. Oliver and J. P. Horcajada | Efficacy of Ceftolozane-Tazobactam in Combination with Colistin against Extensively Drug-Resistant Pseudomonas aeruginosa, Including High-Risk Clones, in an In Vitro Pharmacodynamic Model | 10.1128/aac.02542-19 | NO | NO | IN VITRO STUDY |
| 2018 | M. Montero, B. D. VanScoy, C. Lopez-Causape, H. Conde, J. Adams, C. Segura, L. Zamorano, A. Oliver, J. P. Horcajada and P. G. Ambrose | Evaluation of Ceftolozane-Tazobactam in Combination with Meropenem against Pseudomonas aeruginosa Sequence Type 175 in a Hollow-Fiber Infection Model | 10.1128/aac.00026-18 | NO | NO | IN VITRO STUDY |
| 2019 | M. M. Montero, S. Domene Ochoa, C. Lopez-Causape, B. VanScoy, S. Luque, L. Sorli, N. Campillo, E. Padilla, N. Prim, C. Segura, V. Pomar, A. Rivera, S. Grau, P. G. Ambrose, A. Oliver and J. P. Horcajada | Colistin plus meropenem combination is synergistic in vitro against extensively drug-resistant Pseudomonas aeruginosa, including high-risk clones | 10.1016/j.jgar.2019.04.012 | NO | NO | IN VITRO STUDY |
| 2018 | J. Monterrubio-Villar, S. Rodriguez-Garrido and J. D. Jimenez-Delgado | Postoperative soft-tissue infection due to multidrug-resistant Pseudomonas aeruginosa: usefulness of ceftolozane-tazobactam |  | NO | NO | LETTER |
| 2018 | P. Montravers and M. Bassetti | The ideal patient profile for new beta-lactam/beta-lactamase inhibitors | 10.1097/QCO.0000000000000490 | NO | NO | REVIEW |
| 2019 | G. Montrucchio, G. Sales, S. Corcione, F. G. Derosa and L. Brazzi | Choosing wisely: What is the actual role of antimicrobial stewardship in Intensive Care Units? | 10.23736/S0375-9393.18.12662-9 | NO | NO | REVIEW |
| 2020 | R. E. Moore, B. C. Millar and J. E. Moore | Antimicrobial susceptibility of plastic-associated bacteria isolated from the ocean to novel antibiotics (delafloxacin, meropenem/vaborbactam, ceftolozane/tazobactam, ceftobiprole) - Can environmental bacteria be predictors of persistence of antibiotic activity in clinical pathogens? | 10.1016/j.ijheh.2020.113498 | NO | NO | COMMENT |
| 2019 | A. V. Morant, V. Jagalski and H. T. Vestergaard | Characteristics of Single Pivotal Trials Supporting Regulatory Approvals of Novel Non-orphan, Non-oncology Drugs in the European Union and United States from 2012−2016 | 10.1111/cts.12617 | NO | NO | PHARMACOLOGICAL STUDY |
| 2018 | M. S. Morehead and C. Scarbrough | Emergence of Global Antibiotic Resistance | 10.1016/j.pop.2018.05.006 | NO | NO | REVIEW |
| 2019 | I. Morrissey, S. Magnet, S. Hawser, S. Shapiro and P. Knechtle | In vitro activity of cefepime-enmetazobactam against Gram-negative isolates collected from U.S. And European hospitals during 2014–2015 | 10.1128/AAC.00514-19 | NO | NO | IN VITRO STUDY |
| 2018 | T. Moshiri, N. Mahida and S. Soo | Multi-resistant Gram negative Enterobactericeae in paediatrics: an infection prevention and control challenge | 10.1016/j.paed.2018.03.002 | NO | NO | REVIEW |
| 2018 | S. B. Mossad | Management of Infections in Solid Organ Transplant Recipients | 10.1016/j.idc.2018.06.001 | NO | NO | EDITORIAL |
| 2018 | A. Motos, J. M. Kidd and D. P. Nicolau | Optimizing Antibiotic Administration for Pneumonia | 10.1016/j.ccm.2018.08.006 | NO | NO | REVIEW |
| 2018 | A. Motos, J. L. Kuti, G. L. Bassi, A. Torres and D. P. Nicolau | β-lactam probability of target attainment (PTA) and penetration into epithelial lining fluid (ELF) based on multiple bronchoalveolar lavage (BAL) sampling time points in a swine pneumonia model | 10.1093/ofid/ofy210.1229 | NO | NO | PRECLINICAL STUDY |
| 2019 | A. Motos, J. L. Kuti, G. L. Bassi, A. Torres and D. P. Nicolaua | Is one sample enough? -lactam target attainment and penetration into epithelial lining fluid based on multiple bronchoalveolar lavage sampling time points in a swine pneumonia model | 10.1128/AAC.01922-18 | NO | NO | PRECLINICAL STUDY |
| 2012 | B. Moya, A. Beceiro, G. Cabot, C. Juan, L. Zamorano, S. Alberti and A. Oliver | Pan-beta-lactam resistance development in Pseudomonas aeruginosa clinical strains: molecular mechanisms, penicillin-binding protein profiles, and binding affinities | 10.1128/aac.00680-12 | NO | NO | PHARMACOLOGICAL STUDY |
| 2010 | B. Moya, L. Zamorano, C. Juan, Y. Ge and A. Oliver | Affinity of the new cephalosporin CXA-101 to penicillin-binding proteins of Pseudomonas aeruginosa | 10.1128/aac.00296-10 | NO | NO | PHARMACOLOGICAL STUDY |
| 2010 | B. Moya, L. Zamorano, C. Juan, J. L. Perez, Y. Ge and A. Oliver | Activity of a new cephalosporin, CXA-101 (FR264205), against beta-lactam-resistant Pseudomonas aeruginosa mutants selected in vitro and after antipseudomonal treatment of intensive care unit patients | 10.1128/aac.01104-09 | NO | NO | PHARMACOLOGICAL STUDY |
| 2019 | L. Mueller, A. Masseron, G. Prod’Hom, T. Galperine, G. Greub, L. Poirel and P. Nordmann | Phenotypic, biochemical, and genetic analysis of KPC-41, a KPC-3 variant conferring resistance to ceftazidime-avibactam and exhibiting reduced carbapenemase activity | 10.1128/AAC.01111-19 | NO | NO | CASE REPORT |
| 2019 | X. Mulet, R. Garcia, M. Gaya and A. Oliver | O-antigen serotyping and MALDI-TOF, potentially useful tools for optimizing semi-empiric antipseudomonal treatments through the early detection of high-risk clones | 10.1007/s10096-018-03457-z | NO | NO | IN VITRO STUDY |
| 2019 | E. M. Mullane, L. M. Avery and D. P. Nicolau | WCK 5222 (cefepime/zidebactam): an in vitro assessment of activity compared with current dual-antibiotic options against multidrug-resistant pseudomonas aeruginosa | 10.1093/ofid/ofz360.793 | NO | NO | IN VITRO STUDY |
| 2020 | E. M. Mullane, L. M. Avery and D. P. Nicolau | Comparative evaluation of the in vitro activities of WCK 5222 (Cefepime-Zidebactam) and combination antibiotic therapies against carbapenem-resistant Pseudomonas aeruginosa | 10.1128/AAC.01669-19 | NO | NO | IN VITRO STUDY |
| 2016 | J. Munita, S. Aitken, F. Perez, R. Rosa, R. Jain, M. Nigo, A. Wanger, R. Araos, T. Tran, F. P. Tverdek, L. Shimose, P. Lichtenberger, J. Adachi, L. Abbo, R. Rakita, S. Shelburne, R. A. Bonomo and C. Arias | Ceftolozane-tazobactam (C/T) for severe infections caused by carbapenem-resistant pseudomonas aeruginosa | 10.1093/ofid/ofw172.1584 | YES | NO | ABSTRACT |
| 2018 | S. Mushtaq, A. Vickers, Z. Payne, D. Kenna, J. F. Turton, N. Woodford and D. M. Livermore | Strain and antibiogram changes, over time, in paediatric cystic fibrosis patients colonised with Pseudomonas aeruginosa |  | NO | NO | IN VITRO STUDY |
| 2019 | K. G. Naber and F. M. E. Wagenlehner | Novel Antibiotics in the Treatment of Urinary Tract Infections | 10.1016/j.euf.2018.11.012 | NO | NO | REVIEW |
| 2019 | J. Naik, J. Yang, D. Elsea, S. Critchlow and L. Puzniak | Cost-effectiveness of ceftolozane/tazobactam for treating ventilated nosocomial bacterial pneumonia | 10.1093/ofid/ofz360.1880 | NO | NO | COST-EFFECTIVENESS ANALYSIS |
| 2019 | M. Nakajima, M. Kojiro, S. Aso, H. Matsui, K. Fushimi, Y. Kaita, H. Goto, Y. Yamaguchi and H. Yasunaga | Effect of high-dose vitamin C therapy on severe burn patients: a nationwide cohort study | 10.1186/s13054-019-2693-1 | NO | NO | OUT OF TOPIC |
| 2017 | P. Nambiar, K. Brizendine, V. Athans and E. Cober | Clinical experience with novel cephalosporin/beta-lactamase inhibitor combinations in the treatment of multidrug resistant pseudomonas aeruginosa and carbapenem-resistant enterobacteriaceae in solid organ transplant recipients | 10.1111/ajt.14306 | NO | NO | ABSTRACT |
| 2019 | P. H. Nambiar, K. D. Brizendine, V. Athans and E. Cober | Clinical experience with the use of novel cephalosporin/beta-lactamase inhibitor combinations among solid organ transplant recipients | 10.1111/tid.13161 | NO | NO | LETTER |
| 2016 | S. Natesan, M. P. Pai and T. P. Lodise | Determination of alternative ceftolozane/tazobactam (C/T) dosing regimens for patients with infections due to pseudomonas aeruginosa with high (C/T) mic values | 10.1093/ofid/ofw172.1507 | NO | NO | PHARMACOLOGICAL STUDY |
| 2017 | S. Natesan, M. P. Pai and T. P. Lodise | Determination of alternative ceftolozane/tazobactam dosing regimens for patients with infections due to Pseudomonas aeruginosa with MIC values between 4 and 32 mg/L | 10.1093/jac/dkx221 | NO | NO | PHARMACOLOGICAL STUDY |
| 2016 | R. V. Nathan, F. S. Alvarado, R. C. Prokesch, Q. Luu, T. K. Sleweon, C. P. Schroeder and L. J. Van Anglen | Ceftolozane/tazobactam: Outpatient treatment of gram-negative infections at physician office infusion centers (POICS) | 10.1093/ofid/ofw172.1603 | NO | NO | ABSTRACT |
| 2014 | D. Navarro, M. M. González Brao and M. Milà | Pipeline on the move | 10.1358/dof.2014.039.06.2172406 | NO | NO | REVIEW |
| 2013 | Nct | Study of Intravenous Ceftolozane/Tazobactam Compared to Piperacillin/Tazobactam in Ventilator-Associated Pneumonia |  | NO | NO | PROTOCL |
| 2014 | Nct | Safety and Efficacy Study of Ceftolozane/Tazobactam to Treat Ventilated Nosocomial Pneumonia (MK-7625A-008) |  | NO | NO | PROTOCL |
| 2018 | Nct | Comparative Study To Determine The Efficacy, Safety, And Tolerability Of Ceftolozane-Tazobactam |  | NO | NO | PROTOCL |
| 2018 | Nct | Comparison of Short Infusion Versus Prolonged Infusion of Ceftolozane-tazobactam Among Patients With Ventilator Associated-pneumonia to Pseudomonas Aeruginosa in Intensive Care Units |  | NO | NO | PROTOCL |
| 2019 | Nct | Ceftolozane/Tazobactam (MK-7625A) Plus Metronidazole Versus Meropenem for Participants With Complicated Intra-abdominal Infection (MK-7625A-015) |  | NO | NO | PROTOCL |
| 2020 | Nct | Ceftolozane-tazobactam Versus Meropenem for ESBL and AmpC-producing Enterobacterales Bloodstream Infection |  | NO | NO | REVIEW |
| 2019 | C. P. Nguyen, T. N. Dan Do, R. Bruggemann, J. ten Oever, E. Kolwijck, E. M. M. Adang and H. F. L. Wertheim | Clinical cure rate and cost-effectiveness of carbapenem-sparing beta-lactams vs. meropenem for Gram-negative infections: A systematic review, meta-analysis, and cost-effectiveness analysis | 10.1016/j.ijantimicag.2019.07.003 | NO | NO | COST-EFFECTIVENESS ANALYSIS |
| 2018 | L. Nguyen, J. Garcia, K. Gruenberg and C. MacDougall | Multidrug-Resistant Pseudomonas Infections: Hard to Treat, But Hope on the Horizon? | 10.1007/s11908-018-0629-6 | NO | NO | REVIEW |
| 2016 | A. M. Nicasio, B. D. Van Scoy, R. E. Mendes, M. Castanheira, C. C. Bulik, O. O. Okusanya, S. M. Bhavnani, A. Forrest, R. N. Jones, L. V. Friedrich, J. N. Steenbergen and P. G. Ambrose | Pharmacokinetics-pharmacodynamics of tazobactam in combination with piperacillin in an in vitro infection model | 10.1128/AAC.02747-15 | NO | NO | PHARMACOLOGICAL STUDY |
| 2019 | D. Nichol, J. Rutter, C. Bryant, A. M. Hujer, S. Lek, M. D. Adams, P. Jeavons, A. R. A. Anderson, R. A. Bonomo and J. G. Scott | Antibiotic collateral sensitivity is contingent on the repeatability of evolution | 10.1038/s41467-018-08098-6 | NO | NO | OUT OF TOPIC |
| 2018 | M. H. Nicolas-Chanoine, N. Mayer, K. Guyot, E. Dumont and J. M. Pages | Interplay Between Membrane Permeability and Enzymatic Barrier Leads to Antibiotic-Dependent Resistance in Klebsiella Pneumoniae | 10.3389/fmicb.2018.01422 | NO | NO | IN VITRO STUDY |
| 2015 | D. P. Nicolau | Focus on ceftazidime-avibactam for optimizing outcomes in complicated intra-abdominal and urinary tract infections | 10.1517/13543784.2015.1062873 | NO | NO | REVIEW |
| 2018 | X. M. Nie, Y. S. Li, Z. W. Yang, H. Wang, S. Y. Jin, Y. Jiao, M. L. Metersky and Y. Huang | Initial empiric antibiotic therapy for community-acquired pneumonia in Chinese hospitals | 10.1016/j.cmi.2017.09.014 | NO | NO | ABSTRACT |
| 2017 | M. S. Niederman, I. Martin-Loeches and A. Torres | The research agenda in VAP/HAP: next steps | 10.1007/s00134-017-4695-2 | NO | NO | REVIEW |
| 2018 | E. S. Niranjana, R. Sambath Kumar, M. Sudha and N. Venkateswaramurthy | Review on clinically developing antibiotics | 10.22159/ijap.2018v10i3.22668 | NO | NO | REVIEW |
| 2018 | A. R. Noel, K. E. Bowker, M. Attwood and A. P. MacGowan | Antibacterial effect of ceftolozane/tazobactam in combination with amikacin against aerobic Gram-negative bacilli studied in an in vitro pharmacokinetic model of infection | 10.1093/jac/dky225 | NO | NO | IN VITRO STUDY |
| 2019 | P. J. Nolan, T. Smith, J. D. Finklea, L. Cohen and R. Jain | Ceftolozane-tazobactam demonstrates higher in vitro susceptibility than ceftazidime-avibactam against pseudomonas aeruginosa isolated from respiratory tract of adult cystic fibrosis patients | 10.1093/ofid/ofz360.1458 | NO | NO | IN VITRO STUDY |
| 2018 | A. Novelli | Resistenze antibiotiche e nuove molecole: qual è lo scenario attuale? | 10.1177/0391560318773251 | NO | NO | REVIEW |
| 2018 | S. Nseir and I. Martin-Loeches | Hot topics in ventilator-associated pneumonia | 10.21037/atm.2018.11.09 | NO | NO | REVIEW |
| 2020 | A. K. Nussbaumer-Proll, S. Eberl, B. Reiter, T. Stimpfl, C. Dorn and M. Zeitlinger | Low pH reduces the activity of ceftolozane/tazobactam in human urine, but confirms current breakpoints for urinary tract infections | 10.1093/jac/dkz488 | NO | NO | IN VITRO STUDY |
| 2020 | H. Oberpichler-Schwenk | Nosocomial infections: Ceftolozane/tazobactam for nosocomial pneumonia |  | NO | NO | REVIEW |
| 2019 | J. Odland | IDWeek 2019. Washington, D.C., USA-October 2-6, 2019 | 10.1358/dof.2019.44.11.3084078 | NO | NO | REVIEW |
| 2017 | A. Oesch, R. Dhami, C. Lougheed and S. Elsayed | Thrombocytopenia during treatment with ceftolozane/tazobactam |  | NO | NO | CASE REPORT |
| 2018 | T. Olarte-Luis, D. Caceres-Galindez and J. A. Cortes | [New cephalosporins] | 10.4067/s0716-10182018000500465 | NO | NO | REVIEW |
| 2010 | A. Oliver | Mutators in cystic fibrosis chronic lung infection: Prevalence, mechanisms, and consequences for antimicrobial therapy | 10.1016/j.ijmm.2010.08.009 | NO | NO | REVIEW |
| 2015 | W. Oliver, J. Gonzales, E. Heil, S. Mehrotra, K. Robinett, P. Saleeb and D. Nicolau | Ceftolozane-tazobactam pharmacokinetics in a patient on continuous veno-venous hemofiltration | 10.1097/01.ccm.0000475030.89724.fe | NO | NO | CASE REPORT |
| 2016 | W. D. Oliver, E. L. Heil, J. P. Gonzales, S. Mehrotr, K. Robinett, P. Salee and D. P. Nicolau | Ceftolozane-tazobactam pharmacokinetics in a critically ill patient on continuous venovenous hemofiltration | 10.1128/AAC.02608-15 | NO | NO | PHARMACOLOGICAL STUDY |
| 2020 | D. O'Neall, E. Juhasz, A. Toth, E. Urban, J. Szabo, S. Melegh, K. Katona and K. Kristof | Ceftazidime-avibactam and ceftolozane-tazobactam susceptibility of multidrug resistant Pseudomonas aeruginosa strains in Hungary | 10.1556/030.2020.01152 | NO | NO | IN VITRO STUDY |
| 2008 | A. J. O'Neill | New antibacterial agents for treating infections caused by multi-drug resistant gram-negative bacteria | 10.1517/13543784.17.3.297 | NO | NO | REVIEW |
| 2019 | J. M. Ortiz De La Rosa, P. Nordmann and L. Poirel | ESBLs and resistance to ceftazidime/avibactam and ceftolozane/tazobactam combinations in Escherichia coli and Pseudomonas aeruginosa | 10.1093/jac/dkz149 | NO | NO | IN VITRO STUDY |
| 2015 | K. Outterson, J. H. Powers, G. W. Daniel and M. B. McClellan | Repairing the broken market for antibiotic innovation | 10.1377/hlthaff.2014.1003 | NO | NO | REVIEW |
| 2014 | M. G. Page and K. Bush | Discovery and development of new antibacterial agents targeting Gram-negative bacteria in the era of pandrug resistance: Is the future promising? | 10.1016/j.coph.2014.09.008 | NO | NO | REVIEW |
| 2009 | M. G. Page and J. Heim | Prospects for the next anti-Pseudomonas drug | 10.1016/j.coph.2009.08.006 | NO | NO | REVIEW |
| 2012 | M. G. P. Page | The role of the outer membrane of gram-negative bacteria in antibiotic resistance: Ajax' shield or achilles' heel? | 10.1007/978-3-642-28951-4-5 | NO | NO | REVIEW |
| 2009 | M. G. P. Page and J. Heim | New molecules from old classes: Revisiting the development of β-lactams |  | NO | NO | REVIEW |
| 2018 | Z. D. Pana and T. Zaoutis | Treatment of extended-spectrum β-lactamase-producing enterobacteriaceae (ESBLS) infections: What have we learned until now? [version 1; referees: 2 approved] | 10.12688/f1000research.14822.1 | NO | NO | REVIEW |
| 2019 | K. M. Papp-Wallace | The latest advances in beta-lactam/beta-lactamase inhibitor combinations for the treatment of Gram-negative bacterial infections | 10.1080/14656566.2019.1660772 | NO | NO | REVIEW |
| 2016 | K. M. Papp-Wallace and R. A. Bonomo | New β-Lactamase Inhibitors in the Clinic | 10.1016/j.idc.2016.02.007 | NO | NO | REVIEW |
| 2018 | L. Papst, B. Beović, C. Pulcini, E. Durante-Mangoni, J. Rodríguez-Baño, K. S. Kaye, G. L. Daikos, L. Raka, M. Paul, L. Abbo, P. Abgueguen, B. Almirante, A. M. Azzini, F. Bani-Sadr, M. Bassetti, R. Ben-Ami, B. Beović, G. Béraud, E. Botelho-Nevers, G. Bou, D. Boutoille, A. Cabié, B. Cacopardo, A. Cascio, N. Cassir, F. Castelli, M. Cecala, A. Charmillon, C. Chirouze, J. M. Cisneros, J. D. Colmenero, N. Coppola, S. Corcione, G. L. Daikos, D. Dalla Gasperina, C. De la Calle Cabrera, P. Delobel, D. Di Caprio, E. Durante Mangoni, M. Dupon, N. Ettahar, M. E. Falagas, M. Falcone, M. C. Fariñas, E. Faure, E. Forestier, G. Foti, J. Gallagher, G. Gattuso, V. Gendrin, I. Gentile, D. R. Giacobbe, C. A. Gogos, L. Grandiere Perez, Y. Hansmann, J. P. Horcajada, C. Iacobello, J. T. Jacob, J. A. Justo, S. Kernéis, A. Komnos, B. Kotnik Kevorkijan, D. Lebeaux, R. Le Berre, C. Lechiche, V. Le Moxing, F. X. Lescure, M. Libanore, M. Martinot, E. Merino de Lucas, V. Mondain, P. Mondello, M. Montejo, J. Mootien, P. Muñoz, R. Nir-Paz, A. Pan, J. R. Paño-Pardo, G. Patel, M. Paul, M. T. Pérez Rodríguez, L. Piroth, J. Pogue, B. A. Potoski, V. Pourcher, A. Pyrpasopoulou, G. Rahav, M. Rizzi, J. Rodríguez-Baño, M. Salavert, M. Scheetz, M. Sims, G. Spahija, S. Stefani, A. Stefos, P. D. Tamma, P. Tattevin, A. Tedesco, J. Torre-Cisneros, P. Tripolitsioti, S. Tsiodras, G. Uomo, R. Verdon, P. Viale, V. Vitrat, M. Weinberger and Y. Wiener-Well | Antibiotic treatment of infections caused by carbapenem-resistant Gram-negative bacilli: an international ESCMID cross-sectional survey among infectious diseases specialists practicing in large hospitals | 10.1016/j.cmi.2018.01.015 | NO | NO | OUT OF TOPIC |
| 2019 | S. C. Park, A. M. Wailan, K. E. Barry, K. Vegesana, J. Carroll, A. J. Mathers, W. R. Miller and J. M. Munita | Managing all the genotypic knowledge: Approach to a septic patient colonized by different Enterobacteriales with unique carbapenemases | 10.1128/AAC.00029-19 | NO | NO | CASE REPORT |
| 2019 | T. S. Patel, K. S. Kaye, V. Marshall, J. Krishnan, J. Mills, O. Albin, A. Smith, C. Young, P. Lephart and J. M. Pogue | In vitro antimicrobial activity of ceftazidime/avibactam compared with ceftolozane/tazobactam against real-world clinical isolates of pseudomonas aeruginosa at a large academic tertiary care hospital | 10.1093/ofid/ofz360.591 | NO | NO | IN VITRO STUDY |
| 2019 | T. S. Patel, V. Marshall, K. S. Kaye, A. Smith, C. Young, P. Lephart and J. M. Pogue | Susceptibility of β-lactam-resistant pseudomonas aeruginosa to other β-lactams: is there truly a lack of cross-resistance? | 10.1093/ofid/ofz360.1464 | NO | NO | IN VITRO STUDY |
| 2016 | U. C. Patel, D. P. Nicolau and R. K. Sabzwari | Successful Treatment of Multi-Drug Resistant Pseudomonas aeruginosa Bacteremia with the Recommended Renally Adjusted Ceftolozane/Tazobactam Regimen | 10.1007/s40121-016-0104-3 | NO | NO | CASE REPORT |
| 2015 | D. L. Paterson and P. N. A. Harris | Editorial Commentary: The New Acinetobacter Equation: Hypervirulence Plus Antibiotic Resistance Equals Big Trouble | 10.1093/cid/civ227 | NO | NO | EDITORIAL |
| 2017 | D. L. Paterson and D. van Duin | China's antibiotic resistance problems | 10.1016/S1473-3099(17)30053-1 | NO | NO | COMMENT |
| 2019 | C. Pazzini, P. Ahmad-Nejad and B. Ghebremedhin | Ceftolozane/tazobactam susceptibility testing in extended-spectrum betalactamase- and carbapenemase-producing gram-negative bacteria of various clonal lineages | 10.1556/1886.2019.00001 | NO | NO | IN VITRO STUDY |
| 2017 | M. Peetermans, J. Verhaegen, W. Peetermans and J. Wauters | Empirical antibiotic therapy in the face of rising ESBL incidence | 10.2143/TVG.73.13.2002370 | NO | NO | REVIEW |
| 2018 | M. Peghin, M. Maiani, N. Castaldo, F. Givone, E. Righi, A. Lechiancole, A. Sartor, F. Pea, U. Livi and M. Bassetti | Ceftolozane/tazobactam for the treatment of MDR Pseudomonas aeruginosa left ventricular assist device infection as a bridge to heart transplant | 10.1007/s15010-017-1086-0 | NO | NO | CASE REPORT |
| 2017 | L. H. Pelzl, T. Hoppe-Tichy and M. Karck | Ceftolozane/tazobactam in long-term therapy: Creatine kinase elevation as possible adverse drug effect | <http://www.embase.com/search/results?subaction=viewrecord&from=export&id=L616102879> | NO | NO | NOT IN ENGLISH |
| 2019 | A. Perez, E. Gato, J. Perez-Llarena, F. Fernandez-Cuenca, M. J. Gude, M. Oviano, M. E. Pachon, J. Garnacho, V. Gonzalez, A. Pascual, J. M. Cisneros and G. Bou | High incidence of MDR and XDR Pseudomonas aeruginosa isolates obtained from patients with ventilator-associated pneumonia in Greece, Italy and Spain as part of the MagicBullet clinical trial | 10.1093/jac/dkz030 | NO | NO | IN VITRO STUDY |
| 2015 | F. Perez and R. A. Bonomo | Editorial commentary: Bloodstream infection caused by extended-spectrum β-lactamase-producing gram-negative bacteria: How to define the best treatment regimen? | 10.1093/cid/civ007 | NO | NO | EDITORIAL |
| 2018 | F. Perez and R. A. Bonomo | Evidence to improve the treatment of infections caused by carbapenem-resistant Gram-negative bacteria | 10.1016/S1473-3099(18)30112-9 | NO | NO | COMMENT |
| 2019 | L. R. R. Perez | The impact of efflux pumps on meropenem susceptibility among metallo-β-lactamase-producing and nonproducing Pseudomonas aeruginosa: Insights for better antimicrobial stewardship | 10.1017/ice.2019.151 | NO | NO | LETTER |
| 2020 | L. R. R. Perez, E. Carniel and G. A. Narvaez | High minimum inhibitory concentrations among derepressed AmpC-beta-lactamase-producing Enterobacter cloacae complex isolates for ceftolozane with tazobactam | 10.1017/ice.2020.31 | NO | NO | LETTER |
| 2019 | A. M. Peri, Y. Doi, B. A. Potoski, P. N. A. Harris, D. L. Paterson and E. Righi | Antimicrobial treatment challenges in the era of carbapenem resistance | 10.1016/j.diagmicrobio.2019.01.020 | NO | NO | REVIEW |
| 2019 | F. Perier, S. Couffin, M. Martin, J. Bardon, F. Cook and R. Mounier | Multidrug-Resistant Acinetobacter baumannii Ventriculostomy-Related Infection, Treated by a Colistin, Tigecycline, and Intraventricular Fibrinolysis | 10.1016/j.wneu.2018.09.218 | NO | NO | CASE REPORT |
| 2010 | G. Perletti, V. Magri, F. M. E. Wagenlehner and K. G. Naber | CXA-101: Cephalosporin antibiotic | 10.1358/dof.2010.035.012.1541551 | NO | NO | REVIEW |
| 2019 | N. Pervin, K. B. Tate-Nero, S. Ullah, S. Koirala, V. Sundareshan and J. Koirala | Comparison of clinical outcomes in patients with extensively drug-resistant pseudomonas aeruginosa pneumonia treated with aminoglycosides vs. ceftolozane/tazobactam | 10.1093/ofid/ofz360.1952 | YES | NO | ABSTRACT |
| 2019 | V. Petraitis, R. Petraitiene, E. Naing, T. Aung, W. P. Thi, P. Kavaliauskas, B. B. W. Maung, A. O. Michel, R. J. Ricart Arbona, A. C. DeRyke, D. L. Culshaw, D. P. Nicolau, M. J. Satlin and T. J. Walsh | Ceftolozane-Tazobactam in the Treatment of Experimental Pseudomonas aeruginosa Pneumonia in Persistently Neutropenic Rabbits: Impact on strains with genetically defined mechanisms of resistance | 10.1128/AAC.00344-19 | NO | NO | PRECLINICAL STUDY |
| 2020 | N. Petrosillo, G. Granata, B. Boyle, M. M. Doyle, B. Pinchera and F. Taglietti | Preventing sepsis development in complicated urinary tract infections | 10.1080/14787210.2020.1700794 | NO | NO | REVIEW |
| 2019 | M. Pezzi, A. M. Scozzafava, A. M. Giglio, R. Vozzo, P. D. Casella, S. P. Tiburzi, L. Cosco and M. Verre | Use of Ceftolozane/Tazobactam in a Case of Septic Shock by Puerperal Sepsis | 10.1155/2019/8463693 | NO | NO | CASE REPORT |
| 2017 | M. A. Pfaller, M. Bassetti, L. R. Duncan and M. Castanheira | Ceftolozane/tazobactam activity against drug-resistant Enterobacteriaceae and Pseudomonas aeruginosa causing urinary tract and intraabdominal infections in Europe: Report from an antimicrobial surveillance programme (2012-15) | 10.1093/jac/dkx009 | NO | NO | IN VITRO STUDY |
| 2018 | M. A. Pfaller, D. Shortridge, H. S. Sader, M. Castanheira and R. K. Flamm | Ceftolozane/tazobactam activity against drug-resistant Enterobacteriaceae and Pseudomonas aeruginosa causing healthcare-associated infections in the Asia-Pacific region (minus China, Australia and New Zealand): report from an Antimicrobial Surveillance Programme (2013–2015) | 10.1016/j.ijantimicag.2017.09.016 | NO | NO | IN VITRO STUDY |
| 2017 | M. A. Pfaller, D. Shortridge, H. S. Sader, R. K. Flamm and M. Castanheira | Ceftolozane–tazobactam activity against drug-resistant Enterobacteriaceae and Pseudomonas aeruginosa causing healthcare-associated infections in Australia and New Zealand: Report from an Antimicrobial Surveillance Program (2013–2015) | 10.1016/j.jgar.2017.05.025 | NO | NO | IN VITRO STUDY |
| 2017 | M. A. Pfaller, D. Shortridge, H. S. Sader, A. Gales, M. Castanheira and R. K. Flamm | Ceftolozane-tazobactam activity against drug-resistant Enterobacteriaceae and Pseudomonas aeruginosa causing healthcare-associated infections in Latin America: report from an antimicrobial surveillance program (2013–2015) | 10.1016/j.bjid.2017.06.008 | NO | NO | IN VITRO STUDY |
| 2019 | V. D. Pilato, A. Antonelli, T. Giani, L. H. De Angelis, G. M. Rossolini and S. Pollini | Identification of a novel plasmid lineage associated with the dissemination of metallo-β-lactamase genes among pseudomonads | 10.3389/fmicb.2019.01504 | NO | NO | IN VITRO STUDY |
| 2019 | B. Pilmis, G. Petitjean, P. Lesprit, M. Lafaurie, N. El Helali and A. Le Monnier | Continuous infusion of ceftolozane/tazobactam is associated with a higher probability of target attainment in patients infected with Pseudomonas aeruginosa | 10.1007/s10096-019-03573-4 | NO | NO | PHARMACOLOGICAL STUDY |
| 2014 | P. Pina | 24<sup>th</sup> European Congress of Clinical Microbiology and Infectious Diseases (ECCMID) Barcelona, Spain - May 10-13, 2014 | 10.1358/dof.2014.039.06.2170496 | NO | NO | CONFERENCE PROCEEDINGS |
| 2018 | A. J. Plant, A. Dunn and R. J. Porter | Ceftolozane-tazobactam resistance induced in vivo during the treatment of MDR Pseudomonas aeruginosa pneumonia | 10.1080/14787210.2018.1473079 | YES | NO | LETTER |
| 2018 | T. Plusa | [New antibiotics in infection treatment] |  | NO | NO | REVIEW |
| 2015 | J. M. Pogue, D. A. Cohen and D. Marchaim | Editorial commentary: Polymyxin-resistant acinetobacter baumannii: Urgent action needed | 10.1093/cid/civ044 | NO | NO | EDITORIAL |
| 2018 | J. M. Pogue, K. S. Kaye, M. Veve, A. Gerlach, T. S. Patel, S. L. Davis, E. Chaung, A. Ray, L. Puzniak, R. A. Bonomo and F. Perez | “Real-world” treatment of multidrug-resistant (MDR) or Extensively Drug-Resistant (XDR) P. Aeruginosa Infections With Ceftolozane/Tazobactam (C/T) vs. A Polymyxin or Aminoglycoside (Poly/AG)-based Regimen: A multicenter comparative effectiveness study | 10.1093/ofid/ofy210.2059 | YES | NO | ABSTRACT |
| 2017 | J. M. Pogue, L. Puzniak, S. Merchant, R. Sanagaram and E. Rhee | Real-world analysis of prescribing patterns and susceptibility of ceftolozane/tazobactam (c/t) treatment using an electronic medical record (emr) database in the united states | 10.1093/ofid/ofx163.651 | NO | NO | ABSTRACT |
| 2019 | L. Poirel, J. M. O. De La Rosa, N. Kieffer, V. Dubois, A. Jayol and P. Nordmanna | Acquisition of extended-spectrum -lactamase ges-6 leading to resistance to ceftolozane-tazobactam combination in Pseudomonas Aeruginosa | 10.1128/AAC.01809-18 | NO | NO | IN VITRO STUDY |
| 2017 | J. Poissy, E. Parmentier-Decrucq, C. Thieffry, T. Duburcq and D. Mathieu | “New” drugs for infectious diseases: Can ceftolozane–tazobactam and temocillin be used in intensive care | 10.1007/s13546-017-1267-y | NO | NO | REVIEW |
| 2015 | C. Popa, M. Mircheva, B. K. Kramer, A. Berghofen and B. Kruger | Ceftolozane-tazobactam versus levofloxacin in urinary tract infection | 10.1016/s0140-6736(15)00262-7 | YES | NO | LETTER |
| 2017 | M. W. Popejoy, J. Long and J. A. Huntington | Analysis of patients with diabetes and complicated intra-abdominal infection or complicated urinary tract infection in phase 3 trials of ceftolozane/tazobactam | 10.1186/s12879-017-2414-9 | NO | NO | OUT OF TOPIC |
| 2017 | M. W. Popejoy, D. L. Paterson, D. Cloutier, J. A. Huntington, B. Miller, C. A. Bliss, J. N. Steenbergen, E. Hershberger, O. Umeh and K. S. Kaye | Efficacy of ceftolozane/tazobactam against urinary tract and intra-abdominal infections caused by ESBL-producing Escherichia coli and Klebsiella pneumoniae: a pooled analysis of Phase 3 clinical trials | 10.1093/jac/dkw374 | NO | NO | NOT COMPARABLE DATA |
| 2014 | A. Pop-Vicas and S. M. Opal | The clinical impact of multidrug-resistant gram-negative bacilli in the management of septic shock | 10.4161/viru.26210 | NO | NO | REVIEW |
| 2019 | A. Potron, D. Fournier, C. Emeraud, P. Triponney, P. Plésiat, T. Naas and L. Dortet | Evaluation of the immunochromatographic NG-test CARbA 5 for rapid identification of carbapenemase in nonfermenters | 10.1128/AAC.00968-19 | NO | NO | IN VITRO STUDY |
| 2014 | G. Poulakou, M. Bassetti, E. Righi and G. Dimopoulos | Current and future treatment options for infections caused by multidrug-resistant Gram-negative pathogens | 10.2217/fmb.14.58 | NO | NO | REVIEW |
| 2018 | G. Poulakou, S. Lagou, D. E. Karageorgopoulos and G. Dimopoulos | New treatments of multidrug-resistant Gram-negative ventilator-associated pneumonia | 10.21037/atm.2018.10.29 | NO | NO | REVIEW |
| 2017 | V. Prabhu, J. Foo, H. Ahir, E. Sarpong and S. Merchant | Cost-effectiveness of ceftolozane/tazobactam plus metronidazole compared with piperacillin/tazobactam as empiric therapy for the treatment of complicated intra-abdominal infections based on the in-vitro surveillance of bacterial isolates in the UK | 10.1080/13696998.2017.1333960 | NO | NO | COST-EFFECTIVENESS ANALYSIS |
| 2015 | V. Prabhu, S. Sen, B. Miller, A. Basu and G. Medic | Using an economic model to choose initial appropriate antibiotic therapy based on differences in in-vitro susceptibility to ceftolozane/tazobactam and piperacillin/tazobactam |  | NO | NO | COST-EFFECTIVENESS ANALYSIS |
| 2017 | V. S. Prabhu, J. S. Solomkin, G. Medic, J. Foo, R. H. Borse, T. Kauf, B. Miller, S. S. Sen and A. Basu | Cost-effectiveness of ceftolozane/tazobactam plus metronidazole versus piperacillin/tazobactam as initial empiric therapy for the treatment of complicated intra-abdominal infections based on pathogen distributions drawn from national surveillance data in the United States | 10.1186/s13756-017-0264-2 | NO | NO | COST-EFFECTIVENESS ANALYSIS |
| 2018 | R. Pradhan and S. Singh | Comparison of Data on Serious Adverse Events and Mortality in ClinicalTrials.gov, Corresponding Journal Articles, and FDA Medical Reviews: Cross-Sectional Analysis | 10.1007/s40264-018-0666-y | NO | NO | OUT OF TOPIC |
| 2019 | C. D. Prado-Montoro, J. Ruiz-Aragon, C. Martinez Rubio, S. Garcia-Martin, C. Freyre-Carrillo and M. A. Rodriguez-Iglesias | [Evaluation of susceptibility of multidrug-resistant Pseudomonas aeruginosa strains against ceftolozane/tazobactam] | 10.4067/s0716-10182019000500551 | NO | NO | IN VITRO STUDY |
| 2018 | A. Pragasam, B. Veeraraghavan, E. Nalini, S. Anandan and K. Kaye | An update on antimicrobial resistance and the role of newer antimicrobial agents for Pseudomonas aeruginosa | 10.4103/ijmm.IJMM_18_334 | NO | NO | REVIEW |
| 2019 | A. K. Pragasam, B. Veeraraghavan, B. Shankar, Y. Bakthavatchalam, A. Mathuram, B. George, B. Chacko, P. Korula and S. Anandan | Will ceftazidime/avibactam plus aztreonam be effective for NDM and OXA-48-Like producing organisms: Lessons learnt from in vitro study | 10.4103/ijmm.IJMM_19_189 | NO | NO | IN VITRO STUDY |
| 2018 | A. K. Pragsam, D. T. Kumar, C. G. P. Doss, R. Iyadurai, S. Satyendra, C. Rodrigues, S. Joshi, I. Roy, B. N. Chaudhuri, D. S. Chitnis, D. Tapan and B. Veeraraghavan | In silico and in vitro activity of ceftolozane/tazobactam against pseudomonas aeruginosa collected across Indian hospitals | 10.4103/ijmm.IJMM_17_349 | NO | NO | IN VITRO STUDY |
| 2019 | R. Prevel, A. Boyer, F. M'Zali, T. Cockenpot, A. Lasheras, V. Dubois and D. Gruson | Extended spectrum beta-lactamase producing Enterobacterales faecal carriage in a medical intensive care unit: Low rates of cross-transmission and infection | 10.1186/s13756-019-0572-9 | NO | NO | IN VITRO STUDY |
| 2018 | L. Principe, A. Piazza, C. Mauri, A. Anesi, S. Bracco, G. Brigante, E. Casari, C. Agrappi, M. Caltagirone, F. Novazzi, R. Migliavacca, L. Pagani and F. Luzzaro | Multicenter prospective study on the prevalence of colistin resistance in escherichia coli: Relevance of mcr-1-positive clinical isolates in Lombardy, Northern Italy | 10.2147/IDR.S160489 | NO | NO | IN VITRO STUDY |
| 2018 | M. Probst-Kepper and G. Geginat | New Antibiotics for Treatment of Highly Resistant Gram-negative Bacteria | 10.1055/s-0043-110504 | NO | NO | REVIEW |
| 2017 | A. Pulido-Cejudo, M. Guzmán-Gutierrez, A. Jalife-Montaño, A. Ortiz-Covarrubias, J. L. Martínez-Ordaz, H. F. Noyola-Villalobos and L. M. Hurtado-López | Management of acute bacterial skin and skin structure infections with a focus on patients at high risk of treatment failure | 10.1177/2049936117723228 | NO | NO | REVIEW |
| 2019 | S. Purohit and S. Hughes | Reducing cephalosporin-related supply errors | 10.1211/CP.2019.20206444 | NO | NO | LETTER |
| 2019 | L. Puzniak, R. Fu, J. Gundrum and T. Lodise | Real-world evaluation of ceftolozane/ tazobactam treatment for pseudomonas across 253 U.S. hospitals |  | NO | NO | ABSTRACT |
| 2018 | L. Puzniak, R. Fu, J. Gundrum and T. P. Lodise | Real-world evaluation of patient characteristics and outcomes of patients treated with ceftolozane/tazobactam across 253 us hospitals | 10.1093/ofid/ofy210.2067 | NO | NO | ABSTRACT |
| 2019 | C. A. Pybus and D. E. Greenberg | Cefiderocol retains anti-biofilm activity in mdr gram-negative pathogens | 10.1093/ofid/ofz360.787 | NO | NO | ABSTRACT |
| 2014 | W. Qin, M. Panunzio and S. Biondi | β-Lactam antibiotics renaissance | 10.3390/antibiotics3020193 | NO | NO | REVIEW |
| 2019 | A. N. Quan, M. T. Zmarlicka, M. Matthews, D. Buchanan, K. J. Richey and K. N. Foster | Review of ceftolozane-tazobactam (C/T) use in burn patients | 10.1093/jbcr/irz013.282 | NO | NO | REVIEW |
| 2019 | E. Raby, S. Naicker, F. B. Sime, L. Manning, S. C. Wallis, S. Pandey and J. A. Roberts | Ceftolozane-tazobactam in an elastomeric infusion device for ambulatory care: An in vitro stability study | 10.1136/ejhpharm-2019-002093 | NO | NO | IN VITRO STUDY |
| 2017 | H. Rac, K. R. Stover, J. L. Wagner, S. T. King, H. D. Warnock and K. E. Barber | Time–Kill Analysis of Ceftolozane/Tazobactam Efficacy Against Mucoid Pseudomonas aeruginosa Strains from Cystic Fibrosis Patients | 10.1007/s40121-017-0176-8 | NO | NO | IN VITRO STUDY |
| 2017 | J. Rademacher and T. Welte | New antibiotics – standstill or progress | 10.1007/s00063-017-0271-3 | NO | NO | REVIEW |
| 2013 | C. Rafat, I. Debrix and A. Hertig | Levofloxacin for the treatment of pyelonephritis | 10.1517/14656566.2013.792805 | NO | NO | REVIEW |
| 2020 | M. Rahimzadeh, M. Habibi, S. Bouzari and M. R. Asadi Karam | First Study of Antimicrobial Activity of Ceftazidime-Avibactam and Ceftolozane-Tazobactam Against Pseudomonas aeruginosa Isolated from Patients with Urinary Tract Infection in Tehran, Iran | 10.2147/idr.S243301 | NO | NO | IN VITRO STUDY |
| 2017 | S. Rajan, D. Verotta, C. Sutherland, D. P. Nicolau, A. Spormann and K. Yang | Spatio-temporal activity of human-simulated exposures of meropenem (MEM) and ceftolozane (TOL) on pseudomonas aeruginosa biofilms | 10.1093/ofid/ofx163.673 | NO | NO | IN VITRO STUDY |
| 2020 | M. Rajaofera, X. Kang, P. F. Jin, X. Chen, C. C. Li, L. Yin, L. Liu, Q. H. Sun, N. Zhang, C. Z. Chen, N. He, Q. F. Xia and W. G. Miao | Antibacterial activity of bacillomycin D-like compounds isolated from Bacillus amyloliquefaciens HAB-2 against Burkholderia pseudomallei | 10.4103/2221-1691.280295 | NO | NO | IN VITRO STUDY |
| 2016 | S. Ramírez-Estrada, B. Borgatta and J. Rello | Pseudomonas aeruginosa ventilator-associated pneumonia management | 10.2147/IDR.S50669 | NO | NO | REVIEW |
| 2019 | S. Ranjitkar, A. K. Jones, M. Mostafavi, Z. Zwirko, O. Iartchouk, S. Whitney Barnes, J. R. Walker, T. W. Willis, P. S. Lee and C. R. Dean | Target (MexB)- and Efflux-Based Mechanisms Decreasing the Effectiveness of the Efflux Pump Inhibitor D13-9001 in Pseudomonas aeruginosa PAO1: Uncovering a new role for MexMn-OpRM in efflux of -lactams and a novel regulatory circuit (MMNRS) controlling MexMn expression | 10.1128/AAC.01718-18 | NO | NO | IN VITRO STUDY |
| 2020 | E. Ransom, A. Bhatnagar, J. B. Patel, M. J. Machado, S. Boyd, N. Reese, J. D. Lutgring, D. Lonsway, K. Anderson, A. C. Brown, C. A. Elkins, J. Kamile Rasheed and M. Karlsson | Validation of aztreonam-avibactam susceptibility testing using digitally dispensed custom panels | 10.1128/JCM.01944-19 | NO | NO | IN VITRO STUDY |
| 2016 | G. G. Rao, N. S. Ly, B. T. Tsuji, J. B. Bulitta and A. Forrest | Translational modeling of antibacterial agents | 10.1007/978-3-319-44534-2_17 | NO | NO | BOOK |
| 2019 | S. N. Rao, S. T. Nguyen, M. M. Soriano, J. M. Hayes, M. M. Hackel, D. F. Sahm, G. S. Tillotson, R. Echols, M. Tsuji and Y. Yamano | In vitro antibacterial activity of cefiderocol against a multi-national collection of carbapenem-nonsusceptible gram-negative bacteria from respiratory infections: Sidero-wt-2014-2017 | 10.1093/ofid/ofz360.760 | NO | NO | IN VITRO STUDY |
| 2016 | G. Ravasio, D. Crocchiolo and M. A. Veneziano | Cost effectiveness analysis of ceftolozane-tazobactam in complicated urinary tract infections (CUTI) and complicated intra-abdominal infections (CIAI) sustained by gram negative pathogens | 10.5301/GRHTA-ITJ.2016.15917 | NO | NO | COST-EFFECTIVENESS ANALYSIS |
| 2018 | M. Rawlins, V. Cheng, E. Raby, J. Dyer, A. Regli, P. Ingram, B. C. McWhinney, J. P. J. Ungerer and J. A. Roberts | Pharmacokinetics of Ceftolozane-Tazobactam during Prolonged Intermittent Renal Replacement Therapy | 10.1159/000493196 | NO | NO | PHARMACOLOGICAL STUDY |
| 2020 | R. Recio, M. Mancheño, E. Viedma, J. Villa, M. A. Orellana, J. Lora-Tamayo and F. Chaves | Predictors of mortality in bloodstream infections caused by pseudomonas aeruginosa and impact of antimicrobial resistance and bacterial virulence | 10.1128/AAC.01759-19 | YES | NO | OUT OF TOPIC |
| 2018 | R. Recio, J. Villa, E. Viedma, M. Orellana, J. Lora-Tamayo and F. Chaves | Bacteraemia due to extensively drug-resistant Pseudomonas aeruginosa sequence type 235 high-risk clone: Facing the perfect storm | 10.1016/j.ijantimicag.2018.03.018 | YES | NO | NOT COMPARABLE DATA |
| 2017 | P. Reich, M. Boyle, M. Sullivan, P. G. Hogan, R. Thompson, C. Muenks, C. Kennedy, M. Wallace, C. A. D. Burnham and S. Fritz | Familial and environmental impact on colonization with antibiotic-resistant organisms in the neonatal intensive care unit | 10.1093/ofid/ofx163.1832 | NO | NO | ABSTRACT |
| 2019 | J. Rello, E. Alp and K. E. Vandana | Infectious diseases: the 10 common truths I never believed | 10.1007/s00134-018-5272-z | NO | NO | REVIEW |
| 2020 | J. Rello, C. Sarda, D. Mokart, K. Arvaniti, M. Akova, A. Tabah and E. Azoulay | Antimicrobial Stewardship in Hematological Patients at the intensive care unit: a global cross-sectional survey from the Nine-i Investigators Network | 10.1007/s10096-019-03736-3 | NO | NO | OUT OF TOPIC |
| 2018 | S. Ren, P. S. Fier, H. Ren, A. J. Hoover, D. Hesk, R. Marques and I. Mergelsberg | 34 S: A New Opportunity for the Efficient Synthesis of Stable Isotope Labeled Compounds | 10.1002/chem.201801494 | NO | NO | REVIEW |
| 2018 | N. J. Rhodes, C. E. Cruce, J. N. O’Donnell, R. G. Wunderink and A. R. Hauser | Resistance Trends and Treatment Options in Gram-Negative Ventilator-Associated Pneumonia | 10.1007/s11908-018-0609-x | NO | NO | REVIEW |
| 2010 | E. Riera, M. D. Macia, A. Mena, X. Mulet, J. L. Perez, Y. Ge and A. Oliver | Anti-biofilm and resistance suppression activities of CXA-101 against chronic respiratory infection phenotypes of Pseudomonas aeruginosa strain PAO1 | 10.1093/jac/dkq143 | NO | NO | IN VITRO STUDY |
| 2019 | R. Rigo-Bonnin, J. Gomez-Junyent, L. Garcia-Tejada, E. Benavent, L. Soldevila, F. Tubau and O. Murillo | Measurement of ceftolozane and tazobactam concentrations in plasma by UHPLC-MS/MS. Clinical application in the management of difficult-to-treat osteoarticular infections | 10.1016/j.cca.2018.10.034 | NO | NO | PHARMACOLOGICAL STUDY |
| 2017 | K. R. Rivard, V. Athans, S. W. Lam, S. M. Gordon, G. W. Procop, S. S. Richter and E. Neuner | Impact of antimicrobial stewardship and rapid microarray testing on patients with Gram-negative bacteremia | 10.1007/s10096-017-3008-6 | NO | NO | OUT OF TOPIC |
| 2019 | L. M. Rivas, J. R. W. Martínez, M. Spencer, L. Porte, F. Silva, M. Cifuentes, B. Barraza, P. Rojas, R. A. Bralic, T. T. Tran, W. R. Miller, C. A. Arias, P. García and J. Munita | In vitro activity of ceftolozane/tazobactam (C/T) against enterobacteriaceae and pseudomonas aeruginosa circulating in chile | 10.1093/ofid/ofz360.1456 | NO | NO | IN VITRO STUDY |
| 2018 | F. Robin, M. Auzou, R. Bonnet, R. Lebreuilly, C. Isnard, V. Cattoir and F. Guerin | In Vitro Activity of Ceftolozane-Tazobactam against Enterobacter cloacae Complex Clinical Isolates with Different beta-Lactam Resistance Phenotypes | 10.1128/aac.00675-18 | NO | NO | IN VITRO STUDY |
| 2018 | B. A. Rodriguez, J. E. Girotto and D. P. Nicolau | Ceftazidime/avibactam and ceftolozane/tazobactam: Novel therapy for multidrug resistant gram negative infections in children | 10.2174/1573396314666180308150908 | NO | NO | REVIEW |
| 2017 | C. A. Rodriguez, M. Agudelo, A. F. Zuluaga and O. Vesga | In vivo pharmacodynamics of piperacillin/tazobactam: implications for antimicrobial efficacy and resistance suppression with innovator and generic products | 10.1016/j.ijantimicag.2016.10.011 | NO | NO | PHARMACOLOGICAL STUDY |
| 2018 | O. Rodriguez-Nunez, M. Ripa, L. Morata, C. de la Calle, C. Cardozo, C. Feher, M. Pellice, A. Valcarcel, P. Puerta-Alcalde, F. Marco, C. Garcia-Vidal, A. Del Rio, A. Soriano and J. A. Martinez-Martinez | Evaluation of ceftazidime/avibactam for serious infections due to multidrug-resistant and extensively drug-resistant Pseudomonas aeruginosa | 10.1016/j.jgar.2018.07.010 | NO | NO | CASE REPORT |
| 2019 | M. Rodriguez-Reyes, E. López-Suñé, E. Calvo-Cidoncha, M. Tuset-Creus, C. Codina-Jané and A. Soriano-Viladomiu | Follow-up of recommendations on dose adjustment of ceftolozane/tazobactam in renal failure | 10.1136/ejhpharm-2019-eahpconf.222 | YES | NO | ABSTRACT |
| 2017 | K. A. Rodvold, W. W. Hope and S. E. Boyd | Considerations for effect site pharmacokinetics to estimate drug exposure: concentrations of antibiotics in the lung | 10.1016/j.coph.2017.09.019 | NO | NO | PHARMACOLOGICAL STUDY |
| 2019 | M. M. R. Rojas, C. Lopez, J. Ruiz, J. Pavia, J. Onate and C. Hernandez-Gomez | Antimicrobial Resistance patterns of Enterobacteriaceae and Pseudomonas aeruginosa from Colombian clinical isolates. 2017-2018 | 10.1093/ofid/ofz360.2155 | NO | NO | IN VITRO STUDY |
| 2019 | E. Rojo-Molinero, M. D. MacIà and A. Oliver | Social behavior of antibiotic resistant mutants within pseudomonas aeruginosa biofilm communities | 10.3389/fmicb.2019.00570 | NO | NO | IN VITRO STUDY |
| 2018 | K. V. I. Rolston, B. Gerges, I. Raad, S. L. Aitken, R. Reitzel and R. Prince | In vitro activity of cefiderocol and comparator agents against gram-negative isolates from cancer patients | 10.1093/ofid/ofy210.1206 | NO | NO | IN VITRO STUDY |
| 2019 | A. Romanowski, B. Hamandi, J. Murdoch and G. Wong | Drug utilization evaluation of ceftolozane/ tazobactam in a canadian academic teaching hospital system |  | NO | NO | OUT OF TOPIC |
| 2019 | J. D. Rosen, O. Stojadinovic, J. D. McBride, R. Rico, J. H. Cho-Vega and A. J. Nichols | Bowel-associated dermatosis-arthritis syndrome (BADAS) in a patient with cystic fibrosis | 10.1016/j.jdcr.2018.08.029 | NO | NO | CASE REPORT |
| 2020 | G. Royer, F. Fourreau, B. Boulanger, M. Mercier-Darty, D. Ducellier, F. Cizeau, A. Potron, I. Podglajen, N. Mongardon and J. W. Decousser | Local outbreak of extended-spectrum β-lactamase SHV2a-producing Pseudomonas aeruginosa reveals the emergence of a new specific sub-lineage of the international ST235 high-risk clone | 10.1016/j.jhin.2019.07.014 | NO | NO | CASE REPORT |
| 2020 | J. Ruiz, A. Ferrada, M. Salavert, M. Gordon, E. Villarreal, A. Castellanos-Ortega and P. Ramirez | Ceftolozane/Tazobactam Dosing Requirements Against Pseudomonas aeruginosa Bacteremia | 10.1177/1559325819885790 | NO | NO | PHARMACOLOGICAL STUDY |
| 2017 | P. Ruiz-Garbajosa and R. Cantón | Epidemiology of antibiotic resistance in pseudomonas aeruginosa. Implications for empiric and definitive therapy |  | NO | NO | REVIEW |
| 2016 | G. Sacha, E. Neuner, V. Athans, A. Pallotta, S. Bass, S. Bauer and K. Brizendine | Clinical experience with ceftolozane/tazobactam at a large academic medical center | 10.1093/ofid/ofw172.1599 | YES | NO | ABSTRACT |
| 2017 | G. L. Sacha, E. A. Neuner, V. Athans, S. N. Bass, A. Pallotta, K. R. Rivard, S. R. Bauer and K. D. Brizendine | Retrospective Evaluation of the Use of Ceftolozane/Tazobactam at a Large Academic Medical Center | 10.1097/IPC.0000000000000553 | NO | NO | CASE SERIES |
| 2018 | H. Sader and M. Castanheira | Ceftazidime-avibactam activity against gram-negative bacteria from intensive care unit patients | 10.1097/01.ccm.0000528707.26692.3d | NO | NO | ABSTRACT |
| 2019 | H. Sader, M. Castanheira, R. E. Mendes, C. G. Carvalhaes and R. K. Flamm | Frequency and antimicrobial susceptibility of bacteria isolated from patients hospitalized with pneumonia in United States medical centers (2017-2018) |  | NO | NO | IN VITRO STUDY |
| 2012 | H. Sader, R. Flamm, D. Farrell and R. Jones | Activity of the novel antimicrobial ceftolozane/tazobactam (CXA-201) tested against contemporary clinical strains from European hospitals | 10.1111/j.1469-0691.2012.03802.x | NO | NO | IN VITRO STUDY |
| 2020 | H. S. Sader, C. G. Carvalhaes, L. R. Duncan and D. Shortridge | Antimicrobial Activity of Ceftolozane-Tazobactam and Comparators against Clinical Isolates of Haemophilus influenzae from the United States and Europe | 10.1128/aac.00211-20 | NO | NO | IN VITRO STUDY |
| 2019 | H. S. Sader, C. G. Carvalhaes, R. E. Mendes, M. Castanheira and R. K. Flamm | Comparison of cefepime-zidebactam (WCK 5222), ceftazidime-avibactam, and ceftolozane-tazobactam tested against gram-negative organisms causing pneumonia in United States hospitals in 2018 | 10.1093/ofid/ofz360.1892 | NO | NO | IN VITRO STUDY |
| 2019 | H. S. Sader, C. G. Carvalhaes, D. Shortridge and M. D. Huband | Antimicrobial activity of novel β-lactamase inhibitor combinations tested against organisms causing complicated urinary tract infections in United States medical centers | 10.1093/ofid/ofz360.1309 | NO | NO | IN VITRO STUDY |
| 2018 | H. S. Sader, M. Castanheira, R. E. Mendes and R. K. Flamm | Frequency and antimicrobial susceptibility of Gram-negative bacteria isolated from patients with pneumonia hospitalized in ICUs of US medical centres (2015–17) | 10.1093/jac/dky279 | NO | NO | IN VITRO STUDY |
| 2019 | H. S. Sader, M. Castanheira, J. M. Streit and R. K. Flamm | Frequency of occurrence and antimicrobial susceptibility of bacteria isolated from patients hospitalized with bloodstream infections in United States medical centers (2015–2017) | 10.1016/j.diagmicrobio.2019.06.002 | NO | NO | IN VITRO STUDY |
| 2014 | H. S. Sader, D. J. Farrell, M. Castanheira, R. K. Flamm and R. N. Jones | Antimicrobial activity of ceftolozane/tazobactam tested against Pseudomonas aeruginosa and Enterobacteriaceae with various resistance patterns isolated in European hospitals (2011-12) | 10.1093/jac/dku184 | NO | NO | IN VITRO STUDY |
| 2014 | H. S. Sader, D. J. Farrell, R. K. Flamm and R. N. Jones | Ceftolozane/tazobactam activity tested against aerobic Gram-negative organisms isolated from intra-abdominal and urinary tract infections in European and United States hospitals (2012) | 10.1016/j.jinf.2014.04.004 | NO | NO | IN VITRO STUDY |
| 2018 | H. S. Sader, R. K. Flamm, C. G. Carvalhaes and M. Castanheira | Antimicrobial susceptibility of pseudomonas aeruginosa to ceftazidime-avibactam, ceftolozane-tazobactam, piperacillin-tazobactam, and meropenem stratified by U.S. census divisions: Results from the 2017 INFORM program | 10.1128/AAC.01587-18 | NO | NO | IN VITRO STUDY |
| 2020 | H. S. Sader, R. K. Flamm, C. G. Carvalhaes and M. Castanheira | Comparison of ceftazidime-avibactam and ceftolozane-tazobactam in vitro activities when tested against gram-negative bacteria isolated from patients hospitalized with pneumonia in United States medical centers (2017–2018) | 10.1016/j.diagmicrobio.2019.05.005 | NO | NO | IN VITRO STUDY |
| 2018 | H. S. Sader, R. K. Flamm and M. Castanheira | Comparison of ceftazidime-avibactam and ceftolozane-tazobactam in vitro activities when tested against gram-negative bacteria isolated from patients hospitalized with pneumonia in us medical centers (2017) | 10.1093/ofid/ofy210.2080 | NO | NO | IN VITRO STUDY |
| 2019 | H. S. Sader, R. K. Flamm, M. Castanheira and R. E. Mendes | Comparison of ceftazidime-avibactam, ceftolozane-tazobactam, piperacillin-tazobactam, and meropenem activities when tested against gram-negative organisms isolated from complicated urinary tract infections | 10.1093/ofid/ofz360.1300 | NO | NO | IN VITRO STUDY |
| 2018 | H. S. Sader, R. K. Flamm, G. E. Dale, P. R. Rhomberg and M. Castanheira | Murepavadin activity tested against contemporary (2016-17) clinical isolates of XDR Pseudomonas aeruginosa | 10.1093/jac/dky227 | NO | NO | IN VITRO STUDY |
| 2019 | H. S. Sader, M. D. Huband, C. G. Carvalhaes and M. Castanheira | Frequency and antimicrobial susceptibility of bacteria isolated from patients hospitalized with pneumonia in US medical centers during 2018 | 10.1093/ofid/ofz360.1895 | NO | NO | IN VITRO STUDY |
| 2011 | H. S. Sader, P. R. Rhomberg, D. J. Farrell and R. N. Jones | Antimicrobial activity of CXA-101, a novel cephalosporin tested in combination with tazobactam against Enterobacteriaceae, Pseudomonas aeruginosa, and Bacteroides fragilis strains having various resistance phenotypes | 10.1128/AAC.01737-10 | NO | NO | IN VITRO STUDY |
| 2014 | H. S. Sader, P. R. Rhomberg and R. N. Jones | Post-β-lactamase-inhibitor effect of tazobactam in combination with ceftolozane on extended-spectrum-β-lactamase-producing strains | 10.1128/AAC.02398-13 | NO | NO | IN VITRO STUDY |
| 2019 | A. Sakurai, A. Q. Dinh, B. Hanson, W. C. Shropshire, T. T. Tran, A. Wanger, C. A. Arias and W. R. Miller | Evolution of carbapenem-resistant pseudomonas aeruginosa (CR-PA) genetic lineages and acquisition of β-lactamase enzymes | 10.1093/ofid/ofz360.554 | NO | NO | IN VITRO STUDY |
| 2016 | P. Salgado, F. Gilsanz and E. Maseda | [Resistant gram-negative bacteria. Therapeutic approach and risk factors] |  | NO | NO | REVIEW |
| 2017 | I. Sánchez-Diener, L. Zamorano, C. López-Causapé, G. Cabot, X. Mulet, C. Peña, R. Del Campo, R. Cantón, A. Doménech-Sánchez, L. Martínez-Martínez, S. C. Arcos, A. Navas and A. Oliver | Interplay among resistance profiles, high-risk clones, and virulence in the Caenorhabditis elegans Pseudomonas aeruginosa infection model | 10.1128/AAC.01586-17 | NO | NO | IN VITRO STUDY |
| 2018 | B. Sanchez-Jimenez, N. C. Chavez-Tapia, J. C. Jakobsen, D. Nikolova and C. Gluud | Antibiotic prophylaxis versus placebo or no intervention for people with cirrhosis and variceal bleeding | 10.1002/14651858.CD013175 | NO | NO | PROTOCOL |
| 2018 | B. Sanchez‐Jimenez, N. C. Chavez‐Tapia, J. C. Jakobsen, D. Nikolova and C. Gluud | Antibiotic prophylaxis for people with cirrhosis and variceal bleeding | 10.1002/14651858.CD013214 | NO | NO | PROTOCOL |
| 2019 | L. M. Saraca, C. Di Giuli, F. Sicari, G. Priante, F. Lavagna and D. Francisci | Use of Ceftolozane-Tazobactam in Patient with Severe Medium Chronic Purulent Otitis by XDR Pseudomonas aeruginosa | 10.1155/2019/2683701 | NO | NO | CASE REPORT |
| 2019 | O. Saran, B. Sulik-Tyszka, G. W. Basak and M. M. Wroblewska | Activity of Ceftolozane/Tazobactam Against Gram-Negative Rods of the Family Enterobacteriaceae and Pseudomonas Spp. Isolated from Onco-Hematological Patients Hospitalized in a Clinical Hospital in Poland | 10.12659/msm.913352 | NO | NO | IN VITRO STUDY |
| 2017 | P. Sarkar, V. Yarlagadda, C. Ghosh and J. Haldar | A review on cell wall synthesis inhibitors with an emphasis on glycopeptide antibiotics | 10.1039/c6md00585c | NO | NO | REVIEW |
| 2017 | M. J. Satlin and T. J. Walsh | Multidrug-resistant Enterobacteriaceae, Pseudomonas aeruginosa, and vancomycin-resistant Enterococcus: Three major threats to hematopoietic stem cell transplant recipients | 10.1111/tid.12762 | NO | NO | REVIEW |
| 2018 | Y. Sato, K. Hiramatsu, Y. Suzuki, R. Tanaka, T. Kaneko, K. Nonoshita, M. Ogata, J. I. Kadota and H. Itoh | Optimal trough concentration of teicoplanin in febrile neutropenic patients with hematological malignancy | 10.1159/000481725 | NO | NO | PHARMACOLOGICAL STUDY |
| 2016 | E. Sauvage and M. Terrak | Glycosyltransferases and transpeptidases/penicillin-binding proteins: Valuable targets for new antibacterials | 10.3390/antibiotics5010012 | NO | NO | REVIEW |
| 2019 | A. M. L. Scavuzzi, E. M. B. Beltrão, E. F. Firmo, M. de Oliveira É, F. G. Beserra and A. C. D. S. Lopes | Emergence of blaVIM-2, blaNDM-1, blaIMP-7 and blaGES-1 in blaKPC-2-harbouring Pseudomonas aeruginosa isolates in Brazil | 10.1016/j.jgar.2019.09.009 | NO | NO | IN VITRO STUDY |
| 2016 | A. J. Schaeffer and L. E. Nicolle | Urinary tract infections in older men | 10.1056/NEJMcp1503950 | YES | NO | LETTER |
| 2017 | F. Schaumburg, S. Bletz, A. Mellmann, K. Becker and E. A. Idelevich | Susceptibility of MDR Pseudomonas aeruginosa to ceftolozane/tazobactam and comparison of different susceptibility testing methods | 10.1093/jac/dkx253 | NO | NO | IN VITRO STUDY |
| 2018 | S. M. Schmidt-Malan, A. J. Mishra, A. Mushtaq, C. L. Brinkman and R. Patel | In Vitro Activity of Imipenem-Relebactam and Ceftolozane-Tazobactam against Resistant Gram-Negative Bacilli | 10.1128/aac.00533-18 | NO | NO | IN VITRO STUDY |
| 2019 | S. M. Schmidt-Malan, A. J. Mishra, A. Mushtaq, C. L. Brinkman and R. Patel | Correction: In vitro activity of imipenem-relebactam and ceftolozane-tazobactam against resistant gram-negative bacilli (Antimicrobial Agents and Chemotherapy (2018) 62:8 (e00533-18) DOI: 10.1128/AAC.02576-17) | 10.1128/AAC.02563-18 | NO | NO | IN VITRO STUDY |
| 2016 | C. Schneeberger, F. Holleman and S. E. Geerlings | Febrile urinary tract infections: Pyelonephritis and urosepsis | 10.1097/QCO.0000000000000227 | NO | NO | REVIEW |
| 2017 | E. R. Schwarz, K. G. Oikonomou, M. Reynolds, J. Kim, R. L. Balmiki and S. A. Sterling | Extranodal NK/T-cell lymphoma, nasal type, presenting as refractory pseudomonas aeruginosa facial cellulitis | 10.1177/2324709617716471 | NO | NO | CASE REPORT |
| 2019 | A. Scobie, E. L. Budd, R. J. Harris, S. Hopkins and N. Shetty | Antimicrobial stewardship: An evaluation of structure and process and their association with antimicrobial prescribing in NHS hospitals in England | 10.1093/jac/dky538 | NO | NO | OUT OF TOPIC |
| 2016 | L. J. Scott | Ceftolozane/Tazobactam: A Review in Complicated Intra-Abdominal and Urinary Tract Infections | 10.1007/s40265-015-0524-5 | NO | NO | REVIEW |
| 2018 | J. Sebaaly, J. A. Woods and K. A. Wargo | A Review of Ceftolozane/Tazobactam for the Treatment of Infections Caused by Multidrug-Resistant Pathogens | 10.1097/IPC.0000000000000638 | NO | NO | REVIEW |
| 2018 | H. Seifert, B. Korber-Irrgang and M. Kresken | In-vitro activity of ceftolozane/tazobactam against Pseudomonas aeruginosa and Enterobacteriaceae isolates recovered from hospitalized patients in Germany | 10.1016/j.ijantimicag.2017.06.024 | NO | NO | IN VITRO STUDY |
| 2019 | F. Senchyna, R. L. Gaur, J. Sandlund, C. Truong, G. Tremintin, D. Kultz, C. A. Gomez, F. B. Tamburini, T. Andermann, A. Bhatt, I. Tickler, N. Watz, I. Budvytiene, G. Shi, F. C. Tenover and N. Banaei | Diversity of resistance mechanisms in carbapenem-resistant Enterobacteriaceae at a health care system in Northern California, from 2013 to 2016 | 10.1016/j.diagmicrobio.2018.10.004 | NO | NO | IN VITRO STUDY |
| 2017 | E. Senneville | Antibacterial Treatment in Diabetic Foot Infections | 10.1159/000480099 | NO | NO | REVIEW |
| 2017 | E. J. Septimus, C. M. Coopersmith, J. Whittle, C. P. Hale, N. O. Fishman and T. J. Kim | Sepsis National Hospital Inpatient Quality Measure (SEP-1): Multistakeholder Work Group Recommendations for Appropriate Antibiotics for the Treatment of Sepsis | 10.1093/cid/cix603 | NO | NO | OUT OF TOPIC |
| 2018 | M. M. Sfeir | The GAIN Act legislation to combat antimicrobial resistance: Where do we stand? | 10.1017/ice.2018.252 | NO | NO | LETTER |
| 2019 | M. Shafiekhani, M. Mirjalili and A. Vazin | Prevalence, risk factors and treatment of the most common gram-negative bacterial infections in liver transplant recipients: A review | 10.2147/IDR.S226217 | NO | NO | REVIEW |
| 2015 | P. Shenoy | What is new in the areas of pharmaceuticals? |  | NO | NO | REVIEW |
| 2018 | C. C. Sheu, S. Y. Lin, Y. T. Chang, C. Y. Lee, Y. H. Chen and P. R. Hsueh | Management of infections caused by extended-spectrum β–lactamase-producing Enterobacteriaceae: current evidence and future prospects | 10.1080/14787210.2018.1436966 | NO | NO | REVIEW |
| 2020 | R. K. Shields | Case commentary: The need for cefiderocol is clear, but are the supporting clinical data? | 10.1128/AAC.00059-20 | NO | NO | COMMENT |
| 2018 | R. K. Shields, C. J. Clancy, A. W. Pasculle, E. G. Press, G. Haidar, B. Hao, L. Chen, B. N. Kreiswirth and M. H. Nguyen | Verification of Ceftazidime-Avibactam and Ceftolozane-Tazobactam Susceptibility Testing Methods against Carbapenem-Resistant Enterobacteriaceae and Pseudomonas aeruginosa | 10.1128/JCM.01093-17 | YES | NO | ABSTRACT |
| 2018 | R. K. Shields, G. Haidar, B. A. Potoski, Y. Doi, R. V. Marini, M. H. Nguyen and C. J. Clancy | Ceftolozane/tazobactam (C/T) against multidrug-resistant pseudomonas aeruginosa (MDR-PA) infections: Clinical efficacy, and baseline and emergent resistance | 10.1093/ofid/ofy210.2091 | NO | NO | ABSTRACT |
| 2013 | D. M. Shlaes | New β-lactam-β-lactamase inhibitor combinations in clinical development | 10.1111/nyas.12010 | NO | NO | REVIEW |
| 2020 | D. M. Shlaes | The economic conundrum for antibacterial drugs | 10.1128/AAC.02057-19 | NO | NO | COMMENT |
| 2019 | D. Shortridge, S. J. R. Arends, L. R. Duncan, J. M. Streit and R. K. Flamm | Ceftolozane-tazobactam activity against difficult-to-treat resistance in pseudomonas aeruginosa from bloodstream infections in us hospitals | 10.1093/ofid/ofz360.1453 | NO | NO | ABSTRACT |
| 2017 | D. Shortridge, M. Castanheira, M. A. Pfaller and R. K. Flamm | Ceftolozane-tazobactam activity against Pseudomonas aeruginosa clinical isolates from U.S. hospitals: Report from the PACTS antimicrobial surveillance program, 2012 to 2015 | 10.1128/AAC.00465-17 | NO | NO | IN VITRO STUDY |
| 2017 | D. Shortridge, M. Castanheira, H. S. Sader, J. M. Streit and R. K. Flamm | Susceptibility of ceftolozane/tazobactam against isolates collected from intensive care unit patients in european hospitals (2014-2016) | 10.1186/s13054-017-1629-x | NO | NO | IN VITRO STUDY |
| 2017 | D. Shortridge, L. R. Duncan, M. A. Pfaller and R. K. Flamm | Antimicrobial activity of ceftolozane-tazobactam tested against contemporary (2012-2016) enterobacteriaceae and pseudomonas aeruginosa from ICU vs. non-ICU isolates collected in US medical centers | 10.1093/ofid/ofx163.894 | NO | NO | ABSTRACT |
| 2017 | D. Shortridge, L. R. Duncan, M. A. Pfaller and R. K. Flamm | Activity of ceftolozane-tazobactam and comparators when tested against bacterial surveillance isolates collected from pediatric patients in the us during 2012-2016 as part of a global surveillance program | 10.1093/ofid/ofxl63.898 | NO | NO | IN VITRO STUDY |
| 2017 | D. Shortridge, L. R. Duncan, M. A. Pfaller and R. K. Flamm | Antimicrobial activity of ceftolozane-tazobactam tested against contemporary (2012-2016) enterobacteriaceae and pseudomonas aeruginosa isolates by us census division | 10.1093/ofid/ofx163.912 | NO | NO | ABSTRACT |
| 2019 | D. Shortridge, L. R. Duncan, M. A. Pfaller and R. K. Flamm | Activity of ceftolozane-tazobactam and comparators when tested against Gram-negative isolates collected from paediatric patients in the USA and Europe between 2012 and 2016 as part of a global surveillance programme | 10.1016/j.ijantimicag.2019.01.015 | NO | NO | IN VITRO STUDY |
| 2019 | D. Shortridge, M. A. Pfaller, S. J. R. Arends, J. Raddatz, D. D. DePestel and R. K. Flamm | Comparison of the In Vitro Susceptibility of Ceftolozane-Tazobactam With the Cumulative Susceptibility Rates of Standard Antibiotic Combinations When Tested Against Pseudomonas aeruginosa From ICU Patients With Bloodstream Infections or Pneumonia | 10.1093/ofid/ofz240 | NO | NO | IN VITRO STUDY |
| 2018 | D. Shortridge, M. A. Pfaller, M. Castanheira and R. K. Flamm | Antimicrobial activity of ceftolozane-tazobactam tested against Enterobacteriaceae and Pseudomonas aeruginosa collected from patients with bloodstream infections isolated in United States hospitals (2013–2015) as part of the Program to Assess Ceftolozane-Tazobactam Susceptibility (PACTS) surveillance program | 10.1016/j.diagmicrobio.2018.05.011 | NO | NO | IN VITRO STUDY |
| 2018 | D. Shortridge, M. A. Pfaller, M. Castanheira and R. K. Flamm | Antimicrobial Activity of Ceftolozane-Tazobactam Tested Against Enterobacteriaceae and Pseudomonas aeruginosa with Various Resistance Patterns Isolated in U.S. Hospitals (2013-2016) as Part of the Surveillance Program: Program to Assess Ceftolozane-Tazobactam Susceptibility | 10.1089/mdr.2017.0266 | NO | NO | IN VITRO STUDY |
| 2019 | D. Shortridge, M. A. Pfaller, J. M. Streit and R. K. Flamm | Antimicrobial activity of ceftolozane/tazobactam tested against contemporary (2015-2017) Pseudomonas aeruginosa isolates from a global surveillance programme | 10.1016/j.jgar.2019.10.009 | NO | NO | IN VITRO STUDY |
| 2019 | M. A. Sid Ahmed, H. Abdel Hadi, A. A. I. Hassan, S. Abu Jarir, M. A. Al-Maslamani, N. O. Eltai, K. M. Dousa, A. M. Hujer, A. A. Sultan, B. Soderquist, R. A. Bonomo, E. B. Ibrahim, J. Jass and A. S. Omrani | Evaluation of in vitro activity of ceftazidime/avibactam and ceftolozane/tazobactam against MDR Pseudomonas aeruginosa isolates from Qatar | 10.1093/jac/dkz379 | NO | NO | IN VITRO STUDY |
| 2020 | E. Sieswerda, M. Van Den Brand, R. B. Van Den Berg, J. Sträter, L. Schouls, K. Van Dijk and A. E. Budding | Successful rescue treatment of sepsis due to a pandrug-resistant, NDM-producing Klebsiella pneumoniae using aztreonam powder for nebulizer solution as intravenous therapy in combination with ceftazidime/avibactam | 10.1093/jac/dkz495 | YES | NO | LETTER |
| 2018 | L. L. Silver | The antibiotic future | 10.1007/7355_2017_24 | NO | NO | REVIEW |
| 2016 | S. Simar, D. Sibley, D. Ashcraft, J. Li and G. Pankey | In vitro resistance to two new cephalosporin/β-lactamase inhibitor combination antibiotics by pseudomonas aeruginosa isolates from ochsner medical center patients |  | NO | NO | IN VITRO STUDY |
| 2019 | F. B. Sime, M. Lassig-Smith, T. Starr, J. Stuart, S. Pandey, S. L. Parker, S. C. Wallis, J. Lipman and J. A. Roberts | Population pharmacokinetics of unbound ceftolozane and tazobactam in critically ill patients without renal dysfunction | 10.1128/AAC.01265-19 | NO | NO | PHARMACOLOGICAL STUDY |
| 2019 | F. B. Sime, M. Lassig-Smith, T. Starr, J. Stuart, S. Pandey, S. L. Parker, S. C. Wallis, J. Lipman and J. A. Roberts | A Population Pharmacokinetic Model-Guided Evaluation of Ceftolozane-Tazobactam Dosing in Critically Ill Patients Undergoing Continuous Venovenous Hemodiafiltration | 10.1128/aac.01655-19 | NO | NO | PHARMACOLOGICAL STUDY |
| 2017 | J. Sinko | [Clinical aspects of severe infections caused by antibiotic-resistant Gram-negative bacteria. The Empire strikes back?] | 10.1556/650.2017.30858 | NO | NO | REVIEW |
| 2015 | M. J. Skalweit | Profile of ceftolozane/tazobactam and its potential in the treatment of complicated intra-abdominal infections | 10.2147/DDDT.S61436 | NO | NO | REVIEW |
| 2017 | E. Skoglund, H. Abodakpi, L. Diaz, R. Rios, T. T. Tran, C. A. Arias and V. H. Tam | Ceftolozane/tazobactam resistance in Pseudomonas aeruginosa |  | NO | NO | IN VITRO STUDY |
| 2018 | E. Skoglund, H. Abodakpi, R. Rios, L. Diaz, E. De La Cadena, A. Q. Dinh, J. Ardila, W. R. Miller, J. M. Munita, C. A. Arias, V. H. Tam and T. T. Tran | In Vivo Resistance to Ceftolozane/Tazobactam in Pseudomonas aeruginosa Arising by AmpC- and Non-AmpC-Mediated Pathways | 10.1155/2018/9095203 | NO | NO | CASE REPORT |
| 2019 | E. Skoglund, H. Abodakpi, R. Rios, L. Diaz, E. De La Cadena, A. Q. Dinh, J. Ardila, W. R. Miller, J. M. Munita, C. A. Arias, V. H. Tam and T. T. Tran | Corrigendum to "In Vivo Resistance to Ceftolozane/Tazobactam in Pseudomonas aeruginosa Arising by AmpC- and Non-AmpC-Mediated Pathways" | 10.1155/2019/8314349 | NO | NO | CASE REPORT |
| 2017 | E. Skoglund, K. R. Ledesma, T. M. Lasco and V. H. Tam | Ceftolozane/tazobactam activity against meropenem-non-susceptible Pseudomonas aeruginosa bloodstream infection isolates | 10.1016/j.jgar.2017.10.016 | NO | NO | IN VITRO STUDY |
| 2018 | A. Slack, F. Parsonson, K. Cronin, C. Engler and R. Norton | Activity of ceftolozane-tazobactam against burkholderia pseudomallei | 10.4269/ajtmh.17-0049 | NO | NO | IN VITRO STUDY |
| 2016 | J. R. Smith, A. Arya, J. Yim, K. E. Barber, J. Hallesy, N. B. Singh and M. J. Rybak | Daptomycin in combination with ceftolozane-tazobactam or cefazolin against daptomycin-susceptible and -nonsusceptible Staphylococcus aureus in an in vitro, hollow-fiber model | 10.1128/AAC.01666-15 | NO | NO | IN VITRO STUDY |
| 2010 | A. R. Smyth and M. N. Hurley | Targeting the Pseudomonas aeruginosa biofilm to combat infections in patients with cystic fibrosis | 10.1358/dof.2010.035.012.1537937 | NO | NO | REVIEW |
| 2012 | D. R. Snydman, N. V. Jacobus and L. A. Mc Dermott | Activity of ceftolozane/tazobactam (CXA-201) against 270 recent isolates from the Bacteroides group | 10.1111/j.1469-0691.2012.03802.x | NO | NO | IN VITRO STUDY |
| 2014 | D. R. Snydman, L. A. McDermott and N. V. Jacobus | Activity of ceftolozane-tazobactam against a broad spectrum of recent clinical anaerobic isolates | 10.1128/AAC.02253-13 | NO | NO | IN VITRO STUDY |
| 2019 | W. So, J. Shurko, R. Galega, R. Quilitz, J. N. Greene and G. C. Lee | Mechanisms of high-level ceftolozane/tazobactam resistance in Pseudomonas aeruginosa from a severely neutropenic patient and treatment success from synergy with tobramycin | 10.1093/jac/dky393 | NO | NO | CASE REPORT |
| 2016 | J. Solomkin, M. Popejoy, B. Miller, J. Long, E. Hershberger and J. Mazuski | Clinical outcomes of short-course versus long-course treatment with ceftolozane/tazobactam and meropenem for complicated intraabdominal infections | 10.1089/sur.2016.29005.abstracts | NO | NO | ABSTRACT |
| 2015 | J. S. Solomkin, E. Hershberger and C. Eckmann | Response to Spellberg and Brass | 10.1093/cid/civ896 | NO | NO | LETTER |
| 2016 | R. L. Soon, J. R. Lenhard, Z. P. Bulman, P. N. Holden, P. Kelchlin, J. N. Steenbergen, L. V. Friedrich, A. Forrest and B. T. Tsuji | Combinatorial pharmacodynamics of ceftolozane-tazobactam against genotypically defined β-lactamase-producing Escherichia coli: Insights into the pharmacokinetics/pharmacodynamics of β-lactam-P-lactamase inhibitor combinations | 10.1128/AAC.02635-15 | NO | NO | PHARMACOLOGICAL STUDY |
| 2017 | R. L. Soon, J. R. Lenhard, Z. P. Bulman, P. N. Holden, P. Kelchlin, J. N. Steenbergen, L. V. Friedrich, A. Forrest and B. T. Tsuji | In vitro pharmacodynamic evaluation of ceftolozane/tazobactam against β-lactamase-producing Escherichia coli in a hollow-fibre infection model | 10.1016/j.ijantimicag.2016.07.015 | NO | NO | IN VITRO STUDY |
| 2014 | M. Sorbera, E. Chung, C. W. Ho and N. Marzella | Ceftolozane/tazobactam: A new option in the treatment of complicated gram-negative infections |  | NO | NO | REVIEW |
| 2019 | A. Soriano, L. Puzniak, D. Paterson, F. Thalhalmmer, S. Kluge, P. Viale and S. Kaul | Study of prescribing patterns and effectiveness of ceftolozane-tazobactam (C/T): Real-world analysis (Spectra): A multi-national, multicenter observational study | 10.1093/ofid/ofz360.1957 | YES | NO | ABSTRACT |
| 2019 | P. Soukup, A. C. Faust, V. Edpuganti, W. C. Putnam and J. A. McKinnell | Steady-State Ceftazidime-Avibactam Serum Concentrations and Dosing Recommendations in a Critically Ill Patient Being Treated for Pseudomonas aeruginosa Pneumonia and Undergoing Continuous Venovenous Hemodiafiltration | 10.1002/phar.2338 | NO | NO | CASE REPORT |
| 2017 | A. Sousa Dominguez, M. T. Perez-Rodriguez, A. Nodar, L. Martinez-Lamas, A. Perez-Landeiro and M. Crespo Casal | Successful treatment of MDR Pseudomonas aeruginosa skin and soft-tissue infection with ceftolozane/tazobactam | 10.1093/jac/dkw526 | NO | NO | CASE REPORT |
| 2015 | B. Spellberg and E. P. Brass | Noninferiority Doesn't Mean Not Inferior | 10.1093/cid/civ895 | NO | NO | LETTER |
| 2019 | A. M. Spera, S. Esposito and P. Pagliano | Emerging antibiotic resistance: carbapenemase-producing enterobacteria. New bad bugs, still no new drugs |  | NO | NO | REVIEW |
| 2017 | P. Srinivas and K. Rivard | Polymyxin Resistance in Gram-negative Pathogens | 10.1007/s11908-017-0596-3 | NO | NO | REVIEW |
| 2019 | M. Stanzani, M. Cricca, C. Sassi, E. Sutto, G. De Cicco, F. Bonifazi, C. Bertuzzi, F. Bacci, S. Paolini, M. Cavo and R. E. Lewis | Saprochaete clavata infections in patients undergoing treatment for haematological malignancies: A report of a monocentric outbreak and review of the literature | 10.1111/myc.12978 | NO | NO | REVIEW |
| 2017 | S. N. Steiger, R. R. Comito and D. P. Nicolau | Clinical and economic implications of urinary tract infections | 10.1080/14737167.2017.1358618 | NO | NO | REVIEW |
| 2015 | J. Stein | Product updates for pharmacists 1-10-2015 |  | NO | NO | PHARMACOLOGICAL STUDY |
| 2018 | A. Stewart, J. A. Roberts, S. C. Wallis, A. M. Allworth, A. Legg and K. L. McCarthy | Evidence of clinical response and stability of Ceftolozane/Tazobactam used to treat a carbapenem-resistant Pseudomonas Aeruginosa lung abscess on an outpatient antimicrobial program | 10.1016/j.ijantimicag.2018.02.008 | NO | NO | CASE REPORT |
| 2018 | K. Stokem, J. B. Zuckerman, D. P. Nicolau, M. Wungwattana and E. H. Sears | Use of ceftolozane-tazobactam in a cystic fibrosis patient with multidrug-resistant pseudomonas infection and renal insufficiency | 10.1016/j.rmcr.2017.10.012 | NO | NO | CASE REPORT |
| 2017 | K. R. Stover, C. M. Bland and J. C. Gallagher | The point of antimicrobial susceptibility testing is to inform antimicrobial prescribing | 10.1093/cid/ciw686 | NO | NO | LETTER |
| 2011 | M. V. Stundick, M. Metz, A. Sampath and J. C. Larsen | State-of-the-art therapeutic medical countermeasures for bacterial threat agents | 10.1002/ddr.20462 | NO | NO | REVIEW |
| 2017 | P. A. Su, Y. C. Chuang, R. E. Badal and W. L. Yu | In vitro activity of ceftolozane-tazobactam against Pseudomonas aeruginosa and Enterobacteriaceae isolates from medical center in Taiwan, 2012-2015 |  | NO | NO | IN VITRO STUDY |
| 2019 | P. Subbareddy and T. E. Divakar | Analytical method development and validation for the simultaneous estimation of ceftolozane and tazobactam in pure and pharmaceutical dosage form by RP-HPLC | 10.26452/ijrps.v10i1.1908 | NO | NO | PHARMACOLOGICAL STUDY |
| 2015 | A. J. Sucher, E. B. Chahine, P. Cogan and M. Fete | Ceftolozane/Tazobactam: A New Cephalosporin and β-Lactamase Inhibitor Combination | 10.1177/1060028015593293 | NO | NO | REVIEW |
| 2018 | C. Sutherland, S. A. Abuhussain and D. P. Nicolau | Single β-lactams v. combination regimens: Assessing the probability that an active agent would be selected when considering empiric therapy for P. aeruginosa | 10.1093/ofid/ofy210.1832 | NO | NO | ABSTRACT |
| 2015 | C. A. Sutherland, J. L. Crandon and D. P. Nicolau | Defining Extended Spectrum β-Lactamases: Implications of Minimum Inhibitory Concentration-Based Screening Versus Clavulanate Confirmation Testing | 10.1007/s40121-015-0094-6 | NO | NO | IN VITRO STUDY |
| 2015 | C. A. Sutherland and D. P. Nicolau | Susceptibility Profile of Ceftolozane/Tazobactam and Other Parenteral Antimicrobials Against Escherichia coli, Klebsiella pneumoniae, and Pseudomonas aeruginosa from US Hospitals | 10.1016/j.clinthera.2015.05.501 | NO | NO | IN VITRO STUDY |
| 2016 | C. A. Sutherland and D. P. Nicolau | Development of an HPLC Method for the Determination of Ceftolozane/Tazobactam in Biological and Aqueous Matrixes | 10.1093/chromsci/bmw047 | NO | NO | OUT OF TOPIC |
| 2017 | C. A. Sutherland and D. P. Nicolau | Potency of parenteral antimicrobials including ceftolozane/tazobactam against nosocomial respiratory tract pathogens: Considerations for empiric and directed therapy | 10.21037/jtd.2017.01.26 | NO | NO | IN VITRO STUDY |
| 2019 | C. A. Sutherland, C. Ozbal and D. P. Nicolau | Development of an HPLC-MS/MS method for the determination of ceftolozane/tazobactam in bronchoalveolar lavage fluid | 10.4155/fsoa-2018-0079 | NO | NO | OUT OF TOPIC |
| 2016 | C. A. Sutherland, J. E. Verastegui and D. P. Nicolau | In vitro potency of amikacin and comparators against E. coli, K. pneumoniae and P. aeruginosa respiratory and blood isolates | 10.1186/s12941-016-0155-z | NO | NO | IN VITRO STUDY |
| 2016 | L. S. Syue, Y. H. Chen, W. C. Ko and P. R. Hsueh | New drugs for the treatment of complicated intra-abdominal infections in the era of increasing antimicrobial resistance | 10.1016/j.ijantimicag.2015.12.021 | NO | NO | REVIEW |
| 2007 | S. Takeda, Y. Ishii, K. Hatano, K. Tateda and K. Yamaguchi | Stability of FR264205 against AmpC β-lactamase of Pseudomonas aeruginosa | 10.1016/j.ijantimicag.2007.05.019 | NO | NO | OUT OF TOPIC |
| 2007 | S. Takeda, T. Nakai, Y. Wakai, F. Ikeda and K. Hatano | In vitro and in vivo activities of a new cephalosporin, FR264205, against Pseudomonas aeruginosa | 10.1128/AAC.00860-06 | NO | NO | IN VITRO STUDY |
| 2010 | G. H. Talbot | The antibiotic development pipeline for multidrug-resistant gram-negative bacilli: Current and future landscapes | 10.1086/655988 | NO | NO | REVIEW |
| 2019 | G. H. Talbot, A. Jezek, B. E. Murray, R. N. Jones, R. H. Ebright, G. J. Nau, K. A. Rodvold, J. G. Newland and H. W. Boucher | The infectious diseases society of America's 10 × '20 initiative (10 new systemic antibacterial agents US food and drug administration approved by 2020): Is 20 × '20 a possibility? | 10.1093/cid/ciz089 | NO | NO | OUT OF TOPIC |
| 2019 | P. D. Tamma and A. J. Hsu | Defining the role of novel β-lactam agents that target carbapenem-resistant gram-negative organisms | 10.1093/jpids/piz002 | NO | NO | REVIEW |
| 2017 | P. D. Tamma and J. Rodriguez-Bano | The Use of Noncarbapenem beta-Lactams for the Treatment of Extended-Spectrum beta-Lactamase Infections | 10.1093/cid/cix034 | NO | NO | REVIEW |
| 2016 | S. M. Tamma, A. J. Hsu and P. D. Tamma | Prescribing Ceftolozane/Tazobactam for Pediatric Patients: Current Status and Future Implications | 10.1007/s40272-015-0157-x | NO | NO | REVIEW |
| 2020 | K. Tan, J. Nguyen, K. Nguyen, H. K. Huse, P. H. Nieberg and A. Wong-Beringer | Prevalence of the carbapenem-heteroresistant phenotype among ESBL-producing Escherichia coli and Klebsiella pneumoniae clinical isolates | 10.1093/jac/dkaa048 | NO | NO | IN VITRO STUDY |
| 2019 | X. Tan and R. P. Moenster | Ceftolozane–tazobactam for the treatment of osteomyelitis caused by multidrug-resistant pathogens: a case series | 10.1177/2042098619862083 | NO | NO | CASE REPORT |
| 2014 | G. Tarchini and J. S. Solomkin | Non-inferiority designs and novel antimicrobials | 10.4155/cli.14.34 | NO | NO | NOT FOUND |
| 2015 | M. Tato, M. Garcia-Castillo, A. M. Bofarull and R. Canton | In vitro activity of ceftolozane/tazobactam against clinical isolates of Pseudomonas aeruginosa and Enterobacteriaceae recovered in Spanish medical centres: Results of the CENIT study | 10.1016/j.ijantimicag.2015.07.004 | NO | NO | IN VITRO STUDY |
| 2018 | K. H. M. E. Tehrani and N. I. Martin | β-lactam/β-lactamase inhibitor combinations: an update | 10.1039/c8md00342d | NO | NO | REVIEW |
| 2018 | M. Teleb, E. Soto-Ruiz, D. C. Dominguez and S. Antony | ESBL E coli and P. aeruginosa Resistance to Ceftolozane-Tazobactam in a Patient with a Liver Abscess. The Search for an Omnipotent Antibiotic Goes On! | 10.2174/1871526516666160713163238 | NO | NO | CASE REPORT |
| 2017 | J. Terracciano, E. G. Rhee and J. Walsh | Chemical Stability of Ceftolozane/Tazobactam in Polyvinylchloride Bags and Elastomeric Pumps | 10.1016/j.curtheres.2017.03.002 | NO | NO | PHARMACOLOGICAL STUDY |
| 2017 | A. K. Thabit, Y. Hamada and D. P. Nicolau | Physical compatibility of ceftolozane-tazobactam with selected i.v. drugs during simulated Y-site administration | 10.2146/ajhp150762 | NO | NO | PHARMACOLOGICAL STUDY |
| 2020 | R. Thakare, A. Dasgupta and S. Chopra | Imipenem/cilastatin sodium/relebactam fixed combination to treat urinary infections and complicated intra-abdominal bacterial infections | 10.1358/dot.2020.56.4.3075796 | NO | NO | REVIEW |
| 2019 | C. M. Thaulow, H. S. Blix, B. H. Eriksen, I. Ask, T. Å Myklebust and D. Berild | Using a period incidence survey to compare antibiotic use in children between a university hospital and a district hospital in a country with low antimicrobial resistance: A prospective observational study | 10.1136/bmjopen-2018-027836 | NO | NO | OUT OF TOPIC |
| 2009 | U. Theuretzbacher | Antibiotics: Derivative drugs, novel compounds and the need for effective resistance strategies | 10.2217/fmb.09.97 | NO | NO | REVIEW |
| 2009 | U. Theuretzbacher | Future antibiotics scenarios: is the tide starting to turn? | 10.1016/j.ijantimicag.2009.02.005 | NO | NO | REVIEW |
| 2011 | U. Theuretzbacher | Resistance drives antibacterial drug development | 10.1016/j.coph.2011.07.008 | NO | NO | REVIEW |
| 2012 | U. Theuretzbacher | Accelerating resistance, inadequate antibacterial drug pipelines and international responses | 10.1016/j.ijantimicag.2011.12.006 | NO | NO | REVIEW |
| 2019 | S. Thomas | Management of Resistant Gram-negative Infections; experience with the new agents from a tertiary referral center in the UK | 10.1016/j.jiph.2018.10.062 | NO | NO | ABSTRACT |
| 2015 | G. S. Tillotson and S. H. Zinner | Antibiotic Development and the Evolving Role of Pharmacodynamics - As Good as It Gets? | 10.1016/j.ebiom.2015.06.010 | NO | NO | REVIEW |
| 2017 | J. F. Timsit, W. Esaied, M. Neuville, L. Bouadma and B. Mourvllier | Update on ventilator-associated pneumonia | 10.12688/f1000research.12222.1 | YES | NO | REVIEW |
| 2017 | J. F. Timsit, B. Pilmis and J. R. Zahar | How Should We Treat Hospital-Acquired and Ventilator-Associated Pneumonia Caused by Extended-Spectrum β-Lactamase-Producing Enterobacteriaceae? | 10.1055/s-0037-1603112 | NO | NO | REVIEW |
| 2011 | E. Titelman, I. M. Karlsson, Y. Ge and C. G. Giske | In vitro activity of CXA-101 plus tazobactam (CXA-201) against CTX-M-14- and CTX-M-15-producing Escherichia coli and Klebsiella pneumoniae | 10.1016/j.diagmicrobio.2011.02.004 | NO | NO | IN VITRO STUDY |
| 2008 | A. Toda, H. Ohki, T. Yamanaka, K. Murano, S. Okuda, K. Kawabata, K. Hatano, K. Matsuda, K. Misumi, K. Itoh, K. Satoh and S. Inoue | Synthesis and SAR of novel parenteral anti-pseudomonal cephalosporins: Discovery of FR264205 | 10.1016/j.bmcl.2008.07.085 | NO | NO | REVIEW |
| 2019 | C. Toro Blanch, L. Gratacos Santanach, C. Diez Vallejo and R. Sacrest Guell | Hypokalaemia probably associated with ceftolozane/tazobactam treatment: Three case reports | 10.1016/j.eimc.2018.06.013 | NO | NO | CASE REPORT |
| 2018 | A. Torres, M. S. Niederman, J. Chastre, S. Ewig, P. Fernandez-Vandellos, H. Hanberger, M. Kollef, G. L. Bassi, C. M. Luna, I. Martin-Loeches, J. A. Paiva, R. C. Read, D. Rigau, J. F. Timsit, T. Welte and R. Wunderink | Summary of the international clinical guidelines for the management of hospital-acquired and ventilator-acquired pneumonia | 10.1183/23120541.00028-2018 | NO | NO | REVIEW |
| 2015 | K. A. Toussaint and J. C. Gallagher | β-Lactam/β-Lactamase Inhibitor Combinations: From Then to Now | 10.1177/1060028014556652 | NO | NO | REVIEW |
| 2017 | T. T. Tran, L. Diaz, H. Abodakpi, J. Ardila, E. De La Cadena, R. Rios, W. Miller, L. Rivas, A. Dinh, P. Porras, D. Panesso, V. Tam, J. M. Munita and C. Arias | Ceftolozane-tazobactam resistance in multidrug-resistant pseudomonas aeruginosa isolates not associated with AMPC activity | 10.1093/ofid/ofx163.175 | NO | NO | ABSTRACT |
| 2019 | M. J. Trisler, P. Tamma and E. Avdic | Comparison of outcomes between patients with and without cystic fibrosis treated with ceftolozane-tazobactam for pseudomonas aeruginosa infections | 10.1093/ofid/ofz360.1931 | YES | NO | ABSTRACT |
| 2017 | T. Trupka, K. Fisher, S. T. Micek, P. Juang and M. H. Kollef | PHARMACOLOGICAL basis of β-lactamase inhibitor therapeutics: Tazobactam in combination with ceftolozane | 10.1186/s13054-017-1772-4 | NO | NO | PHARMACOLOGICAL STUDY |
| 2020 | L. Tselepis, G. W. Langley, A. F. Aboklaish, E. Widlake, D. E. Jackson, Trwcj Schofield, J. Brem and J. M. Tyrrell | In vitro efficacy of Imipenem-Relebactam and Cefepime-AAI101 against a global collection of ESBL-positive and carbapenemase-producing Enterobacteriaceae | 10.1016/j.ijantimicag.2020.105925 | NO | NO | IN VITRO STUDY |
| 2017 | M. Tsuji, M. Hackel, R. Echols, Y. Yamano and D. Sahm | In vitro activity of cefiderocol against globally collected carbapenem-resistant gram-negative bacteria isolated from urinary track source: Sidero-cr-2014/2016 | 10.1093/ofid/ofx163.895 | NO | NO | IN VITRO STUDY |
| 2017 | M. Tsuji, M. Hackel, R. Echols, Y. Yamano and D. Sahm | In vitro activity of cefiderocol against gram-negative clinical isolates collected from urinary track source: Sidero-WT-2014/Sidero-WT-2015 | 10.1093/ofid/ofx163.925 | NO | NO | IN VITRO STUDY |
| 2018 | M. Tsuji, M. Hackel, R. Echols, Y. Yamano and D. Sahm | Global surveillance of cefiderocol against gram-negative clinical strains collected in north america: Sidero-WT-2015 | 10.1093/ofid/ofy210.1180 | NO | NO | ABSTRACT |
| 2017 | P. M. Tulkens | Retrospective Evaluation of the Use of Ceftolozane/Tazobactam at a Large Academic Medical Center: What Did We Learn? | 10.1097/IPC.0000000000000573 | NO | NO | CASE SERIES |
| 2015 | C. Tzialla, A. Borghesi, M. Pozzi and M. Stronati | Neonatal infections due to multi-resistant strains: Epidemiology, current treatment, emerging therapeutic approaches and prevention | 10.1016/j.cca.2015.02.038 | NO | NO | REVIEW |
| 2019 | E. R. Ulloa, N. Dillon, H. Tsunemoto, J. Pogliano, G. Sakoulas and V. Nizet | Avibactam sensitizes carbapenem-resistant NDM-1-producing klebsiella pneumoniae to innate immune clearance | 10.1093/infdis/jiz128 | NO | NO | IN VITRO STUDY |
| 2017 | M. Vacas-Córdoba, C. Cardozo-Espinola, P. Puerta-Alcalde, C. Cilloniz, A. Torres and C. García-Vidal | Empirical treatment of adults with hospital-acquired pneumonia: Lights and shadows of the 2016 clinical practice ATS/IDSA guidelines |  | NO | NO | REVIEW |
| 2018 | T. Valentin, G. Feierl, L. Masoud-Landgraf, P. Kohek, J. Luxner and G. Zarfel | Proteus mirabilis harboring carbapenemase NDM-5 and ESBL VEB-6 detected in Austria | 10.1016/j.diagmicrobio.2018.02.009 | NO | NO | CASE REPORT |
| 2018 | A. Van Dalem, M. Herpol, F. Echahidi, C. Peeters, I. Wybo, E. De Wachter, P. Vandamme and D. Pierard | In Vitro Susceptibility of Burkholderia cepacia Complex Isolated from Cystic Fibrosis Patients to Ceftazidime-Avibactam and Ceftolozane-Tazobactam | 10.1128/aac.00590-18 | NO | NO | IN VITRO STUDY |
| 2016 | D. van Duin and R. A. Bonomo | Ceftazidime/Avibactam and Ceftolozane/Tazobactam: Second-generation β-Lactam/β-Lactamase Inhibitor Combinations | 10.1093/cid/ciw243 | YES | NO | REVIEW |
| 2013 | B. Van Scoy, R. E. Mendes, M. Castanheira, J. McCauley, S. M. Bhavnani, A. Forrest, R. N. Jones, O. O. Okusanya, L. V. Friedrich, J. Steenbergen and P. G. Ambrose | Relationship between ceftolozane-tazobactam exposure and drug resistance amplification in a hollow-fiber infection model | 10.1128/AAC.00461-13 | NO | NO | IN VITRO STUDY |
| 2013 | B. VanScoy, R. E. Mendes, J. McCauley, S. M. Bhavnani, C. C. Bulik, O. O. Okusanya, A. Forrest, R. N. Jones, L. V. Friedrich, J. N. Steenbergen and P. G. Ambrose | Pharmacological basis of β-lactamase inhibitor therapeutics: Tazobactam in combination with ceftolozane | 10.1128/AAC.00656-13 | NO | NO | PHARMACOLOGICAL STUDY |
| 2013 | B. VanScoy, R. E. Mendes, A. M. Nicasio, M. Castanheira, C. C. Bulik, O. O. Okusanya, S. M. Bhavnani, A. Forrest, R. N. Jones, L. V. Friedrich, J. N. Steenbergen and P. G. Ambrose | Pharmacokinetics-pharmacodynamics of tazobactam in combination with ceftolozane in an in vitro infection model | 10.1128/AAC.02513-12 | NO | NO | PHARMACOLOGICAL STUDY |
| 2014 | B. D. VanScoy, R. E. Mendes, M. Castanheira, J. McCauley, S. M. Bhavnani, R. N. Jones, L. V. Friedrich, J. N. Steenbergen and P. G. Ambrose | Relationship between ceftolozane-tazobactam exposure and selection for Pseudomonas aeruginosa resistance in a hollow-fiber infection model | 10.1128/AAC.02310-13 | NO | NO | IN VITRO STUDY |
| 2017 | B. D. VanScoy, D. Tenero, S. Turner, D. M. Livermore, J. McCauley, H. Conde, S. M. Bhavnani, C. M. Rubino and P. G. Ambrose | Pharmacokinetics-pharmacodynamics of tazobactam in combination with cefepime in an in vitro infection model | 10.1128/AAC.01052-17 | NO | NO | PHARMACOLOGICAL STUDY |
| 2018 | K. Z. Vardakas, G. L. Voulgaris, A. Maliaros, G. Samonis and M. E. Falagas | Prolonged versus short-term intravenous infusion of antipseudomonal β-lactams for patients with sepsis: a systematic review and meta-analysis of randomised trials | 10.1016/S1473-3099(17)30615-1 | NO | NO | SYSTEMATIC REVIEW |
| 2018 | B. Veeraraghavan, A. Pragasam, Y. Bakthavatchalam, S. Anandan, V. Ramasubramanian, S. Swaminathan, R. Gopalakrishnan, R. Soman, O. Abraham, V. Ohri and K. Walia | Newer β-Lactam/β-Lactamase inhibitor for multidrug-resistant gram-negative infections: Challenges, implications and surveillance strategy for India | 10.4103/ijmm.IJMM_18_326 | NO | NO | REVIEW |
| 2016 | A. L. Velez Perez, S. M. Schmidt-Malan, P. C. Kohner, M. J. Karau, K. E. Greenwood-Quaintance and R. Patel | In vitro activity of ceftolozane/tazobactam against clinical isolates of Pseudomonas aeruginosa in the planktonic and biofilm states | 10.1016/j.diagmicrobio.2016.02.014 | NO | NO | IN VITRO STUDY |
| 2020 | A. Vena, D. R. Giacobbe, N. Castaldo, A. Cattelan, C. Mussini, R. Luzzati, F. G. De Rosa, F. Del Puente, C. M. Mastroianni, A. Cascio, S. Carbonara, A. Capone, S. Boni, C. Sepulcri, M. Meschiari, F. Raumer, A. Oliva, S. Corcione and M. Bassetti | Clinical experience with ceftazidime-avibactam for the treatment of infections due to multidrug-resistant gram-negative bacteria other than carbapenem-resistant Enterobacterales | 10.3390/antibiotics9020071 | NO | NO | CASE SERIES |
| 2020 | A. Vena, D. R. Giacobbe, C. Mussini, A. Cattelan and M. Bassetti | Clinical Efficacy of Ceftolozane-tazobactam Versus Other Active Agents for the Treatment of Bacteremia and Nosocomial Pneumonia Due to Drug resistant P. aeruginosa | 10.1093/cid/ciaa003 | NO | NO | LETTER |
| 2008 | P. I. Vergidis and M. E. Falagas | Multidrug-resistant Gram-negative bacterial infections: The emerging threat and potential novel treatment options |  | NO | NO | REVIEW |
| 2020 | M. P. Veve, E. D. McCurry, G. E. Cooksey and M. A. Shorman | Epidemiology and outcomes of non-HACEK infective endocarditis in the southeast United States | 10.1371/journal.pone.0230199 | NO | NO | NOT COMPARABLE DATA |
| 2019 | B. Viala, F. Z. Zaidi, M. Bastide, Y. Dumont, V. Le Moing, H. Jean-Pierre and S. Godreuil | Assessment of the in Vitro Activities of Ceftolozane/Tazobactam and Ceftazidime/Avibactam in a Collection of Beta-Lactam-Resistant Enterobacteriaceae and Pseudomonas aeruginosa Clinical Isolates at Montpellier University Hospital, France | 10.1089/mdr.2018.0439 | NO | NO | IN VITRO STUDY |
| 2015 | P. Viale, M. Giannella, M. Bartoletti, S. Tedeschi and R. Lewis | Considerations About Antimicrobial Stewardship in Settings with Epidemic Extended-Spectrum β-Lactamase-Producing or Carbapenem-Resistant Enterobacteriaceae | 10.1007/s40121-015-0081-y | NO | NO | REVIEW |
| 2016 | S. B. Vickery, D. McClain and K. A. Wargo | Successful Use of Ceftolozane-Tazobactam to Treat a Pulmonary Exacerbation of Cystic Fibrosis Caused by Multidrug-Resistant Pseudomonas aeruginosa | 10.1002/phar.1825 | NO | NO | CASE REPORT |
| 2019 | J. Villa, O. Carretero, E. Viedma, J. Lora-Tamayo, J. Mingorance and F. Chaves | Emergence of NDM-7-producing multi-drug-resistant Enterobacter hormaechei sequence type ST-78 in Spain: a high-risk international clone | 10.1016/j.ijantimicag.2018.11.009 | NO | NO | CASE REPORT |
| 2018 | M. V. Villegas and S. Lyon | Gram-negative infections: Evolving treatments with expanding options | 10.4155/fsoa-2018-0071 | NO | NO | REVIEW |
| 2016 | J. L. Vincent, M. Bassetti, B. François, G. Karam, J. Chastre, A. Torres, J. A. Roberts, F. S. Taccone, J. Rello, T. Calandra, D. De Backer, T. Welte and M. Antonelli | Advances in antibiotic therapy in the critically ill | 10.1186/s13054-016-1285-6 | NO | NO | REVIEW |
| 2019 | G. L. Voulgaris, M. L. Voulgari and M. E. Falagas | Developments on antibiotics for multidrug resistant bacterial Gram-negative infections | 10.1080/14787210.2019.1610392 | NO | NO | REVIEW |
| 2019 | M. Vurchio, A. Cavuoto, V. De Palo, M. Gaglio, L. Mazzilli, M. Cassatella, G. Garribba and M. Debitonto | Successful treatment with cytosorb in two patients with septic shock: A single centre experience | 10.1159/000500179 | NO | NO | ABSTRACT |
| 2016 | F. M. Wagenlehner and J. F. Alidjanov | Efficacy, pharmacokinetic and pharmacodynamic profile of ceftolozane + tazobactam in the treatment of complicated urinary tract infections | 10.1080/17425255.2016.1201065 | NO | NO | REVIEW |
| 2015 | F. M. Wagenlehner, O. Umeh and R. O. Darouiche | Ceftolozane-tazobactam versus levofloxacin in urinary tract infection - Authors' reply | 10.1016/S0140-6736(15)00263-9 | NO | NO | LETTER |
| 2019 | F. M. E. Wagenlehner and K. G. Naber | Cefiderocol for treatment of complicated urinary tract infections | 10.1016/S1473-3099(18)30722-9 | NO | NO | LETTER |
| 2018 | A. Walkty, H. Adam, M. Baxter, P. Lagace-Wiens, J. A. Karlowsky, D. J. Hoban and G. G. Zhanel | In vitro activity of ceftolozane/tazobactam versus antimicrobial non-susceptible Pseudomonas aeruginosa clinical isolates including MDR and XDR isolates obtained from across Canada as part of the CANWARD study, 2008-16 | 10.1093/jac/dkx468 | NO | NO | IN VITRO STUDY |
| 2017 | A. Walkty, H. J. Adam, M. Baxter, P. Lagace-Wiens, J. Karlowsky, D. Hoban and G. Zhanel | In vitro activity of ceftolozane-tazobactam vs. antimicrobial non-susceptible pseudomonas aeruginosa clinical isolates obtained from across Canada as part of the canward study, 2008-2016 | 10.1093/ofid/ofx163.914 | NO | NO | IN VITRO STUDY |
| 2018 | A. Walkty, H. J. Adam, M. Baxter, P. Lagace-Wiens, J. Karlowsky, D. Hoban and G. Zhanel | In vitro activity of ceftolozane-tazobactam in comparison with ceftazidime-avibactam vs. Antimicrobial Non-Susceptible pseudomonas aeruginosa clinical isolates, including multidrug-resistant and extensively drug-resistant subsets: CANWARD, 2007-2017 | 10.1093/ofid/ofy210.2036 | NO | NO | IN VITRO STUDY |
| 2017 | A. Walkty, M. Baxter, H. J. Adam, P. Lagace-Wiens, J. Karlowsky and G. Zhanel | In vitro activity of newer antimicrobials and relevant comparators vs. 349 Stenotrophomonas maltophilia Clinical Isolates Obtained from Patients in Canadian Hospitals (CANWARD, 2011-2016) | 10.1093/ofid/ofx163.899 | NO | NO | IN VITRO STUDY |
| 2013 | A. Walkty, J. A. Karlowsky, H. Adam, M. Baxter, P. Lagace-Wiens, D. J. Hoban and G. G. Zhanel | In vitro activity of ceftolozane-tazobactam against Pseudomonas aeruginosa isolates obtained from patients in Canadian hospitals in the CANWARD study, 2007 to 2012 | 10.1128/aac.01404-13 | NO | NO | IN VITRO STUDY |
| 2019 | T. Z. Wang, R. P. L. Kodiyanplakkal and D. P. Calfee | Antimicrobial resistance in nephrology | 10.1038/s41581-019-0150-7 | NO | NO | REVIEW |
| 2017 | R. R. Watkins and S. Deresinski | Using β-lactam/β-lactamase inhibitors for infections due to extended-spectrum β-lactamase-producing Enterobacteriaceae to slow the emergence of carbapenem-resistant Enterobacteriaceae | 10.1080/14787210.2017.1380519 | NO | NO | EDITORIAL |
| 2013 | R. R. Watkins, K. M. Papp-Wallace, S. M. Drawz and R. A. Bonomo | Novel β-lactamase inhibitors: A therapeutic hope against the scourge of multidrug resistance | 10.3389/fmicb.2013.00392 | NO | NO | REVIEW |
| 2017 | E. Weiss, W. Essaied, C. Adrie, J. R. Zahar and J. F. Timsit | Treatment of severe hospital-acquired and ventilator-associated pneumonia: A systematic review of inclusion and judgment criteria used in randomized controlled trials | 10.1186/s13054-017-1755-5 | NO | NO | SYSTEMATIC REVIEW |
| 2017 | E. Wenzler, K. L. Bunnell, S. C. Bleasdale, S. Benken, L. H. Danziger and K. A. Rodvold | Pharmacokinetics and dialytic clearance of ceftazidime-avibactam in a critically ill patient on continuous venovenous hemofiltration | 10.1128/AAC.00464-17 | NO | NO | PHARMACOLOGICAL STUDY |
| 2015 | K. Wheeler | Fda approves new antibiotic combo for two complicated infections |  | NO | NO | OUT OF TOPIC |
| 2017 | Y. M. Wi, K. E. Greenwood-Quaintance, K. S. Ko, K. T. Kwon, S. I. Jung, Y. S. Kim, S. W. Kim, J. S. Yeom, K. R. Peck, J. H. Song and R. Patel | Activity of ceftolozane/tazobactam against carbapenem-resistant Pseudomonas aeruginosa |  | NO | NO | IN VITRO STUDY |
| 2018 | P. L. Woerther, R. Lepeule, C. Burdet, J. W. Decousser, É Ruppé and F. Barbier | Carbapenems and alternative β-lactams for the treatment of infections due to extended-spectrum β-lactamase-producing Enterobacteriaceae: What impact on intestinal colonisation resistance? | 10.1016/j.ijantimicag.2018.08.026 | NO | NO | REVIEW |
| 2019 | J. B. Wood, L. B. Cravens and C. B. Creech | Advances in pediatric antimicrobial agents development | 10.1097/MOP.0000000000000713 | NO | NO | REVIEW |
| 2014 | M. Wooley, B. Miller, G. Krishna, E. Hershberger and G. Chandorkar | Impact of renal function on the pharmacokinetics and safety of ceftolozane-tazobactam | 10.1128/AAC.02151-13 | NO | NO | PHARMACOLOGICAL STUDY |
| 2016 | S. A. Woon and D. Fisher | Antimicrobial agents - optimising the ecological balance | 10.1186/s12916-016-0661-z | NO | NO | REVIEW |
| 2016 | G. D. Wright | Antibiotic Adjuvants: Rescuing Antibiotics from Resistance | 10.1016/j.tim.2016.06.009 | NO | NO | REVIEW |
| 2017 | H. Wright, R. A. Bonomo and D. L. Paterson | New agents for the treatment of infections with Gram-negative bacteria: restoring the miracle or false dawn? | 10.1016/j.cmi.2017.09.001 | NO | NO | REVIEW |
| 2020 | J. Y. Wu, P. Srinivas and J. M. Pogue | Cefiderocol: A Novel Agent for the Management of Multidrug-Resistant Gram-Negative Organisms | 10.1007/s40121-020-00286-6 | NO | NO | REVIEW |
| 2019 | R. G. Wunderink, C. Bruno, I. Martin-Loeches, M. Kollef, J. F. Timsit, B. Yu, J. A. Huntington, L. Li, E. Jensen, D. Wolf, J. R. Butterton and E. G. Rhee | Impact of prior and concomitant antibacterial therapy on outcomes in the ASPECT-NP randomized, controlled trial of ceftolozane/tazobactam (C/T) vs. meropenem (MEM) in patients with ventilated nosocomial pneumonia (NP) | 10.1093/ofid/ofz360.1904 | YES | NO | ABSTRACT |
| 2017 | A. J. Xiao, L. Caro, M. W. Popejoy, J. A. Huntington and R. Kullar | PK/PD Target Attainment With Ceftolozane/Tazobactam Using Monte Carlo Simulation in Patients With Various Degrees of Renal Function, Including Augmented Renal Clearance and End-Stage Renal Disease | 10.1007/s40121-016-0143-9 | NO | NO | PHARMACOLOGICAL STUDY |
| 2018 | A. J. Xiao, J. A. Huntington, J. Long and L. Caro | Ceftolozane/tazobactam dose regimens in severely/morbidly obese patients with complicated intra-abdominal infection or complicated urinary tract infection | 10.1016/j.ijantimicag.2018.03.004 | NO | NO | PHARMACOLOGICAL STUDY |
| 2016 | A. J. Xiao, B. W. Miller, J. A. Huntington and D. P. Nicolau | Ceftolozane/tazobactam pharmacokinetic/pharmacodynamic-derived dose justification for phase 3 studies in patients with nosocomial pneumonia | 10.1002/jcph.566 | NO | NO | PHARMACOLOGICAL STUDY |
| 2017 | M. Xipell, M. Bodro, F. Marco, J. A. Martinez and A. Soriano | Successful treatment of three severe MDR or XDR Pseudomonas aeruginosa infections with ceftolozane/tazobactam | 10.2217/fmb-2017-0018 | NO | NO | CASE REPORT |
| 2019 | A. Xu, S. Elsayed and R. Dhami | A retrospective case series assessing ceftolozane/tazobactam use at a large academic centre | <http://www.embase.com/search/results?subaction=viewrecord&from=export&id=L627350101> | NO | NO | CONFERENCE PROCEEDINGS |
| 2017 | S. Yamamoto | Antibiotic resistance a major problem for management of urogenital infection |  | NO | NO | REVIEW |
| 2019 | Y. Yamano, M. Tsuji and R. Echols | Synergistic effect of cefiderocol combined with other antibiotics against cefiderocol high mic isolates from the multi-national sidero-wt studies | 10.1093/ofid/ofz360.791 | NO | NO | ABSTRACT |
| 2018 | Y. Yamano, M. Tsuji, R. Echols, M. Hackel and D. Sahm | In vitro activity of cefiderocol against gram-negative clinical isolates from respiratory specimens: Sidero-WT-2014 |  | NO | NO | IN VITRO STUDY |
| 2019 | H. Yang, A. Motos, G. L. Bassi, E. A. Xiol, F. Pagliara, T. Senussi, F. A. Idone, J. Bobi, R. Cabrera, L. Fernandez-Barat, M. Yang, D. Nicolau, P. Pelosi, M. Antonelli and A. Torres | Efficacy of Ceftolozane/Tazobactam in Comparison with Piperacillin/Tazobactam in Severe Pseudomonas aeruginosa Pneumonia Swine Model | 10.1183/13993003.congress-2019.PA4562 | NO | NO | PRECLINICAL STUDY |
| 2020 | R. S. Yang, L. K. Zhang, R. Helmy, T. Andreani, Z. Liu and H. Sheng | Development of a highly efficient decontamination approach for ceftolozane in the pharmaceutical manufacturing environment | 10.1016/j.jpba.2019.112846 | NO | NO | OUT OF TOPIC |
| 2018 | S. Yang, P. Hemarajata, L. Shevy, M. Maciariello, K. Culbreath, K. Bush and R. Humphries | Unusual carbapenem resistant but ceftriaxone and cefepime susceptible Klebsiella oxytoca isolated from a blood culture: Case report and whole-genome sequencing investigation | 10.1016/j.idcr.2017.11.007 | NO | NO | CASE REPORT |
| 2019 | M. E. Yarrington, D. J. Anderson, E. Dodds Ashley, T. Jones, A. Davis, M. Johnson, Y. Lokhnygina, D. J. Sexton and R. W. Moehring | Impact of FDA black box warning on fluoroquinolone and alternative antibiotic use in southeastern US hospitals | 10.1017/ice.2019.247 | NO | NO | OUT OF TOPIC |
| 2019 | D. Yin, S. Wu, Y. Yang, Q. Shi, D. Dong, D. Zhu and F. Hu | Results from the China Antimicrobial Surveillance Network (CHINET) in 2017 of the In Vitro Activities of Ceftazidime-Avibactam and Ceftolozane-Tazobactam against Clinical Isolates of Enterobacteriaceae and Pseudomonas aeruginosa | 10.1128/AAC.02431-18 | NO | NO | IN VITRO STUDY |
| 2018 | B. Yu, A. Adedoyin, E. Hershberger, L. Caro, A. Xiao, E. G. Rhee and J. A. Huntington | Safety, Tolerability, and Pharmacokinetics of 3 g of Ceftolozane/Tazobactam in Healthy Adults: a Randomized, Placebo-Controlled, Multiple-Dose Study | 10.1002/cpdd.429 | NO | NO | PHARMACOLOGICAL STUDY |
| 2019 | G. Yu, H. H. Zhou, Q. S. Zheng and G. F. Li | Interethnic scaling of fraction unbound of a drug in plasma and volume of distribution: an analysis of extrapolation from Caucasians to Chinese | 10.1007/s00228-018-02610-z | NO | NO | PHARMACOLOGICAL STUDY |
| 2010 | L. Zamorano, C. Juan, A. Fernandez-Olmos, Y. Ge, R. Canton and A. Oliver | Activity of the new cephalosporin CXA-101 (FR264205) against Pseudomonas aeruginosa isolates from chronically-infected cystic fibrosis patients | 10.1111/j.1469-0691.2009.03130.x | NO | NO | IN VITRO STUDY |
| 2019 | R. Zamudio, K. Hijazi, C. Joshi, E. Aitken, M. R. Oggioni and I. M. Gould | Phylogenetic analysis of resistance to ceftazidime/avibactam, ceftolozane/tazobactam and carbapenems in piperacillin/tazobactam-resistant Pseudomonas aeruginosa from cystic fibrosis patients | 10.1016/j.ijantimicag.2019.02.022 | NO | NO | IN VITRO STUDY |
| 2019 | A. Zavala, P. Retailleau, E. Elisee, B. I. Iorga and T. Naas | Genetic, Biochemical, and Structural Characterization of CMY-136 beta-Lactamase, a Peculiar CMY-2 Variant | 10.1021/acsinfecdis.8b00240 | NO | NO | CASE REPORT |
| 2017 | H. Zemlickova, V. Jakubu and M. Fridrichova | [Antimicrobial activity of ceftolozane/tazobactam against Enterobacteriaceae and Pseudomonas aeruginosa in the Czech Republic in 2016] |  | NO | NO | IN VITRO STUDY |
| 2019 | G. G. Zhanel, H. J. Adam, M. R. Baxter, J. Fuller, K. A. Nichol, A. J. Denisuik, A. R. Golden, R. Hink, P. R. S. Lagace-Wiens, A. Walkty, M. R. Mulvey, F. Schweizer, D. Bay, D. J. Hoban and J. A. Karlowsky | 42936 pathogens from Canadian hospitals: 10 years of results (2007-16) from the CANWARD surveillance study | 10.1093/jac/dkz283 | NO | NO | IN VITRO STUDY |
| 2014 | G. G. Zhanel, P. Chung, H. Adam, S. Zelenitsky, A. Denisuik, F. Schweizer, P. R. Lagace-Wiens, E. Rubinstein, A. S. Gin, A. Walkty, D. J. Hoban, J. P. Lynch, 3rd and J. A. Karlowsky | Ceftolozane/tazobactam: a novel cephalosporin/beta-lactamase inhibitor combination with activity against multidrug-resistant gram-negative bacilli | 10.1007/s40265-013-0168-2 | NO | NO | REVIEW |
| 2018 | G. G. Zhanel, M. A. Zhanel and J. A. Karlowsky | Intravenous fosfomycin: An assessment of its potential for use in the treatment of systemic infections in Canada | 10.1155/2018/8912039 | NO | NO | REVIEW |
| 2019 | J. Zhang, R. Dillon, E. McCann, M. Lorenzi, E. Sarpong, S. Mehta and L. Puzniak | PIN1 SYSTEMATIC REVIEW AND NETWORK META-ANALYSIS COMPARING THE EFFICACY AND SAFETY OF CEFTOLOZANE/TAZOBACTAM AND COMPARATORS IN THE TREATMENT OF HOSPITAL-ACQUIRED BACTERIAL PNEUMONIA AND VENTILATOR-ASSOCIATED BACTERIAL PNEUMONIA | 10.1016/j.jval.2019.04.875 | NO | NO | SYSTEMATIC REVIEW |
| 2019 | J. Zhang, A. Khalifeh, R. Santini-Dominguez, R. N. Barth, D. Bruno, S. Desikan, A. Gupta and S. Toursavadkohi | Endovascular Reconstruction of the Hepatic Arterial System for the Management of Mycotic Pseudoaneurysm in a Liver Transplant Patient | 10.1016/j.avsg.2019.05.060 | NO | NO | CASE REPORT |
| 2020 | Z. Zhang, Y. Patel, J. Fiedler-Kelly, H. Feng, M. Johnson, E. Rhee, J. Huntington, M. Rizk and W. Gao | Ceftolozane-tazobactam dose selection for clinical evaluation in pediatric patients with nosocomial pneumonia using a Bayesian approach |  | NO | NO | PHARMACOLOGICAL STUDY |
| 2019 | A. Zikri and K. El Masri | Use of Ceftolozane/tazobactam for the Treatment of Multidrug-resistant Pseudomonas aeruginosa Pneumonia in a Pediatric Patient with Combined Immunodeficiency (CID): A Case Report from a Tertiary Hospital in Saudi Arabia | 10.3390/antibiotics8020067 | NO | NO | CASE REPORT |
| 2019 | M. D. Zilberberg, B. H. Nathanson, K. Ditch, K. Lawrence, M. Olesky and A. F. Shorr | Carbapenem treatment and outcomes among patients with culture-positive complicated intra-abdominal infections in US hospitals: A retrospective cohort study | 10.1093/ofid/ofz504 | NO | NO | AGGREGATED DATA |
| 2017 | J. T. Zobell, K. L. Epps and D. C. Young | Optimization of anti-pseudomonal antibiotics for cystic fibrosis pulmonary exacerbations: II. Cephalosporins and penicillins update | 10.1002/ppul.23713 | YES | NO | LETTER |
| 2020 | A. M. Alnimr and A. M. Alamri | Antimicrobial activity of cephalosporin-beta-lactamase inhibitor combinations against drug-susceptible and drug-resistant Pseudomonas aeruginosa strains | 10.1016/j.jtumed.2020.04.004 | NO | NO | IN VITRO STUDY |
| 2020 | J. Álvarez Otero, J. L. Lamas Ferreiro, A. Sanjurjo Rivo and J. de la Fuente Aguado | Outpatient Parenteral Antimicrobial Therapy With Ceftolozane/Tazobactam via Continuous Infusion for Multidrug-Resistant Pseudomonas aeruginosa Osteomyelitis | 10.1093/ofid/ofaa409 | NO | NO | CASE REPORT |
| 2020 | G. F. Araj, D. M. Berjawi, U. Musharrafieh and N. K. El Beayni | Activity of ceftolozane/tazobactam against commonly encountered antimicrobial resistant Gram-negative bacteria in Lebanon | 10.3855/jidc.12368 | NO | NO | IN VITRO STUDY |
| 2020 | J. Arca-Suárez, J. C. Vázquez-Ucha, P. A. Fraile-Ribot, E. Lence, G. Cabot, M. Martínez-Guitián, C. Lasarte-Monterrubio, M. Rodríguez-Iglesias, A. Beceiro, C. González-Bello, F. Galán-Sánchez, A. Oliver and G. Bou | Molecular and biochemical insights into the in vivo evolution of AmpC-mediated resistance to ceftolozane/tazobactam during treatment of an MDR Pseudomonas aeruginosa infection | 10.1093/jac/dkaa291 | NO | NO | IN VITRO STUDY |
| 2020 | A. C. Arrieta, J. Y. Ang, Z. Zhang, K. B. Larson, B. Yu, M. G. Johnson, E. G. Rhee, E. H. Feng and M. L. Rizk | Plasma pharmacokinetics of ceftolozane/tazobactam in pediatric patients with cystic fibrosis | 10.1002/ppul.24815 | NO | NO | PHARMACOLOGICAL STUDY |
| 2020 | A. Artese, V. Svicher, G. Costa, R. Salpini, V. C. Di Maio, M. Alkhatib, F. A. Ambrosio, M. M. Santoro, Y. G. Assaraf, S. Alcaro and F. Ceccherini-Silberstein | Current status of antivirals and druggable targets of SARS CoV-2 and other human pathogenic coronaviruses | 10.1016/j.drup.2020.100721 | NO | NO | OUT OF TOPIC |
| 2020 | M. Bassetti, D. R. Giacobbe, C. Robba, P. Pelosi and A. Vena | Treatment of extended-spectrum β-lactamases infections: what is the current role of new β-lactams/β-lactamase inhibitors? | 10.1097/qco.0000000000000685 | NO | NO | REVIEW |
| 2020 | E. M. Beirão, S. D. S. Rodrigues, T. K. Andrade, F. B. Serra, M. D. N. Paula, T. J. B. Polis and A. C. Gales | Activity of ceftolozane-tazobactam and comparators against gram-negative bacilli: Results from the study for monitoring antimicrobial resistance trends (SMART - Brazil; 2016-2017) | 10.1016/j.bjid.2020.05.010 | NO | NO | IN VITRO STUDY |
| 2020 | S. S. Bhagwat, P. Hariharan, P. R. Joshi, S. R. Palwe, R. Shrivastava, M. V. Patel, N. K. Devanga Ragupathi, Y. D. Bakthavatchalam, M. S. Ramesh, R. Soman and B. Veeraraghavan | Activity of cefepime/zidebactam against MDR Escherichia coli isolates harbouring a novel mechanism of resistance based on four-amino-acid inserts in PBP3 | 10.1093/jac/dkaa353 | NO | NO | IN VITRO STUDY |
| 2020 | K. C. Blomquist and D. E. Nix | A Critical Evaluation of Newer β-Lactam Antibiotics for Treatment of Pseudomonas aeruginosa Infections | 10.1177/1060028020974003 | NO | NO | REVIEW |
| 2020 | J. Canovas, G. Petitjean, F. Chau, A. Le Monnier, B. Fantin and A. Lefort | Expression of CTX-M-15 limits the efficacy of ceftolozane/tazobactam against Escherichia coli in a high-inoculum murine peritonitis model | 10.1016/j.cmi.2020.06.032 | NO | NO | IN VITRO STUDY |
| 2020 | D. Carr, Z. Zhang, Q. Si, F. Racine, J. C. Xiao, R. Katwaru, M. K. Wismer, M. G. Johnson, H. P. Feng, K. Young, M. L. Rizk and M. Motyl | In Vitro Hollow-Fiber Studies Assessing Antibacterial Activity of Ceftolozane/Tazobactam Against Multidrug-Resistant Pseudomonas Aeruginosa | 10.1093/ofid/ofaa469 | NO | NO | IN VITRO STUDY |
| 2020 | J. M. Cottreau and A. B. Christensen | Newly Approved Antimicrobials | 10.1097/nor.0000000000000633 | NO | NO | REVIEW |
| 2020 | R. Cultrera, M. Libanore, A. Barozzi, E. d'Anchera, L. Romanini, F. Fabbian, F. De Motoli, B. Quarta, A. Stefanati, N. Bolognesi and G. Gabutti | Ceftolozane/Tazobactam and Ceftazidime/Avibactam for Multidrug-Resistant Gram-Negative Infections in Immunocompetent Patients: A Single-Center Retrospective Study | 10.3390/antibiotics9100640 | YES | NO | AGGREGATED DATA |
| 2020 | R. S. R. da Cunha, E. Carniel, G. A. Narvaez, C. G. Dias and L. R. R. Perez | Impact of the blue-carba rapid test for carbapenemase detection on turnaround time for an early therapy against Pseudomonas aeruginosa | 10.1016/j.ajic.2020.08.018 | YES | NO | IN VITRO STUDY |
| 2020 | D. Fournier, R. Carrière, M. Bour, E. Grisot, P. Triponney, C. Muller, J. Lemoine, K. Jeannot and P. Plésiat | Mechanisms of Resistance to Ceftolozane/Tazobactam in Pseudomonas aeruginosa: Results of the GERPA Multicenter Study | 10.1128/aac.01117-20 | NO | NO | IN VITRO STUDY |
| 2020 | I. Galani, V. Papoutsaki, I. Karantani, I. Karaiskos, L. Galani, P. Adamou, I. Deliolanis, A. Kodonaki, E. Papadogeorgaki, M. Markopoulou, S. Maraki, M. Damala, E. Prifti, E. Vagiakou, E. Petinaki, K. Fountoulis, S. Tsiplakou, H. Kirikou, M. Souli, A. Antoniadou and H. Giamarellou | In vitro activity of ceftolozane/tazobactam alone and in combination with amikacin against MDR/XDR Pseudomonas aeruginosa isolates from Greece | 10.1093/jac/dkaa160 | NO | NO | IN VITRO STUDY |
| 2020 | S. Garazzino, E. Altieri, E. Silvestro, G. Pruccoli, C. Scolfaro and E. Bignamini | Ceftolozane/Tazobactam for Treating Children With Exacerbations of Cystic Fibrosis Due to Pseudomonas aeruginosa: A Review of Available Data | 10.3389/fped.2020.00173 | NO | NO | REVIEW |
| 2020 | M. Gatti, M. Giannella, E. Raschi, P. Viale and F. De Ponti | Ceftolozane/tazobactam exposure in critically ill patients undergoing continuous renal replacement therapy: a PK/PD approach to tailor dosing | 10.1093/jac/dkaa416 | NO | NO | PHARMACOLOGICAL STUDY |
| 2020 | C. M. Gill, T. E. Asempa and D. P. Nicolau | Development and Application of a Pragmatic Algorithm to Guide Definitive Carbapenemase Testing to Identify Carbapenemase-Producing Pseudomonas aeruginosa | 10.3390/antibiotics9110738 | NO | NO | IN VITRO STUDY |
| 2020 | M. Hernández-García, M. García-Castillo, S. García-Fernández, J. Melo-Cristino, M. F. Pinto, E. Gonçalves, V. Alves, A. R. Vieira, E. Ramalheira, L. Sancho, J. Diogo, R. Ferreira, T. Silva, C. Chaves, G. Bou, E. Cercenado, M. Delgado-Valverde, A. Oliver, C. Pitart, J. Rodríguez-Lozano, N. Tormo, J. Romano, L. Pássaro, L. Paixão, D. López-Mendoza, J. Díaz-Regañón and R. Cantón | Distinct epidemiology and resistance mechanisms affecting ceftolozane/tazobactam in Pseudomonas aeruginosa isolates recovered from ICU patients in Spain and Portugal depicted by WGS | 10.1093/jac/dkaa430 | NO | NO | IN VITRO STUDY |
| 2020 | A. M. Hujer, S. W. Long, R. J. Olsen, M. A. Taracila, L. J. Rojas, J. M. Musser and R. A. Bonomo | Predicting β-lactam resistance using whole genome sequencing in Klebsiella pneumoniae: the challenge of β-lactamase inhibitors | 10.1016/j.diagmicrobio.2020.115149 | NO | NO | IN VITRO STUDY |
| 2019 | R. M. Humphries and K. Spafford | Letter to the Editor | 10.1093/cid/ciz224 | NO | NO | LETTER |
| 2020 | J. A. Karlowsky, M. A. Hackel, S. K. Bouchillon and D. F. Sahm | In Vitro Activity of WCK 5222 (Cefepime-Zidebactam) against Worldwide Collected Gram-Negative Bacilli Not Susceptible to Carbapenems | 10.1128/aac.01432-20 | NO | NO | IN VITRO STUDY |
| 2020 | M. H. Kollef, Á. Réa-Neto, R. G. Wunderink, C. J. Bruno and E. G. Rhee | Doripenem for treating nosocomial pneumonia and ventilator-associated pneumonia - Authors' reply | 10.1016/s1473-3099(19)30717-0 | NO | NO | LETTER |
| 2020 | L. Li, X. Li, Y. Xia, Y. Chu, H. Zhong, J. Li, P. Liang, Y. Bu, R. Zhao, Y. Liao, P. Yang, X. Lu and S. Jiang | Recommendation of Antimicrobial Dosing Optimization During Continuous Renal Replacement Therapy | 10.3389/fphar.2020.00786 | NO | NO | REVIEW |
| 2020 | T. Lupia, S. Corcione, S. Mornese Pinna and F. G. De Rosa | New cephalosporins for the treatment of pneumonia in internal medicine wards | 10.21037/jtd-20-417 | NO | NO | REVIEW |
| 2020 | C. Mané, C. Delmas, J. Porterie, G. Jourdan, P. Verwaerde, B. Marcheix, D. Concordet, B. Georges, S. Ruiz and P. Gandia | Influence of extracorporeal membrane oxygenation on the pharmacokinetics of ceftolozane/tazobactam: an ex vivo and in vivo study | 10.1186/s12967-020-02381-1 | NO | NO | PHARMACOLOGICAL STUDY |
| 2020 | F. Marchesi, L. Toma, E. G. Di Domenico, I. Cavallo, A. Spadea, G. Prignano, F. Pimpinelli, E. Papa, I. Terrenato, F. Ensoli and A. Mengarelli | Ceftolozane-Tazobactam for Febrile Neutropenia Treatment in Hematologic Malignancy Patients Colonized by Multi-Resistant Enterobacteriaceae: Preliminary Results from a Prospective Cohort Study | 10.4084/mjhid.2020.065 | YES | NO | LETTER |
| 2020 | I. Martin-Loeches, A. Bisanti, E. Diaz and A. Rodriguez | Ceftolozane and tazobactam for the treatment of hospital acquired pneumonia | 10.1080/14787210.2020.1794816 | NO | NO | REVIEW |
| 2020 | I. Martins-Oliveira, B. Pérez-Viso, S. Quintas, A. Silva-Dias, R. Gomes, A. G. Rodrigues, R. Cantón and C. Pina-Vaz | Evaluation of ultra-rapid susceptibility testing of ceftolozane-tazobactam by a flow cytometry assay directly from positive blood cultures | 10.1007/s10096-020-03926-4 | NO | NO | IN VITRO STUDY |
| 2020 | M. Meschiari, I. Franconi, E. Bacca, V. Bianco, G. Orlando, G. Cuomo, A. Bedini and C. Mussini | Ceftazidime/avibactam and ceftolozane/tazobactam for the treatment of extensively drug-resistant Pseudomonas aeruginosa post-neurosurgical infections: three cases and a review of the literature | 10.1007/s15010-020-01539-9 | NO | NO | CASE REPORT |
| 2020 | A. Motos, G. Li Bassi, F. Pagliara, L. Fernandez-Barat, H. Yang, E. A. Xiol, T. Senussi, F. A. Idone, C. Travierso, C. Chiurazzi, R. Amaro, M. Yang, J. Bobi, M. Rigol, D. P. Nicolau, G. Frigola, R. Cabrera, J. Ramirez, P. Pelosi, F. Blasi, M. Antonelli, A. Artigas, J. Vila, M. Kollef and A. Torres | SHORT-TERM EFFECTS OF APPROPRIATE EMPIRICAL ANTIMICROBIAL TREATMENT WITH CEFTOLOZANE/TAZOBACTAM IN A SWINE MODEL OF NOSOCOMIAL PNEUMONIA | 10.1128/aac.01899-20 | NO | NO | PRECLINICAL STUDY |
| 2020 | M. Moussa, M. Abou Chakra, A. Dellis, Y. Moussa and A. Papatsoris | Pharmacotherapeutic advances for recurrent urinary tract infections in women | 10.1080/14656566.2020.1795128 | NO | NO | REVIEW |
| 2020 | X. Mulet, M. Fernández-Esgueva, C. Norte, L. Zamorano, E. Del Barrio-Tofiño and A. Oliver | Validation of MALDI-TOF for the early detection of the ST175 high-risk clone of Pseudomonas aeruginosa in clinical isolates belonging to a Spanish nationwide multicenter study | 10.1016/j.eimc.2020.05.022 | NO | NO | IN VITRO STUDY |
| 2020 | P. J. Nolan, R. Jain, L. Cohen, J. D. Finklea and T. T. Smith | In vitro activity of ceftolozane-tazobactam and ceftazidime-avibactam against Pseudomonas aeruginosa isolated from patients with cystic fibrosis | 10.1016/j.diagmicrobio.2020.115204 | NO | NO | IN VITRO STUDY |
| 2020 | J. N. O'Donnell, M. R. Bidell and T. P. Lodise | Approach to the Treatment of Patients with Serious Multidrug-Resistant Pseudomonas aeruginosa Infections | 10.1002/phar.2449 | NO | NO | REVIEW |
| 2020 | Z. R. Palacios-Baena, L. Valiente de Santis, N. Maldonado, C. M. Rosso-Fernández, I. Borreguero, C. Herrero-Rodríguez, S. López-Cárdenas, F. J. Martínez-Marcos, A. Martín-Aspas, P. Jiménez-Aguilar, J. J. Castón, F. Anguita-Santos, G. Ojeda-Burgos, M. P. Aznarte-Padial, J. Praena-Segovia, J. E. Corzo-Delgado, M. Esteban-Moreno, J. Rodríguez-Baño and P. Retamar | Quasiexperimental intervention study protocol to optimise the use of new antibiotics in Spain: the NEW_SAFE project | 10.1136/bmjopen-2019-035460 | NO | NO | OUT OF TOPIC |
| 2020 | K. M. Papp-Wallace, A. R. Mack, M. A. Taracila and R. A. Bonomo | Resistance to Novel β-Lactam-β-Lactamase Inhibitor Combinations: The "Price of Progress" | 10.1016/j.idc.2020.05.001 | NO | NO | REVIEW |
| 2020 | M. A. Pfaller, D. Shortridge, S. J. R. Arends, L. R. Duncan, J. M. Streit and R. K. Flamm | Activity of ceftolozane-tazobactam and comparators when tested against bacterial surveillance isolates collected from patients at risk of infections caused by resistant gram-negative pathogens | 10.1016/j.diagmicrobio.2020.115101 | NO | NO | IN VITRO STUDY |
| 2020 | S. Pungcharoenkijkul, J. Traipattanakul, S. Thunyaharn and W. Santimaleeworagun | Antimicrobials as Single and Combination Therapy for Colistin-Resistant Pseudomonas aeruginosa at a University Hospital in Thailand | 10.3390/antibiotics9080475 | NO | NO | IN VITRO STUDY |
| 2020 | W. C. Putnam, R. R. Kallem, V. Edpuganti, I. Subramaniyan and R. G. Hall, 2nd | Development and validation of a quantitative LC-MS/MS method for the simultaneous determination of ceftolozane and tazobactam in human plasma and urine | 10.1016/j.jchromb.2020.122354 | NO | NO | IN VITRO STUDY |
| 2020 | C. A. Pybus, C. Felder-Scott, V. Obuekwe and D. E. Greenberg | Cefiderocol Retains Anti-Biofilm Activity in Multi-Drug Resistant Gram-Negative Pathogens | 10.1128/aac.01194-20 | NO | NO | IN VITRO STUDY |
| 2020 | M. T. Romano, S. Premraj, J. M. Bray and L. C. Murillo | Ceftolozane/tazobactam for pulmonary exacerbation in a 63-year-old cystic fibrosis patient with renal insufficiency and an elevated MIC to Pseudomonas aeruginosa | 10.1016/j.idcr.2020.e00830 | NO | NO | CASE REPORT |
| 2020 | Z. Rubic, S. Soprek, M. Jelic, A. Novak, I. Goic-Barisic, M. Radic, A. Tambic-Andrasevic and M. Tonkic | Molecular Characterization of β-Lactam Resistance and Antimicrobial Susceptibility to Possible Therapeutic Options of AmpC-Producing Multidrug-Resistant Proteus mirabilis in a University Hospital of Split, Croatia | 10.1089/mdr.2020.0002 | NO | NO | IN VITRO STUDY |
| 2020 | M. Sabet, Z. Tarazi and D. C. Griffith | In Vivo Activity of QPX7728, an Ultrabroad-Spectrum Beta-Lactamase Inhibitor, in Combination with Beta-Lactams against Carbapenem-Resistant Klebsiella pneumoniae | 10.1128/aac.01267-20 | NO | NO | PRECLINICAL STUDY |
| 2020 | H. S. Sader, C. G. Carvalhaes, L. R. Duncan, R. K. Flamm and D. Shortridge | Susceptibility trends of ceftolozane/tazobactam and comparators when tested against European Gram-negative bacterial surveillance isolates collected during 2012-18 | 10.1093/jac/dkaa278 | NO | NO | IN VITRO STUDY |
| 2020 | H. S. Sader, C. G. Carvalhaes, J. M. Streit, T. B. Doyle and M. Castanheira | Antimicrobial Activity of Ceftazidime-Avibactam, Ceftolozane-Tazobactam and Comparators Tested Against Pseudomonas aeruginosa and Klebsiella pneumoniae Isolates from United States Medical Centers in 2016-2018 | 10.1089/mdr.2020.0217 | NO | NO | IN VITRO STUDY |
| 2020 | M. Sheffield, D. Nelson, M. O'Neal, A. P. Gould, J. Bouchard, D. Nicolau, J. A. Justo, J. Hucks and P. B. Bookstaver | Use of continuous-infusion ceftolozane/tazobactam for resistant Gram-negative bacterial infections: a retrospective analysis and brief review of the literature | 10.1016/j.ijantimicag.2020.106158 | YES | NO | CONTROL GROUP NOT AVAILABLE |
| 2020 | M. A. Sid Ahmed, F. A. Khan, A. A. Sultan, B. Söderquist, E. B. Ibrahim, J. Jass and A. S. Omrani | β-lactamase-mediated resistance in MDR-Pseudomonas aeruginosa from Qatar | 10.1186/s13756-020-00838-y | NO | NO | IN VITRO STUDY |
| 2020 | F. B. Sime, M. Lassig-Smith, T. Starr, J. Stuart, S. Pandey, S. L. Parker, S. C. Wallis, J. Lipman and J. A. Roberts | Cerebrospinal fluid penetration of ceftolozane/tazobactam in critically ill patients with an indwelling external ventricular drain | 10.1128/aac.01698-20 | NO | NO | PHARMACOLOGICAL STUDY |
| 2020 | C. L. Slater, J. Winogrodzki, P. A. Fraile-Ribot, A. Oliver, M. Khajehpour and B. L. Mark | Adding Insult to Injury: Mechanistic Basis for How AmpC Mutations Allow Pseudomonas aeruginosa To Accelerate Cephalosporin Hydrolysis and Evade Avibactam | 10.1128/aac.00894-20 | NO | NO | IN VITRO STUDY |
| 2020 | P. D. Tamma, S. Beisken, Y. Bergman, A. E. Posch, E. Avdic, S. L. Sharara, S. E. Cosgrove and P. J. Simner | Modifiable Risk Factors for the Emergence of Ceftolozane-Tazobactam Resistance | 10.1093/cid/ciaa1306 | NO | NO | IN VITRO STUDY |
| 2020 | H. J. Tang and C. C. Lai | Doripenem for treating nosocomial pneumonia and ventilator-associated pneumonia | 10.1016/s1473-3099(19)30693-0 | NO | NO | LETTER |
| 2020 | A. K. Thabit | Antibiotics in the Biliary Tract: A Review of the Pharmacokinetics and Clinical Outcomes of Antibiotics Penetrating the Bile and Gallbladder Wall | 10.1002/phar.2431 | NO | NO | REVIEW |
| 2020 | S. A. Winans, R. L. Guerrero-Wooley, S. H. Park, G. Hino, Jr. and S. C. Forland | Continuous infusion of ceftolozane-tazobactam resulted in high cerebrospinal fluid concentrations of ceftolozane in a patient with multidrug-resistant Pseudomonas aeruginosa meningitis | 10.1007/s15010-020-01510-8 | NO | NO | CASE REPORT |
| 2020 | D. Yahav, C. G. Giske, A. Grāmatniece, H. Abodakpi, V. H. Tam and L. Leibovici | New β-Lactam-β-Lactamase Inhibitor Combinations | 10.1128/cmr.00115-20 | NO | NO | REVIEW |
| 2020 | Z. Zhang, Y. T. Patel, J. Fiedler-Kelly, H. P. Feng, C. J. Bruno and W. Gao | Population Pharmacokinetic Analysis for Plasma and Epithelial Lining Fluid Ceftolozane/Tazobactam Concentrations in Patients With Ventilated Nosocomial Pneumonia | 10.1002/jcph.1733 | NO | NO | PHARMACOLOGICAL STUDY |
| 2019 | M. R. Jacobs, A. M. Abdelhamed, C. E. Good, D. D. Rhoads, K. M. Hujer, A. M. Hujer, T. N. Domitrovic, S. D. Rudin, S. S. Richter, D. van Duin, B. N. Kreiswirth, C. Greco, D. E. Fouts and R. A. Bonomo | ARGONAUT-I: Activity of cefiderocol (s-649266), a siderophore cephalosporin, against gram-negative bacteria, including carbapenem-resistant nonfermenters and enterobacteriaceae with defined extended-spectrum β-lactamases and carbapenemases | 10.1128/AAC.01801-18 | NO | NO | IN VITRO STUDY |
| 2019 | J. F. Timsit, J. A. Huntington, R. G. Wunderink, N. Shime, M. Kollef, Ü. Kivistik, M. Nováček, A. Réa-Neto, I. Martin-Loeches, B. Yu, C. J. Bruno, E. Jensen, J. R. Butterton, D. J. Wolf and E. G. Rhee | Ceftolozane/tazobactam (C/T) vs meropenem (MEM) in patients (pts) with ventilated hospital-acquired pneumonia (vHAP) - subset analysis of the ASPECT-NP randomized, controlled, phase 3 trial | 10.1186/s40635-019-0265-y | YES | NO | CONFERENCE PROCEEDINGS |
| 2019 | D. Shortridge, M. A. Pfaller, S. J. R. Arends, J. Raddatz, D. D. Depestel and R. K. Flamm | Comparison of the in Vitro Susceptibility of Ceftolozane-Tazobactam with the Cumulative Susceptibility Rates of Standard Antibiotic Combinations When Tested against Pseudomonas aeruginosa from ICU Patients with Bloodstream Infections or Pneumonia | 10.1093/ofid/ofz240 | NO | NO | IN VITRO STUDY |
| 2019 | I. Martin-Loeches, C. J. Bruno, N. Shime, R. G. Wunderink, M. Kollef, Ü. Kivistik, M. Nováček, A. Réa-Neto, J. F. Timsit, B. Yu, J. A. Huntington, E. Jensen, J. R. Butterton, E. G. Rhee and A. F. Shorr | Outcomes in patients with ventilated nosocomial pneumonia (NP) and organ failure treated with ceftolozane/tazobactam (C/T) vs meropenem (MER) - analysis of the ASPECT-NP randomized, controlled trial | 10.1186/s40635-019-0265-y | YES | NO | CONFERENCE PROCEEDINGS |
| 2019 | A. Khan, T. T. Tran, R. Rios, B. Hanson, W. C. Shropshire, Z. Sun, L. Diaz, A. Q. Dinh, A. Wanger, L. Ostrosky-Zeichner, T. Palzkill, C. A. Arias and W. R. Miller | Extensively Drug-Resistant Pseudomonas aeruginosa ST309 Harboring Tandem Guiana Extended Spectrum β-Lactamase Enzymes: A Newly Emerging Threat in the United States | 10.1093/ofid/ofz273 | NO | NO | IN VITRO STUDY |
| 2019 | C. Cardoso, F. Faria, R. Cavaco, A. Graça and L. Bento | Carbapenem resistant bacteria infections in a general ICU | 10.1186/s40635-019-0265-y | NO | NO | CONFERENCE PROCEEDINGS |
| 2020 | J. Zhou, J. Yang, F. Hu, K. Gao, J. Sun and J. Yang | Clinical and molecular epidemiologic characteristics of ceftazidime/ avibactam-resistant carbapenem-resistant Klebsiella pneumoniae in a neonatal intensive care unit in China | 10.2147/IDR.S256922 | NO | NO | IN VITRO STUDY |
| 2020 | S. S. Yedu Krishnan, P. R. Roshni and T. Aby | In and out about spontaneous bacterial peritonitis: Special mention on risk factors and resistance |  | NO | NO | REVIEW |
| 2020 | M. Sheffield, D. Nelson, M. O'Neal, A. Gould, J. Bouchard, D. Nicolau, J. A. Justo, J. Hucks and B. Bookstaver | The use of continuous infusion Ceftolozane/tazobactam for resistant gram-negative bacterial infections: A case series | 10.1002/jac5.1204 | NO | NO | CASE SERIES |
| 2020 | M. M. Sfeir | Antimicrobial susceptibility testing for glucose-nonfermenting gram-negative bacteria: The tip of the iceberg | 10.1128/AAC.00011-20 | NO | NO | LETTER |
| 2020 | S. Sadyrbaeva-Dolgova, S. Guijarro-Herrera, B. Cancela-Diez, R. García-Fumero and A. Jimenez-Morales | Real life experience with ceftolozane-tazobactam in tertiary hospital | 10.1007/s11096-019-00945-w | YES | NO | CONFERENCE PROCEEDINGS |
| 2020 | S. Rotea, M. García, B. Feal, C. Fernández, V. Giménez and I. Martín | Intravenous perfusion of ceftolozane-tazobactam using elastomeric infusion pumps | 10.1136/ejhpharm-2020-eahpconf.55 | NO | NO | DATA NOT RELEVANT |
| 2020 | P. Moise, M. Gonzalez, I. Alekseeva, D. Lopez, B. Akrich, B. Akrich, A. DeRyke, M. Hackel and M. Motyl | Assessment of antimicrobial susceptibility of the most common Gram-negative intensive care unit (ICU) respiratory pathogens in deciding antimicrobial therapy | 10.1186/s13054-020-2772-3 | NO | NO | IN VITRO STUDY |
| 2020 | A. A. Mirza, E. J. Rad and P. K. Mohabir | Cystic fibrosis and COVID-19: Care considerations | 10.1016/j.rmcr.2020.101226 | NO | NO | REVIEW |
| 2020 | S. M. Martinez castro, E. B. Bárcena Barreto, E. U. Utrera, J. A. Carbonell, R. F. Ferriols and G. A. Aguilar | Ceftolozane/tazobactam pharmacokinetics in critically ill patients on continuous venovenous hemodiafiltration | 10.1186/s13054-020-2772-3 | NO | NO | PHARMACOLOGICAL STUDY |
| 2020 | M. Kollef, J. F. Timsit, I. Martin-Loeches, R. G. Wunderink, J. A. Huntington, E. Jensen, C. J. Bruno, B. Yu, D. J. Wolf and E. G. Rhee | Outcomes in patients (pts) with failure of initial antibiotic therapy for hospital-acquired/ventilator-associated bacterial pneumonia (HABP/VABP) prior to enrollment in the phase 3 ASPECT-NP trial of ceftolozane/tazobactam (C/T) vs. meropenem (MEM) | 10.1186/s13054-020-2772-3 | YES | NO | ABSTRACT |
| 2020 | J. M. Kidd, K. Abdelraouf and D. P. Nicolau | Efficacy of human-simulated bronchopulmonary exposures of cefepime, zidebactam and the combination (WCK 5222) against MDR Pseudomonas aeruginosa in a neutropenic murine pneumonia model | 10.1093/jac/dkz414 | NO | NO | PRECLINICAL STUDY |
| 2020 | M. Hernández-garcia, C. C. Chaves, J. M. Melo-Cristino, D. S. Silva, A. R. Vieira, M. P. F. Pinto, J. D. Diogo, E. G. Gonçalves, J. R. Romano and R. C. Cantón | Characterization of resistance mechanisms affecting ceftolozane/ tazobactam in Enterobacterales and Pseudomonas aeruginosa ICU isolates using whole genome sequencing (STEP study) | 10.1186/s13054-020-2772-3 | NO | NO | ABSTRACT |
| 2020 | L. Gras Martín, L. López Vinardell, E. Fernández De Gamarra Martínez, J. López-Contreras González, I. Conejo Marín, N. Mas Malagarriga, M. A. Mangues Bafalluy and N. Garin Escrivá | Patterns of use of ceftolozane-tazobactam in a tertiaryhospital | 10.1007/s11096-019-00945-w | NO | NO | ABSTRACT |
| 2020 | L. Gómez-Ganda, P. Lalueza-Broto, Á. G. Arévalo-Bernabé, A. Rey-Pérez, J. Baena-Caparrós, L. Domenech-Moral and M. Baguena-Martínez | Evolution of antimicrobial consumption in a trauma intensive care unit using defined daily doses per 100 occupied bed days | 10.1136/ejhpharm-2020-eahpconf.276 | NO | NO | ABSTRACT |
| 2020 | L. Gambitta, S. Campbell Davies, E. Galfrascoli, E. Calzavara and V. Curci | Antibiotic prescription through motivated request: Clinical pharmacy tool to improve appropriateness and limit resistant bacterial strains. A follow-up after a year of monitoring in a local hospital | 10.1136/ejhpharm-2020-eahpconf.133 | NO | NO | ABSTRACT |
| 2020 | M. Falcone, G. Tiseo, A. Antonelli, C. Giordano, V. Di Pilato, P. Bertolucci, E. M. Parisio, A. Leonildi, N. Aiezza, I. Baccani, E. Tagliaferri, L. Righi, S. Forni, S. Sani, M. T. Mechi, F. Pieralli, S. Barnini, G. M. Rossolini and F. Menichetti | Clinical features and outcomes of bloodstream infections caused by New Delhi metallo-β-lactamase-producing enterobacterales during a regional outbreak | 10.1093/ofid/ofaa011 | YES | NO | NO C/T DATA |
| 2020 | S. D. Da Costa Júnior, W. R. C. Da Silva, A. M. C. M. Da Silva, M. A. V. Maciel and I. M. F. Cavalcanti | Synergistic Effect between Usnic Acid and Polymyxin B against Resistant Clinical Isolates of Pseudomonas aeruginosa | 10.1155/2020/9852145 | NO | NO | IN VITRO STUDY |
| 2020 | S. Anwar, S. Acharya, S. Thapa, J. Rabadi and N. Mobarakai | Carbapenemase producing Klebsiella pneumoniae (KPC) meningitis from chronic otitis media | 10.1016/j.idcr.2020.e00963 | NO | NO | CASE REPORT |
| 2020 | S. Alosaimy, S. C. J. Jorgensen, A. M. Lagnf, S. Melvin, R. P. Mynatt, T. J. Carlson, K. W. Garey, D. Allen, V. Venugopalan, M. Veve, V. Athans, S. Saw, C. N. Yost, S. L. Davis and M. J. Rybak | Real-world multicenter analysis of clinical outcomes and safety of meropenem-vaborbactam in patients treated for serious gram-negative bacterial infections | 10.1093/ofid/ofaa051 | NO | NO | NO C/T DATA |
| 2020 |  | 25th EAHP Congress |  | NO | NO | ABSTRACT |
| 2020 | F. F. Tuon, J. Cieslinski, S. D. S. Rodrigues, F. B. Serra and M. D. N. D. Paula | Evaluation of in vitro activity of ceftolozane–tazobactam against recent clinical bacterial isolates from Brazil – the EM200 study | 10.1016/j.bjid.2020.04.004 | NO | NO | IN VITRO STUDY |
| 2020 | P. Srivastava and K. Sivashanmugam | Combinatorial Drug Therapy for Controlling Pseudomonas aeruginosa and Its Association With Chronic Condition of Diabetic Foot Ulcer | 10.1177/1534734619873785 | NO | NO | REVIEW |
| 2020 | K. V. I. Rolston, B. Gerges, S. Shelburne, S. L. Aitken, I. Raad and R. A. Prince | Activity of cefiderocol and comparators against isolates from cancer patients | 10.1128/AAC.01955-19 | NO | NO | IN VITRO STUDY |
| 2020 | S. Palwe, B. Veeraraghavan, H. Periasamy, K. Khobragade and A. S. Kharat | Unorthodox parenteral β-lactam and β-lactamase inhibitor combinations: Flouting antimicrobial stewardship and compromising patient care | 10.1128/AAC.00168-20 | NO | NO | REVIEW |
| 2020 | S. Palwe, K. Khobragade and A. S. Kharat | Preserving the Dwindling ß-lactams-Based Empiric Therapy Options for Gram-Negative Infections in Challenging Resistance Scenario: Lessons Learned and Way Forward | 10.1089/mdr.2019.0195 | NO | NO | REVIEW |
| 2020 | I. Osipov, V. Ivanov, G. Salogub, L. Girshova, N. Stepanova and M. Aksenov | Ceftolozane/tazobactam in the treatment of nosocomial infections associated with XDR-strains of pseudomonas aeruginosa among patients with acute myeloid leukaemia | 10.1097/HS9.0000000000000404 | NO | NO | CASE SERIES |
| 2020 | H. G. Griffith, K. Dantuluri, C. Thurm, D. J. Williams, R. Banerjee, L. M. Howard and C. G. Grijalva | Considerable variability in antibiotic use among US children's hospitals in 2017-2018 | 10.1017/ice.2019.373 | NO | NO | OUT OF TOPIC |
| 2020 | F. Gómez De Rueda, B. Cancela Díez, E. Tena Sempere María, M. Cárdenas Antón and A. Gálvez Del Postigo | Ceftolozane/tazobactam as a treatment for multidrug-resistant P. Aeruginosa in a ventricle-peritoneal shunt |  | NO | NO | CASE REPORT |
| 2020 | D. R. Giacobbe and I. Karaiskos | Stewardship of antibiotics for multidrug-resistant gram-negative bacteria | 10.3390/antibiotics9040206 | NO | NO | EDITORIAL |
| 2020 | I. M. Ghazi, W. S. El Nekidy, R. Asay, P. Fingmori, A. Knarr and M. Awad | Simultaneous administration of imipenem/ cilastatin/relebactam with selected intravenous antimicrobials, a stewardship approach | 10.1371/journal.pone.0233335 | NO | NO | PHARMACOLOGICAL STUDY |
| 2020 | R. A. Bonnin, A. B. Jousset, A. Chiarelli, C. Emeraud, P. Glaser, T. Naas and L. Dortet | Emergence of new non-clonal group 258 high-risk clones among Klebsiella pneumoniae Carbapenemase-Producing K. Pneumoniae Isolates, France | 10.3201/EID2606.191517 | NO | NO | IN VITRO STUDY |
| 2020 | S. Avija, V. Kommineni, S. Latha, R. Pavani and M. Bhagavan Raju | Development of a stability indicating hplc method forsimultaneous estimation of ceftolozane and tazobactamand its validation as per ICH guidelines |  | NO | NO | OUT OF TOPIC |
| 2020 | A. Alvarez-Buylla, M. Allen, D. Betts, S. Bennett, I. Monahan and T. Planche | Multicentre study of the in vitro activity of ceftolozane/tazobactam and other commonly used antibiotics against Pseudomonas aeruginosa isolates from patients in the UK | 10.1093/jacamr/dlaa024 | NO | NO | IN VITRO STUDY |
| 2020 | R. Zaragoza, P. Vidal-Cortés, G. Aguilar, M. Borges, E. Diaz, R. Ferrer, E. Maseda, M. Nieto, F. X. Nuvials, P. Ramirez, A. Rodriguez, C. Soriano, J. Veganzones and I. Martín-Loeches | Update of the treatment of nosocomial pneumonia in the ICU | 10.1186/s13054-020-03091-2 | NO | NO | REVIEW |
| 2020 | G. Wareth, J. Linde, P. Hammer, N. H. Nguyen, T. N. M. Nguyen, W. D. Splettstoesser, O. Makarewicz, H. Neubauer, L. D. Sprague and M. W. Pletz | Phenotypic and WGS-derived antimicrobial resistance profiles of clinical and non-clinical Acinetobacter baumannii isolates from Germany and Vietnam | 10.1016/j.ijantimicag.2020.106127 | NO | NO | IN VITRO STUDY |
| 2020 | E. Wallenburg, R. ter Heine, J. A. Schouten and R. J. M. Brüggemann | Personalised antimicrobial dosing: standing on the shoulders of giants | 10.1016/j.ijantimicag.2020.106062 | NO | NO | REVIEW |
| 2020 | T. ten Doesschate, T. W. van der Vaart, J. A. A. Damen, M. J. M. Bonten and C. H. van Werkhoven | Carbapenem-alternative strategies for complicated urinary tract infections: A systematic review of randomized controlled trials | 10.1016/j.jinf.2020.08.008 | NO | NO | SYSTEMATIC REVIEW |
| 2020 | N. R. Shively, M. A. Moffa, K. T. Paul, E. J. Wodusky, B. A. Schipani, S. L. Cuccaro, M. S. Harmanos, M. S. Cratty, B. N. Chamovitz and T. L. Walsh | Impact of a telehealth-based antimicrobial stewardship program in a community hospital health system | 10.1093/cid/ciz878 | NO | NO | OUT OF TOPIC |
| 2020 | A. Sharma, K. A. Wahby, M. Inany and S. J. Lee | Use of phenytoin for treatment of tacrolimus toxicity with superimposed sepsis | 10.1136/bcr-2020-234839 | NO | NO | CASE REPORT |
| 2020 | E. Scaltriti, G. Piccinelli, S. Corbellini, A. Caruso, N. Latronico and M. A. De Francesco | Detection of a hypermucoviscous Klebsiella pneumoniae co-producing NDM-5 and OXA-48 carbapenemases with sequence type 383, Brescia, Italy | 10.1016/j.ijantimicag.2020.106130 | NO | NO | LETTER |
| 2020 | M. C. Safir, S. M. Bhavnani, C. M. Slover, P. G. Ambrose and C. M. Rubino | Antibacterial drug development: A new approach is needed for the field to survive and thrive | 10.3390/antibiotics9070412 | NO | NO | REVIEW |
| 2020 | H. S. Sader, M. Castanheira, J. M. Streit, C. G. Carvalhaes and R. E. Mendes | Frequency and antimicrobial susceptibility of bacteria causing bloodstream infections in pediatric patients from United States (US) medical centers (2014–2018): therapeutic options for multidrug-resistant bacteria | 10.1016/j.diagmicrobio.2020.115108 | NO | NO | IN VITRO STUDY |
| 2020 | R. S. Rekha, H. Karadottir, S. Ahmed, G. H. Gudmundsson, B. Agerberth and P. Bergman | Innate effector systems in primary human macrophages sensitize multidrug-resistant klebsiella pneumoniae to antibiotics | 10.1128/IAI.00186-20 | NO | NO | IN VITRO STUDY |
| 2020 | A. Protić, M. Bura and K. Juričić | A 23-year-old man with left lung atelectasis treated with a targeted segmental recruitment maneuver: A case report | 10.1186/s13256-020-02409-6 | NO | NO | CASE REPORT |
| 2020 | B. A. Pontefract, H. T. Ho, A. Crain, M. K. Kharel and S. E. Nybo | Drugs for Gram-negative bugs from 2010-2019: A decade in review | 10.1093/ofid/ofaa276 | NO | NO | REVIEW |
| 2020 | L. Poirel, X. Vuillemin, M. Juhas, A. Masseron, U. Bechtel-Grosch, S. Tiziani, S. Mancini and P. Nordmann | KPC-50 confers resistance to ceftazidime-avibactam associated with reduced carbapenemase activity | 10.1128/AAC.00321-20 | NO | NO | CASE REPORT |
| 2020 | M. PÉRez-VÁZquez, P. J. Sola-Campoy, Á. M. Zurita, A. ÁVila, F. GÓMez-Bertomeu, S. SolÍS, L. LÓPez-Urrutia, E. M. GÓNzalez-BarberÁ, E. Cercenado, V. Bautista, N. Lara, B. Aracil, A. Oliver, J. Campos and J. Oteo-Iglesias | Carbapenemase-producing Pseudomonas aeruginosa in Spain: interregional dissemination of the high-risk clones ST175 and ST244 carrying blaVIM-2, blaVIM-1, blaIMP-8, blaVIM-20 and blaKPC-2 | 10.1016/j.ijantimicag.2020.106026 | NO | NO | IN VITRO STUDY |
| 2020 | M. Penven, A. Lalieu, A. Boruchowicz, M. Paluch, T. Diedrich, G. Dewulf and C. Cattoen | Bacteremia caused by Elizabethkingia miricola in a patient with acute pancreatitis and peritoneal dialysis | 10.1016/j.medmal.2020.01.009 | NO | NO | CASE REPORT |
| 2020 | S. Octavia, K. L. Chew, R. T. P. Lin and J. W. P. Teo | Multidrug-resistant Salmonella enterica serovar London carrying blaNDM-1 encoding plasmid from Singapore | 10.1016/j.cmi.2020.01.033 | NO | NO | CASE REPORT |
| 2020 | J. D. Lutgring, R. Balbuena, N. Reese, S. E. Gilbert, U. Ansari, A. Bhatnagar, S. Boyd, D. Campbell, J. Cochran, J. Haynie, J. Ilutsik, C. Longo, S. Swint, J. K. Rasheed, A. C. Brown and M. Karlsson | Antibiotic susceptibility of NDM-producing enterobacterales collected in the United States in 2017 and 2018 | 10.1128/AAC.00499-20 | NO | NO | IN VITRO STUDY |
| 2020 | Y. R. Lee and S. Yeo | Cefiderocol, a New Siderophore Cephalosporin for the Treatment of Complicated Urinary Tract Infections Caused by Multidrug-Resistant Pathogens: Preclinical and Clinical Pharmacokinetics, Pharmacodynamics, Efficacy and Safety | 10.1007/s40261-020-00955-x | NO | NO | REVIEW |
| 2020 | M. Kresken, M. Korte-Berwanger, S. G. Gatermann, Y. Pfeifer, N. Pfennigwerth, H. Seifert and G. Werner | In vitro activity of cefiderocol against aerobic Gram-negative bacterial pathogens from Germany | 10.1016/j.ijantimicag.2020.106128 | NO | NO | IN VITRO STUDY |
| 2020 | T. Kostyanev, M. N. Nguyen, R. Markovska, P. Stankova, B. B. Xavier, C. Lammens, Y. Marteva-Proevska, T. Velinov, R. Cantón, H. Goossens and S. Malhotra-Kumar | Emergence of ST654 Pseudomonas aeruginosa co-harbouring blaNDM-1 and blaGES-5 in novel class I integron In1884 from Bulgaria | 10.1016/j.jgar.2020.06.008 | NO | NO | LETTER |
| 2020 | M. Kosecka-Strojek, E. Sadowy, I. Gawryszewska, J. Klepacka, T. Tomasik, M. Michalik, W. Hryniewicz and J. Miedzobrodzki | Emergence of linezolid-resistant Staphylococcus epidermidis in the tertiary children’s hospital in Cracow, Poland | 10.1007/s10096-020-03893-w | NO | NO | IN VITRO STUDY |
| 2020 | A. Jorda and M. Zeitlinger | Preclinical Pharmacokinetic/Pharmacodynamic Studies and Clinical Trials in the Drug Development Process of EMA-Approved Antibacterial Agents: A Review | 10.1007/s40262-020-00892-0 | NO | NO | REVIEW |
| 2020 | M. Iannaccone, M. Boattini, G. Bianco, R. Cavallo and C. Costa | Meropenem/vaborbactam-based combinations against KPC-producing Klebsiella pneumoniae and multidrug-resistant Pseudomonas aeruginosa | 10.1016/j.ijantimicag.2020.106066 | NO | NO | IN VITRO STUDY |
| 2020 | S. J. Hecker, K. R. Reddy, O. Lomovskaya, D. C. Griffith, D. Rubio-Aparicio, K. Nelson, R. Tsivkovski, D. Sun, M. Sabet, Z. Tarazi, J. Parkinson, M. Totrov, S. H. Boyer, T. W. Glinka, O. A. Pemberton, Y. Chen and M. N. Dudley | Discovery of Cyclic Boronic Acid QPX7728, an Ultrabroad-Spectrum Inhibitor of Serine and Metallo-β-lactamases | 10.1021/acs.jmedchem.9b01976 | NO | NO | OUT OF TOPIC |
| 2020 | J. A. Goldberg, H. Nguyen, V. Kumar, E. J. Spencer, D. Hoyer, E. K. Marshall, A. Cmolik, M. O'Shea, S. H. Marshall, A. M. Hujer, A. M. Hujer, K. M. Hujer, K. M. Hujer, S. D. Rudin, S. D. Rudin, T. N. Domitrovic, T. N. Domitrovic, C. R. Bethel, K. M. Papp-Wallace, K. M. Papp-Wallace, K. M. Papp-Wallace, L. K. Logan, L. K. Logan, F. Perez, F. Perez, F. Perez, M. R. Jacobs, M. R. Jacobs, D. Van Duin, B. M. Kreiswirth, R. A. Bonomo, R. A. Bonomo, R. A. Bonomo, R. A. Bonomo, R. A. Bonomo, R. A. Bonomo, M. S. Plummer and F. Van Den Akker | A γ-Lactam Siderophore Antibiotic Effective against Multidrug-Resistant Gram-Negative Bacilli | 10.1021/acs.jmedchem.0c00255 | NO | NO | CASE REPORT |
| 2020 | N. Godefroy, C. Meloni, H. Junot, L. Drieux, C. Luyt and A. Bleibtreu |  | 10.1016/j.medmal.2020.06.081 | NO | NO | NOT IN ENGLISH |
| 2020 | M. G. Chai, M. O. Cotta, M. H. Abdul-Aziz and J. A. Roberts | What are the current approaches to optimising antimicrobial dosing in the intensive care unit? | 10.3390/pharmaceutics12070638 | NO | NO | REVIEW |
| 2020 | M. Carugati, A. Piazza, A. M. Peri, L. Cariani, M. Brilli, D. Girelli, D. Di Carlo, A. Gramegna, M. Pappalettera, F. Comandatore, G. Grasselli, A. P. Cantù, M. Arghittu, A. Gori, C. Bandi, F. Blasi and A. Bandera | Fatal respiratory infection due to ST308 VIM-1-producing Pseudomonas aeruginosa in a lung transplant recipient: case report and review of the literature | 10.1186/s12879-020-05338-3 | NO | NO | CASE REPORT |
| 2020 | J. Al Salman, L. Al Dabal, M. Bassetti, W. A. Alfouzan, M. Al Maslamani, B. Alraddadi, A. Elhoufi, M. Enani, F. A. Khamis, E. Mokkadas, I. Romany, A. Somily and S. Kanj | Management of infections caused by WHO critical priority Gram-negative pathogens in Arab countries of the Middle East: a consensus paper | 10.1016/j.ijantimicag.2020.106104 | NO | NO | REVIEW |
| 2020 | W. M. Akram, G. A. Menezes, N. Abbas, W. Ahmad and A. M. Ahmed | Treatment of multi-drug resistant gram-negative bacterial pathogenic infections | 10.22207/JPAM.14.3.02 | NO | NO | REVIEW |
| 2020 | C. Adembri, I. Cappellini and A. Novelli | The role of PK/PD–based strategies to preserve new molecules against multi-drug resistant gram-negative strains | 10.1080/1120009X.2020.1786634 | NO | NO | REVIEW |
| 2020 | J. B. Kopp, H. J. Anders, K. Susztak, M. A. Podestà, G. Remuzzi, F. Hildebrandt and P. Romagnani | Podocytopathies | 10.1038/s41572-020-0196-7 | NO | NO | REVIEW |
| 2020 | J. Gómez-Junyent, R. Rigo-Bonnin, E. Benavent, L. Soldevila, A. Padullés, X. Cabo, F. Tubau, J. Ariza and O. Murillo | Efficacy and Therapeutic Drug Monitoring of Continuous Beta-Lactam Infusion for Osteoarticular Infections Caused by Fluoroquinolone-Resistant Pseudomonas aeruginosa: A Prospective Cohort Study | 10.1007/s13318-020-00625-5 | YES | NO | AGGREGATED DATA |
| 2021 | M. Bassetti, E. Righi and M. Akova | Gram-Negative Infections | 10.1007/978-3-030-57317-1_12 | NO | NO | BOOK |
| 2020 | J. Arca-Suárez, C. Lasarte-Monterrubio, B. K. Rodiño-Janeiro, G. Cabot, J. C. Vázquez-Ucha, M. Rodríguez-Iglesias, F. Galán-Sánchez, A. Beceiro, C. González-Bello, A. Oliver and G. Bou | Molecular mechanisms driving the in vivo development of OXA-10-mediated resistance to ceftolozane/tazobactam and ceftazidime/avibactam during treatment of XDR Pseudomonas aeruginosa infections | 10.1093/jac/dkaa396 | NO | NO | IN VITRO STUDY |
| 2020 | F. Markotic, S. Grgic, G. Poropat, A. Fox, D. Nikolova, K. Vukojevic, J. C. Jakobsen and C. Gluud | Antibiotics for adults with acute cholecystitis or acute cholangitis or both | 10.1002/14651858.CD013646 | NO | NO | PROTOCOL |

Abstract 4% (n=51), Aggregated data 0,3% (n=3), Book 0,8% (n=9), Case report 9,6% (n=110), Case series 0,8% (n=9), Comment 1,7% (n=19), Conference Proceedings 1,7% (n=19), Cost-effectiveness analysis 0,9% (n=11), Data not relevant 0,4% (n=4), Editorial 1,6% (n=18), Guidelines 0,6% (n=7), In vitro study 28,7% (n=328), Letter 3,8% (n=43), Meta-analysis 0,3% (n=3), Monograph 0,2% (n=2), No C/T data 0,4% (n=54), No control group 0,08% (n=1), Not comparable data 0,4% (n=5), not found 0,2% (n=2), not in English 0,6% (n=7), Out of topic 5,8% (n=66), Pharmacological study 6,2% (n=71), Preclinical study 1,7% (n=19), Protocol 0,9% (n=10), Review 27% (n=309), Systematic review 0,7% (n=8).
