# Supplementary material for: Ceftolozane-Tazobactam Combination Therapy Compared to Ceftolozane-Tazobactam Monotherapy for the Treatment of Severe Infections: A Systematic Review and Meta-Analysis
Source: Antibiotics (Basel). 2021 Jan 15;10(1):79. doi: 10.3390/antibiotics10010079 (PMC7830767; doi:10.3390/antibiotics10010079)
Supplement: Supplementary file 1 [file antibiotics-10-00079-s001.zip › supplementary tables_/Table S2.docx]

**Table S2.** Quality assessment in a systematic review of cohort studies, using the New Castle-Ottawa Scale. References (Ref.) are available from the main document.

| **COHORT STUDIES** | | | | |
| --- | --- | --- | --- | --- |
| **Study** | **Ref.** | **Selection** | **Comparability** | **Outcome** |
| Haidar (2017) | **[14]** | **** |  | **** |
| Xipell (2018) | **[16]** | **** |  | **** |
| Bassetti (2018) | **[17]** | **** | **** | **** |
| Díaz-Cañestro (2018) | **[18]** | **** |  | **** |
| Rodríguez-Núñez (2019) | **[19]** | **** | **** | **** |
| Gerlach (2019) | **[20]** | **** | **** | **** |
| Bassetti (2020) | **[21]** | **** | **** | **** |
| **CASE-CONTROL STUDIES** | | | | |
| **Study** | **Ref.** | **Selection** | **Comparability** | **Exposure** |
| Fernández-Cruz (2018) | **[15]** | **** | **** | **** |

**Risk of bias legend**

*Cohort studies*

A study can be awarded with a star (****) for each item within the Selection (four stars), Comparability (two stars) and Outcome (three stars), for a maximum of cumulative nine stars.
*Case-Control studies*

A study can be awarded a maximum of one star (****) for each numbered item within the Selection and Exposure categories. A maximum of two stars can be given for Comparability
